# Protein-Predicted Obesity Phenotypes and Cardiovascular Events: A Secondary Analysis of UK Biobank Proteomics Data

**DOI:** 10.3390/proteomes13040051

**Published:** 2025-10-09

**Authors:** Chang Liu, Bojung Seo, Qin Hui, Peter W. F. Wilson, Arshed A. Quyyumi, Yan V. Sun

**Affiliations:** 1 Department of Epidemiology, Emory University Rollins School of Public Health, Atlanta, GA 30322, USA; chang.liu2@emory.edu (C.L.); bjseo2@gmail.com (B.S.); qhui@emory.edu (Q.H.); pwwilso@emory.edu (P.W.F.W.); 2 Atlanta VA Healthcare System, Decatur, GA 30033, USA; 3 Division of Cardiology, Department of Medicine, Emory University School of Medicine, Atlanta, GA 30322, USA; aquyyum@emory.edu

**Keywords:** obesity, proteomics, MACE, prediction

## Abstract

Background: Proteomic profiling may improve the understanding of obesity and cardiovascular risk prediction. This study explores the use of protein-predicted scores for body mass index (PPS_BMI_), body fat percentage (PPS_BFP_), and waist–hip ratio (PPS_WHR_) to estimate risk for major adverse cardiovascular events (MACEs). Methods: We used data from the UK Biobank with proteome profiling. PPS_BMI_, PPS_BFP_, and PPS_WHR_ were derived using the LASSO algorithm. The association between these protein scores and incident MACEs was evaluated using a competing risk model. Results: Strong to moderate correlations were observed between protein-predicted obesity phenotypes and their measured counterparts (R^2^: BMI = 0.78, BFP = 0.85, WHR = 0.63). Each standard deviation increment of PPS_BFP_ and PPS_WHR_, but not PPS_BMI_, was associated with greater risk of MACEs (hazard ratio [HR] 1.25, 95% CI 1.14–1.38, *p* < 0.0001; HR 1.15, 95% CI 1.06–1.24, *p* = 0.001, respectively). For predicting MACEs, compared with the PREVENT equation (C statistic 0.694), the models adjusted for only age, sex, current smoking, and protein scores showed comparable performance (C statistics 0.684–0.688). Conclusion: Protein-predicted scores of obesity showed strong independent associations and predictive performance for MACEs, suggesting they may capture additional biological risk beyond anthropometry. These scores may complement existing risk models by providing a biologically informed approach to assessing obesity-related cardiovascular risk and improving risk stratification.

## 1. Introduction

Over the past two decades, the prevalence of obesity, defined as a body mass index (BMI) greater than 30 kg/m^2^, has grown inexorably, and the age-adjusted prevalence of obesity among U.S. adults has reached 42.4% [1]. Obesity is a well-established modifiable risk factor for adverse cardiovascular events [2]. While BMI has been used as a simple and standard metric to quantify adiposity, its limitations in capturing body composition nuances have prompted a search for more comprehensive measures of obesity risk [3]. This has led to the emergence of body fat percentage (BFP) and waist–hip ratio (WHR), which can offer different assessments of adiposity and its impact on health [4,5].

Proteomics is an emerging field that holds promise to unravel the complex molecular mechanisms underlying various phenotypes. Advancements in the high-throughput technology of proteomics have enabled a comprehensive exploration of the molecular landscape underlying obesity [6]. Using proteomic markers to understand obesity-related phenotypes and their potential role in the prediction of cardiovascular events remains relatively unexplored. Recent studies have shown that protein risk scores that incorporate proteomic profiles can enhance prediction of adverse cardiovascular events in both primary and secondary event populations, outperforming traditional risk factors [7,8]. Leveraging obesity-associated proteomics improves our understanding of obesity at the molecular level, and thus may enable more precise estimates of adverse cardiovascular outcomes beyond directly measured phenotypes. In our study, we conducted a secondary analysis of existing proteomic data from the UK Biobank to identify proteomic markers and scores associated with obesity-related phenotypes, such as BMI, BFP, and WHR, and assessed the associations and predictive performance of these proteomic scores for incident major adverse cardiovascular events (MACEs).

## 2. Materials and Methods

The UK Biobank (UKB) is a large-scale biomedical database, with the study design and cohort profile having been described previously [9]. Established in 2006, the UKB recruited approximately half a million participants aged between 40 and 69 years old from the United Kingdom. Participants completed standard questionnaires and provided detailed information about medical conditions, lifestyle, environment, physical measurements, and biological measures. The UKB cohort was linked to Hospital Episode Statistics data for hospital admissions and primary care data, and a death registry included date of death and both primary and secondary causes of death. All first occurrences of disease and cause of death were mapped to the International Classification of Diseases, Tenth Revision (ICD-10), codes [9]. In this study, we conducted a secondary analysis of existing proteomic and phenotypic data made available by the UKB [9]. Proteomic profiling on blood plasma samples was previously performed for 54,219 participants in the UKB using the antibody-based Olink Explore 3072 PEA platform [10]. A total of 2923 distinct proteins were measured [10].

At baseline, demographics and risk factors were collected at enrollment, including age, sex, race and ethnicity, total cholesterol levels, high-density lipoprotein cholesterol (HDL-C) levels, systolic blood pressure, estimated glomerular filtration rate (eGFR) calculated using the 2021 CKD-EPI equation [11], diabetes, smoking status, blood-pressure-lowering medication use, and cholesterol-lowering medication use. Baseline obesity-related phenotypes including BMI, BFP, and WHR were obtained. BFP (%) was measured using the Tanita BC418MA body composition analyzer and was defined as the total mass of fat divided by total body mass, multiplied by 100. WHR was defined as the ratio of waist circumference to hip circumference. Time to MACEs and the subcomponents were defined as the duration from enrollment until the event, loss to follow-up, or the conclusion of follow-up in September 2023. All data were obtained by the UKB study team.

Among the participants with proteomic profiling, a subcohort of participants formed the healthy cohort without prevalent or incident diabetes (ICD-10 codes E10-E14), cardiovascular disease (ICD-10 codes I00-I13, I15, I20-I51, I60-I69), renal disease (ICD-10 codes N17-N23, N25-N29), or cancer. Additionally, participants without prevalent stroke (ICD-10 codes I60, I61, I63, I64) and coronary artery disease (ICD-10 codes I20-I25) were included for the prediction of a MACE, a composite event that included incident ischemic stroke (algorithmically defined, Data-Field 42008), myocardial infarction (MI, ICD-10 codes I21-I23, I25), and cardiovascular death (ICD-10 codes I00-I13, I15, I20-I51, I60-I69).

After excluding participants with >20% missing data across the 2923 proteins, a total of 15,652 participants in the healthy cohort and an additional 24,999 participants without prevalent stroke and coronary artery disease were included in the analysis. A total of 4 proteins with >20% missing data across all samples were excluded, and 2919 proteins remained in the analysis. The missingness of the protein levels were imputed to the minimum value across the samples, assuming they were below the detectable limit. Protein levels were transformed using rank-based inverse normalization, which ranks values, converts them to quantiles, and maps them to the corresponding standard normal z-scores to reduce skewness and the impact of outliers [12].

Cohort characteristics were compared between participants with and without MACEs using a two-sample t-test or Kruskal–Wallis test for continuous variables, and the Chi-squared test or Fisher’s exact test for categorical variables. Bonferroni correction was applied for multiple testing.

The protein scores for obesity-related phenotypes, including BMI, BFP, and WHR, were trained using data from the healthy cohort. To determine the adequate sample size for the training set of these protein scores, we partitioned the healthy cohort randomly into increments of 10%, ranging from 10% to 90%. Each partition was repeated 100 times. Participants were randomly selected from the healthy cohort, and the remaining participants constituted the test set. Within the training set, we applied the least absolute shrinkage and selection operator (LASSO) algorithm using the R package glmnet 4.1-9 with ten-fold cross-validation to identify the proteins that best predicted the measured obesity-related phenotype. Unlike traditional methods that rely on statistical significance, the LASSO algorithm selects proteins based on their capacity to explain the variance in the obesity phenotype, prioritizing their predictive performance within the model. For each obesity-related trait, the LASSO-selected proteins were analyzed for gene ontology pathway enrichment using the R package topGO [13]. Multiple testing corrections for the pathways were performed using false discovery rate (FDR), with an FDR-corrected q value < 0.05 considered significant [14].

Additionally, these selected proteins in the training set were used to compute a weighted protein score, namely, protein-predicted score of BMI (PPS_BMI_), BFP (PPS_BFP_), and WHR (PPS_WHR_). The scores were calculated by summing the protein levels weighted by the LASSO-derived beta coefficients, and subsequently transformed into z-scores with a mean of zero and a standard deviation of one. The performance of the PPS in the test set was evaluated using R^2^ in the linear regression of the PPS against the measured phenotype. We examined the association between each LASSO-selected protein and the obesity-related phenotype using linear regression, and the measured obesity-related phenotype was regressed on the specific protein.

For risk prediction of MACE incidence and its subcomponents, ischemic stroke, myocardial infarction, and cardiovascular death, we utilized the healthy cohort excluding the training set plus the participants without prevalent stroke and coronary artery disease at enrollment. In this cohort, the associations between PPS_BMI_, PPS_BFP_, and PPS_WHR_ and outcomes were estimated using Fine and Gray’s competing risk model, treating death as a competing risk [15]. Three models with hierarchical adjustment were adopted: Model 1 adjusted for age, sex, and race (white vs. other); Model 2 adjusted for the measured obesity-related phenotype (BMI, BFP, or WHR) in addition to Model 1; Model 3 adjusted for additional risk factors in the PREVENT equation [16], including total cholesterol, HDL-C, systolic blood pressure, eGFR, diabetes, current smoking, blood-pressure-lowering medication use, and cholesterol-lowering medication use, in addition to Model 2. The analyses were conducted in the overall cohort and sex-stratified groups. Sex interaction with the protein scores was tested using Model 3. Additionally, a sensitivity analysis was performed after excluding 3736 individuals with prevalent cancer.

C statistics were calculated to evaluate the performance of the protein scores in predicting MACEs using the R package survC1 [17]. Each protein score was evaluated individually and together, in both an unadjusted model and a model adjusted for age, sex, and current smoking. C statistics were also calculated for the PREVENT equation model [16] to compare the predictive performance.

The overall study workflow is shown in Supplemental Figure S1. All data analyses were conducted using R version 4.4.0. Statistical significance was based on *p* values < 0.05. The codes used for data analysis are available at https://github.com/Sun-Epi3-Lab (accessed on 25 September 2025).

## 3. Results

Among 40,651 participants with proteomic data, a total of 4071 (10.0%) developed incident MACEs over a median follow-up of 14.5 (interquartile range 13.7–15.2) years, including 781 (19.2%) incident ischemic stroke, 3096 (76.1%) MI, and 978 (24.0%) cardiovascular death events, as shown in Table 1. After Bonferroni correction (*p* < 0.0025, 0.05 divided by 20 independent tests), participants with incident MACEs were older, more likely to be male, and had a higher prevalence of cardiovascular risk factors compared with the cohort without MACEs, with the exception of total cholesterol, as shown in Table 1. In the analysis of obesity-related phenotypes stratified by sex, participants who developed MACEs had higher BMI, BFP, and WHR compared to participants without MACEs among men and women, as shown in Table 2.

Based on the comparison of R^2^ values assessing the prediction performance of PPS_BMI_, PPS_BFP_, and PPS_WHR_ across various sample sizes within the training set, we observed a consistent improvement in R^2^ from 10% to 50% of the sample size in the healthy cohort. The performance remained stable for 50% to 90% of the sample size. Since there was no meaningful difference in performance between the 50% and 90% sample sizes among the healthy cohort, we chose to use 50% of the total healthy cohort (N = 7826) as the final training set for fitting LASSO to conduct protein selection for each obesity-related phenotype, as shown in Supplemental Figure S2 and Supplemental Table S1. Then, we randomly divided the healthy cohort into two subsets, a training set and a test set, each comprising 7826 participants.

In the training set of the healthy cohort, LASSO models selected 389, 385, and 176 proteins for prediction of BMI, BFP, and WHR, respectively. The associations between individual proteins and measured obesity-related phenotypes are shown in Supplemental Table S2A–C. Across these LASSO-selected proteins, a total of 213, 226, and 76 distinct proteins were uniquely selected for BMI, BFP, and WHR, respectively, without overlap with the proteins selected for the other traits, as shown in Supplemental Figure S3. Notably, 25 proteins were selected for prediction models across all three obesity traits, as shown in Supplemental Table S2D and Supplemental Figure S3. Pathway enrichment for gene ontology using the proteins selected for BMI and WHR did not reveal significant pathways after FDR correction of multiple testing. The selected proteins for BFP resulted in the cell adhesion pathway with an FDR-corrected q value < 0.05, as shown in Supplemental Table S3A–C.

To maximize the sample size for the prediction of MACEs, the test set of 7826 healthy participants (not used in the construction of PPS) was included in addition to the 24,999 participants without stroke and coronary artery disease at baseline, totaling 32,825 participants used for MACE prediction. Among these participants, the PPS_BMI_, PPS_BFP_, and PPS_WHR_ scores were significantly correlated with the measured phenotypes, with R^2^ of 0.78, 0.85, and 0.63, respectively, as shown in Supplemental Figure S4. The three protein scores were statistically associated with MACEs and the subcomponents of MACEs in Model 1 adjusting for age, sex, and race. The associations remained statistically significant for MACEs and MI in Model 2 additionally adjusting for the measured BMI, BFP, or WHR, as shown in Table 3. In the full Model 3, a standard deviation (SD) increment in PPS_BFP_ and PPS_WHR_ was significantly associated with higher risk for MACEs (HR 1.25, 95% CI 1.14–1.38, *p* < 0.0001; HR 1.15, 95% CI 1.06–1.24, *p* = 0.001, respectively), whereas PPS_BMI_ showed a nominal association (HR 1.08, 95% CI 1.00–1.17, *p* = 0.0524), as shown in Table 3. In Model 3, all three scores remained significantly associated with MI, while PPS_BFP_ remained associated with cardiovascular death, as shown in Table 3 and Supplemental Figure S5. The sensitivity analysis after excluding prevalent cancer resulted in similar findings, as shown in Supplemental Table S4.

A statistically significant interaction of sex with the protein scores was not identified. In the sex-stratified analysis, the associations between PPS_BMI_ and MACEs and MI and the association between PPS_BFP_ and cardiovascular death were significant only among males, while the association between PPS_WHR_ and ischemic stroke was significant only among females. Consistent associations were found among both sex groups between PPS_BFP_ and MACEs and MI, and between PPS_WHR_ and MACEs and MI, as shown in Supplemental Table S5. Sensitivity analyses that excluded individuals with a history of cancer showed similar results, as shown in Supplemental Table S6.

The individual protein scores PPS_BMI_, PPS_BFP_, and PPS_WHR_ had C statistics of 0.557, 0.529, and 0.626 for predicting MACEs, respectively, as shown in Table 4. The combination of three protein scores showed a C statistic of 0.634. Compared with the fully adjusted PREVENT equation model [16] with a C statistic of 0.694, the models adjusted for only age, sex, current smoking, and individual protein scores showed comparable performance (PPS_BMI_ 0.685, PPS_BFP_ 0.684, PPS_WHR_ 0.687). The model adjusted for age, sex, current smoking, and all three protein scores showed a C statistic of 0.688, as shown in Table 4.

## 4. Discussion

This study explored the capacity of proteomic profiles in estimating obesity-related phenotypes and assessed their associations with MACEs. The protein-predicted obesity-related phenotypes—BMI, BFP, and WHR—were strongly correlated with their measured counterparts, suggesting that proteomic profiles capture the complex molecular underpinnings of obesity, potentially providing a more nuanced understanding beyond traditional metrics. Higher protein-predicted scores for BMI, BFP, and WHR were associated with a greater risk of MACEs, even after adjusting for established cardiovascular risk factors. Our findings underscore the potential utility of proteomic data to help characterize the biological impact of adiposity in the prediction of cardiovascular events.

The protein-predicted score of BFP in our study showed a correlation with measured BFP, with an R^2^ of 0.85. A previous study by Williams et al. [18] reported an R^2^ of 0.92 for predicting DEXA-derived BFP in the Fenland cohort using SomaScan (aptamer-based) proteomics data. While both Olink and SomaScan platforms are capable of high-throughput protein quantification, these results may not be directly comparable due to differences in assay technology, protein coverage, phenotyping methods, and modeling approaches. Nonetheless, this result may indicate that certain proteins could play more significant roles in BFP [19,20,21]. The cell adhesion pathway may be enriched with proteins associated with BFP, with several playing crucial roles. CDH2 and CDH5 are central to adherin junction formation [22], while ITGAL, ITGA5, and ITGB6 mediate cell–cell and cell–matrix interactions [23]. Additionally, NCAM1 and SLITRK2 serve as key neuronal adhesion molecules [24]. On the other hand, BMI depends on multiple tissue compositions such as muscle mass, bone density, and fat distribution. Consequently, the protein profile associated with BMI can be more complex, influencing multiple tissue types and physiological processes beyond adiposity alone [25]. Additionally, WHR may also be affected by external factors such as measurement error [26]. The complexities of WHR measurements, including variations in body shape and individual differences in skeletal structure, can introduce additional challenges when correlating proteomic data with this phenotype. The 25 proteins associated with all three obesity phenotypes highlight the multifaceted nature of obesity and emphasize the importance of diverse biological pathways in understanding and addressing this complex condition. For example, GHRL (appetite-regulating hormone) is crucial for regulating appetite and energy balance, thereby influencing body weight and fat distribution [27]. CFH (complement factor H) and AGER (advanced glycosylation end product-specific receptor) may intersect with obesity through their impact on insulin resistance [28,29,30]. BAG3 (BAG family molecular chaperone regulator 3) plays a role in cell survival and responses to stress, making it relevant in various diseases, including metabolic disorders [31]. LEP (leptin) is shared among the proteins selected from LASSO for both BMI and BFP. Leptin resistance, marked by reduced satiety, often leads to obesity [32].

In this population-based study, even with the comprehensive adjustment for risk factors in the PREVENT equation [16], in addition to the measured obesity-related phenotypes, the protein-predicted scores of BFP and WHR consistently demonstrated robust statistical associations with MACEs and MI. This finding underscores the potential utility of proteomic data to enhance risk prediction models for incident cardiovascular disease. Even though the PPS–sex interactions did not reach statistical significance, in the sex-stratified analysis, the associations of PPS_BMI_ with MACEs and MI were only significant in males. A similar pattern was noted for PPS_BFP_ in relation to cardiovascular death. These findings are consistent with earlier studies that have reported sex differences in the impact of obesity on adverse cardiovascular outcomes [33]. Additionally, a sex difference was observed in the association between PPS_WHR_ with ischemic stroke, with significant associations observed only among females. This result is consistent with prior research indicating that women demonstrate a greater excess risk of MI with increased waist circumference and WHR compared to men [34]. Differences in body composition and fat distribution are influenced by sex hormones, and women typically exhibit higher fat mass and subcutaneous fat, while men tend to have more lean mass and visceral fat [34,35]. Such male–female differences highlight the potential of proteomic data in elucidating sex-specific molecular mechanisms underlying cardiovascular disease.

Our study also demonstrates that models incorporating age, sex, smoking status, and obesity-related protein scores can predict MACEs with performance comparable to the PREVENT equation model [16], a widely used tool for cardiovascular risk prediction. The advantage of using protein scores derived from obesity-related phenotypes is that they provide a direct biological assessment of obesity risk, which contributes to several cardiovascular risk factors in the PREVENT model, such as blood pressure, lipids, and diabetes. The ability of protein scores to achieve similar predictive performance to the PREVENT model [16], while requiring only basic demographic and smoking information, has significant practical implications. Age, sex, and smoking status are easily collected in clinical practice, and adding protein scores could offer a more personalized, biologically relevant cardiovascular risk assessment. The integration of simple clinical data with proteomic markers has the potential to improve cardiovascular risk stratification, enhancing its accuracy, cost-effectiveness, and utility in clinical decision-making.

The study of the large biobank cohort with well-characterized phenotypes, comprehensive proteomic data, and long-term follow-up highlights the potential of proteomic profiles in predicting obesity-related phenotypes and their implications for cardiovascular risk prediction. However, several limitations warrant consideration. First, it is important to note that the proteomic profiling in this study was based on measurements of canonical protein products using the Olink platform, and did not distinguish between different proteoforms. This limitation may overlook important biological variability and complexity in the human proteome that could influence both obesity-related phenotypes and cardiovascular risk. Future studies incorporating proteoform-level resolution are warranted to further elucidate these relationships. Second, while the use of the traditional three-point MACE definition allows for a focused and clinically meaningful assessment of high-impact cardiovascular outcomes, it may limit the generalizability of our findings to the broader spectrum of cardiovascular diseases, such as heart failure, stable angina, and peripheral artery disease. Third, our analysis focused on participants from the UK Biobank cohort, a population of predominantly European descent, which may limit the generalizability of our findings to other populations. While proteomic profiling offers valuable insights into molecular pathways associated with obesity and cardiovascular disease, future research should focus on validating these findings in diverse populations and elucidating the molecular mechanisms linking proteomic profiles with adverse cardiovascular outcomes. Fourth, while the PREVENT model serves as a useful benchmark for comparison, it is important to note that differences in cohort characteristics, healthcare systems, and event ascertainment between the US-based derivation cohorts of PREVENT and the UKB may limit the direct applicability of PREVENT in this context. Additionally, an important consideration is how these scores translate into individualized clinical assessments. The PPS is based on relative protein abundance across samples, rather than absolute concentrations, which complicates cross-sample comparisons outside the context of the cohort.

## 5. Conclusions

In summary, we showed that integrating protein-predicted scores of obesity-related traits with readily available clinical variables (e.g., age, sex, smoking status) yields predictive performance for incident MACEs comparable to the established PREVENT equation in the UKB. Further validation in diverse cohorts is needed to confirm the robustness and generalizability of these protein scores. This protein score approach can be complementary to existing MACE prediction methods. However, current proteomic platforms such as Olink and SomaScan, while highly sensitive, are not yet cost-effective or widely accessible for routine clinical use. Our study provides a biologically informed perspective that may inform future risk-stratification strategies in obesity.

## Figures and Tables

**Table 1:** Cohort characteristics among the UK Biobank participants.

Variables	Overall N = 40,651	No MACE N = 36,580 (90.0%)	MACE N = 4071 (10.0%)	Parametric *p* Value No MACE vs. MACE	Non-Parametric *p* Value No MACE vs. MACE
Age (years)	56.44 (8.19)	55.98 (8.20)	60.60 (6.89)	<0.001	<0.001
Male (%)	18,026 (44.3)	15,525 (42.4)	2501 (61.4)	<0.001	<0.001
White (%)	37,997 (93.5)	34,171 (93.4)	3826 (94.0)	0.175	0.170
BMI (kg/m^2^)	27.32 (4.74)	27.19 (4.69)	28.46 (5.04)	<0.001	<0.001
Body Fat Percentage (%)	31.46 (8.57)	31.51 (8.58)	31.00 (8.42)	<0.001	<0.001
Waist–hip Ratio	0.87 (0.09)	0.86 (0.09)	0.91 (0.09)	<0.001	<0.001
Total Cholesterol (mg/dL)	221.97 (43.61)	222.17 (43.22)	220.19 (46.92)	0.007	0.006
HDL-C (mg/dL)	56.48 (14.79)	56.91 (14.75)	52.64 (14.61)	<0.001	<0.001
Systolic Blood Pressure (mmHg)	139.63 (19.64)	138.88 (19.49)	146.35 (19.76)	<0.001	<0.001
eGFR (mL/min/1.73 m^2^)	94.88 (13.07)	95.30 (12.81)	91.13 (14.66)	<0.001	<0.001
Diabetes Prevalence (%)	1845 (4.5)	1419 (3.9)	426 (10.5)	<0.001	<0.001
Current Smoking (%)	4276 (10.6)	3615 (9.9)	661 (16.3)	<0.001	<0.001
Blood-Pressure-Lowering Medication Use (%)	7530 (18.7)	6220 (17.2)	1310 (32.7)	<0.001	<0.001
Cholesterol-Lowering Medication Use (%)	5702 (14.2)	4638 (12.8)	1064 (26.5)	<0.001	<0.001
Ischemic Stroke Incidence (%)	781 (1.9)	0	781 (19.2)	-	-
MI Incidence (%)	3096 (7.6)	0	3096 (76.1)	-	-
CV Death (%)	978 (2.4)	0	978 (24.0)	-	-

Mean (standard deviation) shown unless stated for continuous variables. Count (percentage) shown for categorical variables. BMI: body mass index; HDL-C: high-density lipoprotein cholesterol; eGFR: estimated glomerular filtration rate; MACE: major adverse cardiovascular event (ischemic stroke, myocardial infarction, and cardiovascular death); MI: myocardial infarction; CV: cardiovascular.

**Table 2:** Obesity-related phenotypes stratified by sex among the UK Biobank participants.

Obesity-Related Phenotype	Sex	Overall	No MACE	MACE	Parametric *p* Value No MACE vs. MACE	Non-Parametric *p* Value No MACE vs. MACE
BMI (kg/m^2^)	Male	27.68 (4.14)	27.53 (4.06)	28.58 (4.52)	<0.001	<0.001
	Female	27.03 (5.16)	26.94 (5.10)	28.28 (5.76)	<0.001	<0.001
Body Fat Percentage (%)	Male	25.09 (5.77)	24.85 (5.71)	26.58 (5.87)	<0.001	<0.001
	Female	36.53 (6.88)	36.42 (6.86)	38.04 (6.94)	<0.001	<0.001
Waist–hip Ratio	Male	0.93 (0.06)	0.93 (0.06)	0.95 (0.06)	<0.001	<0.001
	Female	0.82 (0.07)	0.82 (0.07)	0.84 (0.07)	<0.001	<0.001

Mean (standard deviation) shown. BMI: body mass index; MACE: major adverse cardiovascular event (ischemic stroke, myocardial infarction, and cardiovascular death).

**Table 3:** Associations between protein predicted scores and outcomes.

Outcome	Model	PPS_BMI_ (per SD)				PPS_BFP_ (per SD)				PPS_WHR_ (per SD)			
		N	N Event	HR (95% CI)	*p*	N	N Event	HR (95% CI)	*p*	N	N Event	HR (95% CI)	*p*
MACE	Model 1	32,757	4057	1.22 (1.18, 1.26)	<0.0001	32,268	3952	1.29 (1.23, 1.35)	<0.0001	32,816	4070	1.47 (1.4, 1.55)	<0.0001
	Model 2	32,757	4057	1.16 (1.08, 1.24)	<0.0001	32,268	3952	1.35 (1.24, 1.47)	<0.0001	32,816	4070	1.34 (1.26, 1.42)	<0.0001
	Model 3	26,450	3271	1.08 (1, 1.17)	0.0524	26,074	3196	1.25 (1.14, 1.38)	<0.0001	26,495	3282	1.15 (1.06, 1.24)	0.001
Ischemic Stroke	Model 1	32,755	776	1.14 (1.06, 1.23)	0.0005	32,267	767	1.19 (1.07, 1.32)	0.0011	32,814	781	1.32 (1.18, 1.48)	<0.0001
	Model 2	32,755	776	1.11 (0.95, 1.29)	0.1864	32,267	767	1.12 (0.93, 1.35)	0.2162	32,814	781	1.26 (1.1, 1.46)	0.0011
	Model 3	26,450	640	1.11 (0.93, 1.31)	0.2481	26,074	636	1.02 (0.83, 1.25)	0.8681	26,495	643	1.21 (1, 1.46)	0.0523
MI	Model 1	32,757	3093	1.23 (1.19, 1.28)	<0.0001	32,268	3015	1.31 (1.24, 1.38)	<0.0001	32,816	3095	1.52 (1.44, 1.62)	<0.0001
	Model 2	32,757	3093	1.21 (1.12, 1.3)	<0.0001	32,268	3015	1.39 (1.26, 1.53)	<0.0001	32,816	3095	1.41 (1.31, 1.51)	<0.0001
	Model 3	26,450	2496	1.11 (1.02, 1.22)	0.0207	26,074	2438	1.28 (1.14, 1.42)	<0.0001	26,495	2498	1.17 (1.06, 1.28)	0.0011
CV Death	Model 1	32,757	970	1.28 (1.19, 1.38)	<0.0001	32,268	924	1.39 (1.25, 1.54)	<0.0001	32,816	978	1.43 (1.28, 1.59)	<0.0001
	Model 2	32,757	970	1.04 (0.9, 1.2)	0.6069	32,268	924	1.52 (1.27, 1.83)	<0.0001	32,816	978	1.17 (1.02, 1.33)	0.0221
	Model 3	26,450	767	1.02 (0.86, 1.2)	0.8306	26,074	732	1.43 (1.15, 1.76)	0.0011	26,495	774	1.08 (0.91, 1.28)	0.3712

PPS_BMI_: protein-predicted score of BMI; PPS_BFP_: protein-predicted score of body fat percentage; PPS_WHR_: protein-predicted score of waist–hip ratio; MACE: major adverse cardiovascular event (ischemic stroke, myocardial infarction, and cardiovascular death); MI: myocardial infarction; CV: cardiovascular; HR: hazard ratio; CI: confidence interval. Model 1: adjusted for age, sex, race (white vs. other); Model 2: adjusted for the measured obesity-related phenotype (BMI, body fat percentage, or waist–hip ratio), in addition to Model 1; Model 3: adjusted for total cholesterol, high-density lipoprotein cholesterol, systolic blood pressure, estimated glomerular filtration rate calculated using the 2021 CKD-EPI equation, diabetes, current smoking, blood-pressure-lowering medication use, cholesterol-lowering medication use, in addition to Model 2.

**Table 4:** Prediction performance for MACEs.

**Protein-Predicted Score**	**Protein-Predicted Score Model**		PREVENT Model *** **C Statistic**
	Unadjusted Model * **C Statistic**	Protein-Predicted Score Model ** **C Statistic**	
PPS_BMI_	0.557	0.685	0.694
PPS_BFP_	0.529	0.684	
PPS_WHR_	0.626	0.687	
PPS_BMI_ + PPS_BFP_ + PPS_WHR_	0.634	0.688	

PPS_BMI_: protein-predicted score of BMI; PPS_BFP_: protein-predicted score of body fat percentage; PPS_WHR_: protein-predicted score of waist–hip ratio; MACE: major adverse cardiovascular event (ischemic stroke, myocardial infarction, and cardiovascular death); CI: confidence interval. * Model only included protein-predicted score(s). ** Model adjusted for age, sex, current smoking. *** PREVENT model adjusted for age, sex, total cholesterol, high-density lipoprotein cholesterol, systolic blood pressure, BMI, estimated glomerular filtration rate calculated using the 2021 CKD-EPI equation, diabetes, current smoking, blood-pressure-lowering medication use, cholesterol-lowering medication use.

## Data Availability

Restrictions apply to the availability of these data. Data were obtained from The UK Biobank. The UK Biobank will make the data available to all bona fide researchers for all types of health-related research that is in the public interest, without preferential or exclusive access for any persons. All researchers will be subject to the same application process and approval criteria as specified by UK Biobank. For more details on the access procedure, see the UK Biobank website: www.ukbiobank.ac.uk (accessed on 5 October 2025).

## Supplementary Materials

The following supporting information can be downloaded at https://www.mdpi.com/article/10.3390/proteomes13040051/s1; Supplemental Table S1. Performance of the protein-predicted scores by different sample sizes of training set. Supplemental Table S2A. Association between the 389 LASSO-selected proteins and BMI in the training set of the healthy cohort. Supplemental Table S2B. Association between the 385 LASSO-selected proteins and body fat percentage in the training set of the healthy cohort. Supplemental Table S2C. Association between the 176 LASSO-selected proteins and waist–hip ratio in the training set of the healthy cohort. Supplemental Table S2D. The 25 LASSO-selected proteins shared across obesity-related phenotypes. Supplemental Table S3A. Pathway enrichment for gene ontology using the 389 LASSO-selected proteins for BMI. Top 10 pathways out of 86 total pathways with *p* < 0.05 are shown. Supplemental Table S3B. Pathway enrichment for gene ontology using the 385 LASSO-selected proteins for body fat percentage. Top 10 pathways out of 75 total pathways with *p* < 0.05 are shown. Supplemental Table S3C. Pathway enrichment for gene ontology using the 176 LASSO-selected proteins for waist–hip Ratio. Top 10 pathways out of 57 total pathways with *p* < 0.05 are shown. Supplemental Table S4. Associations between protein-predicted scores and outcomes. Sensitivity analysis results after excluding cancer at baseline. Supplemental Table S5. Sex-specific associations between protein-predicted scores and outcomes. Supplemental Table S6. Sex-specific associations between protein-predicted scores and outcomes. Sensitivity analysis results after excluding cancer at baseline. Supplemental Figure S1. Description of the sample selection workflow from the UK Biobank cohort. Supplemental Figure S2. R2 values assessing the prediction performance of protein-predicted scores of BMI (PPS_BMI_), BFP (PPS_BFP_), and WHR (PPS_WHR_) across various sample sizes. The median R2 from the LASSO models with 2.5% and 97.5% percentiles of the 100 iterations are shown. Supplemental Figure S3. The LASSO-selected proteins shared across obesity-related phenotypes. Supplemental Figure S4. Linear associations between predicted protein scores of obesity-related phenotypes and measured phenotypes. Supplemental Figure S5. Forest plot of the associations between protein-predicted scores of obesity-related phenotypes and MACE individual components. Model 1: adjusted for age, sex, and race (white vs. other); Model 2: adjusted for the measured obesity-related phenotype (BMI, body fat percentage, or waist–hip ratio) in addition to Model 1; Model 3: adjusted for total cholesterol, high-density lipoprotein cholesterol, systolic blood pressure, estimated glomerular filtration rate calculated using the 2021 CKD-EPI equation, diabetes, current smoking, blood-pressure-lowering medication use, cholesterol-lowering medication use, in addition to Model 2.

## References

[1] Hales C.M., Carroll M.D., Fryar C.D., Ogden C.L. (2020). Prevalence of Obesity and Severe Obesity Among Adults: United States, 2017–2018.

[2] Powell-Wiley T.M., Poirier P., Burke L.E., Despres J.P., Gordon-Larsen P., Lavie C.J., Lear S.A., Ndumele C.E., Neeland I.J., Sanders P. (2021). Obesity and Cardiovascular Disease: A Scientific Statement from the American Heart Association. Circulation.

[3] Frankenfield D.C., Rowe W.A., Cooney R.N., Smith J.S., Becker D. (2001). Limits of body mass index to detect obesity and predict body composition. Nutrition.

[4] Cheng C.H., Ho C.C., Yang C.F., Huang Y.C., Lai C.H., Liaw Y.P. (2010). Waist-to-hip ratio is a better anthropometric index than body mass index for predicting the risk of type 2 diabetes in Taiwanese population. Nutr. Res..

[5] Goonasegaran A.R., Nabila F.N., Shuhada N.S. (2012). Comparison of the effectiveness of body mass index and body fat percentage in defining body composition. Singap. Med. J..

[6] Zaghlool S.B., Sharma S., Molnar M., Matias-Garcia P.R., Elhadad M.A., Waldenberger M., Peters A., Rathmann W., Graumann J., Gieger C. (2021). Revealing the role of the human blood plasma proteome in obesity using genetic drivers. Nat. Commun..

[7] Helgason H., Eiriksdottir T., Ulfarsson M.O., Choudhary A., Lund S.H., Ivarsdottir E.V., Hjorleifsson Eldjarn G., Einarsson G., Ferkingstad E., Moore K.H.S. (2023). Evaluation of Large-Scale Proteomics for Prediction of Cardiovascular Events. JAMA.

[8] Nurmohamed N.S., Belo Pereira J.P., Hoogeveen R.M., Kroon J., Kraaijenhof J.M., Waissi F., Timmerman N., Bom M.J., Hoefer I.E., Knaapen P. (2022). Targeted proteomics improves cardiovascular risk prediction in secondary prevention. Eur. Heart J..

[9] Sudlow C., Gallacher J., Allen N., Beral V., Burton P., Danesh J., Downey P., Elliott P., Green J., Landray M. (2015). UK biobank: An open access resource for identifying the causes of a wide range of complex diseases of middle and old age. PLoS Med..

[10] Sun B.B., Chiou J., Traylor M., Benner C., Hsu Y.H., Richardson T.G., Surendran P., Mahajan A., Robins C., Vasquez-Grinnell S.G. (2023). Plasma proteomic associations with genetics and health in the UK Biobank. Nature.

[11] Inker L.A., Eneanya N.D., Coresh J., Tighiouart H., Wang D., Sang Y., Crews D.C., Doria A., Estrella M.M., Froissart M. (2021). New Creatinine- and Cystatin C-Based Equations to Estimate GFR without Race. N. Engl. J. Med..

[12] McCaw Z.R., Lane J.M., Saxena R., Redline S., Lin X. (2020). Operating characteristics of the rank-based inverse normal transformation for quantitative trait analysis in genome-wide association studies. Biometrics.

[13] Alexa A., Rahnenfuhrer J., Lengauer T. (2006). Improved scoring of functional groups from gene expression data by decorrelating GO graph structure. Bioinformatics.

[14] Benjamini Y., Hochberg Y. (1995). Controlling the False Discovery Rate: A Practical and Powerful Approach to Multiple Testing. J. R. Stat. Soc. Ser. B (Methodol.).

[15] Fine J.P., Gray R.J. (1999). A Proportional Hazards Model for the Subdistribution of a Competing Risk. J. Am. Stat. Assoc..

[16] Khan S.S., Matsushita K., Sang Y., Ballew S.H., Grams M.E., Surapaneni A., Blaha M.J., Carson A.P., Chang A.R., Ciemins E. (2024). Development and Validation of the American Heart Association’s PREVENT Equations. Circulation.

[17] Uno H., Cai T., Pencina M.J., D’Agostino R.B., Wei L.J. (2011). On the C-statistics for evaluating overall adequacy of risk prediction procedures with censored survival data. Stat. Med..

[18] Williams S.A., Kivimaki M., Langenberg C., Hingorani A.D., Casas J.P., Bouchard C., Jonasson C., Sarzynski M.A., Shipley M.J., Alexander L. (2019). Plasma protein patterns as comprehensive indicators of health. Nat. Med..

[19] Konige M., Wang H., Sztalryd C. (2014). Role of adipose specific lipid droplet proteins in maintaining whole body energy homeostasis. Biochim. Biophys. Acta.

[20] Huemer M.T., Bauer A., Petrera A., Scholz M., Hauck S.M., Drey M., Peters A., Thorand B. (2021). Proteomic profiling of low muscle and high fat mass: A machine learning approach in the KORA S4/FF4 study. J. Cachexia Sarcopenia Muscle.

[21] Liao J., Goodrich J.A., Chen W., Qiu C., Chen J.C., Costello E., Alderete T.L., Chatzi L., Gilliland F., Chen Z. (2024). Cardiometabolic profiles and proteomics associated with obesity phenotypes in a longitudinal cohort of young adults. Sci. Rep..

[22] Kruse K., Lee Q.S., Sun Y., Klomp J., Yang X., Huang F., Sun M.Y., Zhao S., Hong Z., Vogel S.M. (2019). N-cadherin signaling via Trio assembles adherens junctions to restrict endothelial permeability. J. Cell Biol..

[23] Niland S., Eble J.A. (2012). Integrin-mediated cell-matrix interaction in physiological and pathological blood vessel formation. J. Oncol..

[24] Hansen S.M., Berezin V., Bock E. (2008). Signaling mechanisms of neurite outgrowth induced by the cell adhesion molecules NCAM and N-cadherin. Cell Mol. Life Sci..

[25] Goudswaard L.J., Bell J.A., Hughes D.A., Corbin L.J., Walter K., Davey Smith G., Soranzo N., Danesh J., Di Angelantonio E., Ouwehand W.H. (2021). Effects of adiposity on the human plasma proteome: Observational and Mendelian randomisation estimates. Int. J. Obes..

[26] Sebo P., Herrmann F.R., Haller D.M. (2017). Accuracy of anthropometric measurements by general practitioners in overweight and obese patients. BMC Obes..

[27] Abizaid A., Horvath T.L. (2012). Ghrelin and the central regulation of feeding and energy balance. Indian J. Endocrinol. Metab..

[28] Moreno-Navarrete J.M., Martinez-Barricarte R., Catalan V., Sabater M., Gomez-Ambrosi J., Ortega F.J., Ricart W., Bluher M., Fruhbeck G., Rodriguez de Cordoba S. (2010). Complement factor H is expressed in adipose tissue in association with insulin resistance. Diabetes.

[29] Portero-Otin M., de la Maza M.P., Uribarri J. (2023). Dietary Advanced Glycation End Products: Their Role in the Insulin Resistance of Aging. Cells.

[30] Asadipooya K., Uy E.M. (2019). Advanced Glycation End Products (AGEs), Receptor for AGEs, Diabetes, and Bone: Review of the Literature. J. Endocr. Soc..

[31] Rosati A., Graziano V., De Laurenzi V., Pascale M., Turco M.C. (2011). BAG3: A multifaceted protein that regulates major cell pathways. Cell Death Dis..

[32] Obradovic M., Sudar-Milovanovic E., Soskic S., Essack M., Arya S., Stewart A.J., Gojobori T., Isenovic E.R. (2021). Leptin and Obesity: Role and Clinical Implication. Front. Endocrinol..

[33] Di Angelantonio E., Bhupathiraju S.N., Wormser D., Gao P., Kaptoge S., Berrington de Gonzalez A., Cairns B.J., Huxley R., Jackson C.L., The Global BMI Mortality Collaboration (2016). Body-mass index and all-cause mortality: Individual-participant-data meta-analysis of 239 prospective studies in four continents. Lancet.

[34] Peters S.A.E., Bots S.H., Woodward M. (2018). Sex Differences in the Association Between Measures of General and Central Adiposity and the Risk of Myocardial Infarction: Results From the UK Biobank. J. Am. Heart Assoc..

[35] Valencak T.G., Osterrieder A., Schulz T.J. (2017). Sex matters: The effects of biological sex on adipose tissue biology and energy metabolism. Redox Biol..

