# Supplementary material for: Protein-Predicted Obesity Phenotypes and Cardiovascular Events: A Secondary Analysis of UK Biobank Proteomics Data
Source: Proteomes. 2025 Oct 9;13(4):51. doi: 10.3390/proteomes13040051 (PMC12551110; doi:10.3390/proteomes13040051)

Table of Contents

Supplemental Table S1. Performance of the Protein Predicted Scores by Different Sample Sizes of Training set. 3

Supplemental Table S2A. Association between the 389 LASSO Selected Proteins and BMI in the Training Set of the Healthy Cohort. 4

Supplemental Table S2B. Association between the 385 LASSO Selected Proteins and Body Fat Percentage in the Training Set of the Healthy Cohort. 14

Supplemental Table S2C. Association between the 176 LASSO Selected Proteins and Waist-hip Ratio in the Training Set of the Healthy Cohort. 24

Supplemental Table S2D. The 25 LASSO Selected Proteins Shared across Obesity-related Phenotypes. 29

Supplemental Table S3A. Pathway Enrichment for Gene Ontology Using the 389 LASSO-selected Proteins for BMI. Top 10 Pathways out of Total 86 Pathways with $p < 0.05$ are shown. 31

Supplemental Table S3B. Pathway Enrichment for Gene Ontology Using the 385 LASSO-selected Proteins for Body Fat Percentage. Top 10 Pathways out of Total 75 Pathways with $p < 0.05$ are shown. 32

Supplemental Table S3C. Pathway Enrichment for Gene Ontology Using the 176 LASSO-selected Proteins for Waist-hip Ratio. Top 10 Pathways out of Total 57 Pathways with $p < 0.05$ are shown. 33

Supplemental Table S4. Associations Between Protein Predicted Scores and Outcomes. Sensitivity Analysis Results after Excluding Cancer at Baseline. 34

Supplemental Table S5. Sex-Specific Associations Between Protein Predicted Scores and Outcomes. 35

Supplemental Table S6. Sex-Specific Associations Between Protein Predicted Scores and Outcomes. Sensitivity Analysis Results after Excluding Cancer at Baseline. 37

Supplemental Figure S1. Description of the sample selection workflow from the UK Biobank cohort. 39

Supplemental Figure S2. R^2 values assessing the prediction performance of protein-predicted scores of BMI (PPS_{BMI}), BFP (PPS_{BFP}), and WHR (PPS_{WHR}) across various sample sizes. The median R^2 from the LASSO models with 2.5% and 97.5% percentiles of the 100 iterations are shown. 40

Supplemental Figure S3. The LASSO Selected Proteins Shared across Obesity-related Phenotypes. 41

Supplemental Figure S4. Linear Associations Between Predicted Protein Scores of Obesity-related Phenotypes and Measured Phenotypes. 42

Supplemental Figure S5. Forest Plot of the Associations Between Protein Predicted Scores of Obesity-related Phenotypes and MACE Individual Components. Model 1: adjusted for age, sex and race (white vs. other); Model 2: adjusted for the measured obesity-related phenotype (BMI, body fat percentage, or waist-hip ratio) in addition to Model 1; Model 3: adjusted for total cholesterol, high density lipoprotein cholesterol, systolic blood pressure, estimated glomerular filtration rate

calculated using the 2021 CKD-EPI equation, diabetes, current smoking, blood pressure lowering medication use, cholesterol lowering medication use in addition to Model 2. 43

Supplemental Table S1. Performance of the Protein Predicted Scores by Different Sample Sizes of Training set.

Protein Predicted Score	Training Set Sample Size (%)	Median R^2	Percentile 2.5%	Percentile 97.5%
PPS_{BMI}	1564 (10%)	0.7110	0.6969	0.7231
	3127 (20%)	0.7399	0.7309	0.7474
	4690 (30%)	0.7513	0.7433	0.7587
	6254 (40%)	0.7588	0.7503	0.7672
	7817 (50%)	0.7646	0.7558	0.7720
	9380 (60%)	0.7677	0.7593	0.7762
	10944 (70%)	0.7708	0.7610	0.7809
	12507 (80%)	0.7733	0.7618	0.7857
	14070 (90%)	0.7763	0.7568	0.7937
PPS_{BFP}	1548 (10%)	0.8236	0.8148	0.8308
	3096 (20%)	0.8358	0.8310	0.8400
	4644 (30%)	0.8418	0.8378	0.8460
	6191 (40%)	0.8466	0.8408	0.8509
	7739 (50%)	0.8496	0.8442	0.8549
	9287 (60%)	0.8523	0.8471	0.8575
	10834 (70%)	0.8535	0.8465	0.8600
	12382 (80%)	0.8553	0.8465	0.8647
	13930 (90%)	0.8577	0.8411	0.8691
PPS_{WHR}	1566 (10%)	0.5771	0.5624	0.5884
	3131 (20%)	0.5952	0.5868	0.6016
	4696 (30%)	0.6041	0.5961	0.6116
	6261 (40%)	0.6097	0.5992	0.6162
	7826 (50%)	0.6125	0.6034	0.6237
	9391 (60%)	0.6158	0.6035	0.6264
	10956 (70%)	0.6181	0.6017	0.6318
	12521 (80%)	0.6202	0.6027	0.6394
	14086 (90%)	0.6209	0.5906	0.6507

PPS_{BMI}, protein predicted score of BMI; PPS_{BFP}: protein predicted score of body fat percentage; PPS_{WHR}: protein predicted score of waist-hip ratio.

Supplemental Table S2A. Association between the 389 LASSO Selected Proteins and BMI in the Training Set of the Healthy Cohort.

Protein	Name	LASSO	Linear Regression*		
		Beta	Beta (95% CI)	P	FDR
ACAN	Aggrecan core protein	-0.0077	-0.49 (-0.59, -0.4)	1.30E-24	2.68E-24
ACE2	Angiotensin-converting enzyme 2	0.1393	0.86 (0.76, 0.96)	1.20E-65	5.12E-65
ACRV1	Acrosomal protein SP-10	0.0604	0.36 (0.27, 0.46)	3.20E-13	5.51E-13
ADA	Adenosine deaminase	0.0088	0.55 (0.46, 0.64)	2.00E-32	4.74E-32
ADAM12	Disintegrin and metalloproteinase domain-containing protein 12	0.003	0.89 (0.79, 0.98)	6.20E-75	2.75E-74
ADAM15	Disintegrin and metalloproteinase domain-containing protein 15	-0.0319	-0.29 (-0.38, -0.19)	1.90E-09	2.91E-09
ADAMTS15	A disintegrin and metalloproteinase with thrombospondin motifs 15	0.1426	1.67 (1.58, 1.76)	2.10E-259	6.90E-258
ADAMTS16	A disintegrin and metalloproteinase with thrombospondin motifs 16	0.0014	0.21 (0.12, 0.31)	1.10E-05	1.43E-05
ADAMTS8	A disintegrin and metalloproteinase with thrombospondin motifs 8	-0.0136	-0.53 (-0.63, -0.44)	1.50E-29	3.38E-29
ADGRB3	Adhesion G protein-coupled receptor B3	-0.0003	-0.57 (-0.67, -0.48)	7.60E-33	1.86E-32
ADGRG2	Adhesion G-protein coupled receptor G2	-0.1181	-0.97 (-1.06, -0.88)	3.60E-92	2.06E-91
ADM	Pro-adrenomedullin	0.2999	1.79 (1.7, 1.88)	7.00E-289	3.02E-287
AFP	Alpha-fetoprotein	-0.0106	-0.12 (-0.22, -0.03)	0.0088	1.04E-02
AGER	Advanced glycosylation end product-specific receptor	-0.1281	-0.84 (-0.93, -0.74)	1.60E-68	6.92E-68
AGT	Angiotensinogen	-0.0175	-0.19 (-0.28, -0.1)	0.0001	9.74E-05
AKR1C4	Aldo-keto reductase family 1 member C4	0.01	0.32 (0.22, 0.41)	2.70E-11	4.24E-11
AMBP	Protein AMBP	0.0082	1.1 (1.01, 1.2)	5.10E-111	3.49E-110
ANGPTL1	Angiopoietin-related protein 1	-0.0601	-0.44 (-0.53, -0.35)	3.70E-20	6.84E-20
ANGPTL2	Angiopoietin-related protein 2	0.0292	1.12 (1.03, 1.22)	7.10E-120	5.84E-119
ANGPTL7	Angiopoietin-related protein 7	-0.1052	-0.14 (-0.23, -0.04)	0.0052	6.21E-03
ANXA2	Annexin A2	0.0023	0.72 (0.63, 0.81)	1.40E-51	4.55E-51
APCS	Serum amyloid P-component	0.0067	1.78 (1.7, 1.86)	0.00E+00	0.00E+00
APOA1	Apolipoprotein A-I	-0.0865	-0.76 (-0.86, -0.67)	8.30E-60	3.10E-59
APOA4	Apolipoprotein A-IV	-0.0019	-0.23 (-0.32, -0.14)	1.60E-06	2.23E-06
APOF	Apolipoprotein F	-0.0009	-1.4 (-1.49, -1.31)	6.90E-190	1.27E-188
APOL1	Apolipoprotein L1	0.0119	0.6 (0.5, 0.69)	6.10E-36	1.56E-35
APOM	Apolipoprotein M	-0.0177	-0.52 (-0.61, -0.42)	7.70E-27	1.65E-26
AREG	Amphiregulin	-0.0525	-0.22 (-0.32, -0.13)	1.90E-06	2.59E-06
ARG2	Arginase-2, mitochondrial	0.0334	0.09 (0, 0.18)	0.0457	5.23E-02
ARSA	Arylsulfatase A	-0.0121	0.72 (0.63, 0.82)	1.40E-51	4.56E-51
ART3	Ecto-ADP-ribosyltransferase 3	-0.1188	-0.46 (-0.56, -0.37)	2.20E-21	4.32E-21
B4GAT1	Beta-1,4-glucuronyltransferase 1	-0.1476	-0.8 (-0.89, -0.71)	1.50E-64	6.12E-64
BAG3	BAG family molecular chaperone regulator 3	0.0124	1.1 (1, 1.19)	1.40E-115	1.05E-114
BCHE	Cholinesterase	0.0211	1.04 (0.95, 1.14)	1.40E-106	9.05E-106
BCL2	Apoptosis regulator Bcl-2	-0.0023	0.68 (0.59, 0.77)	3.20E-46	9.47E-46
BCL2L11	Bcl-2-like protein 11, Isoform BimL	-0.0144	0 (-0.09, 0.1)	0.9608	9.66E-01
BOC	Brother of CDO	-0.0044	-0.35 (-0.44, -0.26)	3.80E-14	6.71E-14
BPIFB2	BPI fold-containing family B member 2	0.0141	1.52 (1.43, 1.61)	6.40E-226	1.67E-224
BRK1	Protein BRICK1	0.0015	0.42 (0.32, 0.51)	4.50E-18	8.29E-18

BSG	Basigin	0.0491	0.94 (0.85, 1.04)	2.00E-81	9.84E-81
C1QTNF6	Complement C1q tumor necrosis factor-related protein 6	0.012	0.26 (0.17, 0.36)	2.50E-08	3.74E-08
C7	Complement component C7	-0.0478	-0.78 (-0.88, -0.68)	2.30E-55	7.95E-55
CA14	Carbonic anhydrase 14	0.032	-0.92 (-1.02, -0.83)	1.90E-82	9.71E-82
CA4	Carbonic anhydrase 4	0.1754	0.3 (0.2, 0.39)	7.20E-10	1.10E-09
CA6	Carbonic anhydrase 6	0.0288	-0.31 (-0.4, -0.21)	6.90E-11	1.08E-10
CA9	Carbonic anhydrase 9	-0.0419	-1.1 (-1.19, -1)	7.40E-115	5.36E-114
CALB1	Calbindin	-0.0566	-0.7 (-0.8, -0.61)	3.70E-46	1.10E-45
CALB2	Calretinin	0.0639	1.24 (1.15, 1.33)	1.30E-150	1.77E-149
CALCA	Calcitonin	0.0111	0.7 (0.61, 0.79)	5.60E-48	1.72E-47
CALCB	Calcitonin gene-related peptide 2	-0.0155	-0.49 (-0.58, -0.4)	1.70E-24	3.49E-24
CAPS	Calcyphosin	0.0015	0.31 (0.22, 0.4)	4.10E-11	6.42E-11
CCL15	C-C motif chemokine 15	-0.0479	0.22 (0.13, 0.32)	2.20E-06	2.97E-06
CCL19	C-C motif chemokine 19	0.0101	0.69 (0.6, 0.78)	3.50E-50	1.10E-49
CCL23	C-C motif chemokine 23	-0.0072	0.35 (0.26, 0.45)	3.50E-13	5.98E-13
CCL27	C-C motif chemokine 27	-0.1521	0.01 (-0.08, 0.11)	0.776	8.03E-01
CCL28	C-C motif chemokine 28	-0.0381	-0.53 (-0.63, -0.44)	2.50E-29	5.63E-29
CCN5	CCN family member 5	0.2143	1.2 (1.1, 1.3)	7.80E-130	7.61E-129
CD14	Monocyte differentiation antigen CD14	-0.0924	-0.04 (-0.14, 0.06)	0.4207	4.51E-01
CD22	B-cell receptor CD22	0.053	0.97 (0.88, 1.06)	5.80E-89	3.18E-88
CD300LG	CMRF35-like molecule 9	0.1124	-0.01 (-0.11, 0.08)	0.7624	7.93E-01
CD36	Platelet glycoprotein 4	0.0336	0.49 (0.4, 0.59)	5.70E-25	1.18E-24
CD59	CD59 glycoprotein	0.0228	1.18 (1.08, 1.27)	1.10E-116	8.88E-116
CD84	SLAM family member 5	-0.0025	-0.01 (-0.1, 0.08)	0.8093	8.33E-01
CD99	CD99 antigen	0.0552	0.66 (0.56, 0.75)	8.90E-42	2.42E-41
CDA	Cytidine deaminase	0.0039	0.87 (0.78, 0.96)	5.30E-78	2.51E-77
CDCP1	CUB domain-containing protein 1	-0.0589	0.41 (0.31, 0.5)	1.30E-15	2.31E-15
CDH15	Cadherin-15	0.0118	0.56 (0.46, 0.65)	3.60E-29	7.92E-29
CDH6	Cadherin-6	-0.0044	-0.23 (-0.33, -0.14)	2.20E-06	2.99E-06
CDHR1	Cadherin-related family member 1	0.0603	0.68 (0.59, 0.77)	2.30E-46	7.07E-46
CDSN	Corneodesmosin	-0.0102	0.15 (0.05, 0.24)	0.002	2.41E-03
CEACAM5	Carcinoembryonic antigen-related cell adhesion molecule 5	0.0379	-0.12 (-0.22, -0.02)	0.0144	1.68E-02
CELSR2	Cadherin EGF LAG seven-pass G-type receptor 2	-0.0338	0.13 (0.03, 0.22)	0.0088	1.04E-02
CES1	Liver carboxylesterase 1	-0.0395	1.3 (1.2, 1.39)	5.30E-153	7.37E-152
CES2	Cocaine esterase	0.0154	0.71 (0.62, 0.8)	5.30E-51	1.68E-50
CES3	Carboxylesterase 3	0.001	0.7 (0.61, 0.79)	4.40E-50	1.38E-49
CFD	Complement factor D	0.0981	1.38 (1.29, 1.48)	2.30E-177	4.12E-176
CFH	Complement factor H	0.1082	1.84 (1.75, 1.93)	9.9e-324	7.71E-322
CFHR2	Complement factor H-related protein 2	0.0071	0.49 (0.39, 0.58)	6.60E-23	1.32E-22
CFHR4	Complement factor H-related protein 4	0.0024	0.4 (0.31, 0.49)	5.20E-17	9.43E-17
CFHR5	Complement factor H-related protein 5	0.0028	0.78 (0.68, 0.87)	9.10E-58	3.36E-57
CHCHD10	Coiled-coil-helix-coiled-coil-helix domain-containing protein 10, mitochondrial	0.0375	1.05 (0.96, 1.14)	9.90E-104	6.18E-103

CHGB	Secretogranin-1	-0.0551	-0.97 (-1.06, -0.87)	2.00E-87	1.07E-86
CHRDL2	Chordin-like protein 2	-0.0067	0.14 (0.05, 0.23)	0.0028	3.47E-03
CKB	Creatine kinase B-type	-0.1086	-1.79 (-1.87, -1.7)	9.90E-324	7.71E-322
CKMT1A_CKMT 1B	Creatine kinase U-type, mitochondrial	-0.0165	-0.21 (-0.3, -0.12)	1.00E-05	1.40E-05
CLEC3B	Tetranectin	0.0303	0.47 (0.38, 0.56)	2.80E-23	5.56E-23
CLEC5A	C-type lectin domain family 5 member A	-0.0716	-0.34 (-0.44, -0.25)	3.30E-12	5.34E-12
CLEC6A	C-type lectin domain family 6 member A	-0.0193	0.35 (0.25, 0.44)	1.40E-12	2.40E-12
CLMP	CXADR-like membrane protein	0.2649	1.45 (1.36, 1.54)	3.10E-196	6.06E-195
CLSTN2	Calsyntenin-2	-0.0001	-0.44 (-0.54, -0.35)	2.00E-20	3.84E-20
CLSTN3	Calsyntenin-3	0.0041	0.64 (0.55, 0.73)	4.80E-40	1.28E-39
CLUL1	Clusterin-like protein 1	-0.0096	-0.84 (-0.93, -0.74)	2.70E-70	1.19E-69
CNTN3	Contactin-3	0.1141	1.16 (1.07, 1.25)	4.30E-131	4.36E-130
CNTN5	Contactin-5	-0.0442	-0.6 (-0.69, -0.5)	1.20E-35	2.94E-35
COL15A1	Collagen alpha-1(XV) chain	0.3179	1.46 (1.37, 1.55)	8.70E-202	1.79E-200
COL28A1	Collagen alpha-1(XXVIII) chain	0.02	0.25 (0.16, 0.35)	1.70E-07	2.37E-07
COL4A1	Collagen alpha-1(IV) chain	-0.1903	-1.27 (-1.37, -1.18)	1.10E-157	1.63E-156
COL9A1	Collagen alpha-1(IX) chain	0.0013	0.03 (-0.07, 0.12)	0.5889	6.26E-01
COMP	Cartilage oligomeric matrix protein	0.086	0.75 (0.66, 0.85)	2.10E-54	7.28E-54
COMT	Catechol O-methyltransferase	-0.0136	0.29 (0.2, 0.38)	8.60E-10	1.31E-09
CPVL	Probable serine carboxypeptidase CPVL	0.0209	0.24 (0.15, 0.34)	4.60E-07	6.44E-07
CRIM1	Cysteine-rich motor neuron 1 protein	0.1755	0.02 (-0.08, 0.12)	0.6784	7.15E-01
CRLF1	Cytokine receptor-like factor 1	0.0147	0.79 (0.7, 0.88)	7.30E-64	2.91E-63
CRTAC1	Cartilage acidic protein 1	0.0304	0.04 (-0.06, 0.13)	0.4468	4.78E-01
CRYBB2	Beta-crystallin B2	-0.0576	-0.48 (-0.57, -0.38)	2.10E-22	4.07E-22
CSF3R	Granulocyte colony-stimulating factor receptor	0.0027	0.55 (0.46, 0.65)	1.40E-30	3.16E-30
CST1	Cystatin-SN	-0.0121	-0.33 (-0.43, -0.23)	2.50E-11	4.00E-11
CST5	Cystatin-D	-0.089	-0.33 (-0.42, -0.23)	1.10E-11	1.79E-11
CST6	Cystatin-M	-0.0831	-0.17 (-0.26, -0.07)	0.0006	7.39E-04
CSTB	Cystatin-B	0.0409	0.95 (0.86, 1.04)	6.00E-86	3.18E-85
CTBS	Di-N-acetylchitobiase	-0.002	0.68 (0.58, 0.77)	2.10E-44	5.83E-44
CTHRC1	Collagen triple helix repeat-containing protein 1	0.1693	1.2 (1.11, 1.29)	7.00E-134	7.59E-133
CTNNA1	Catenin alpha-1	0.0214	-0.07 (-0.16, 0.03)	0.1585	1.75E-01
CXCL13	C-X-C motif chemokine 13	-0.0335	0.51 (0.42, 0.61)	7.70E-27	1.65E-26
CXCL17	C-X-C motif chemokine 17	-0.2006	-0.15 (-0.25, -0.06)	0.0017	2.13E-03
CXCL9	C-X-C motif chemokine 9	-0.0048	0.06 (-0.04, 0.15)	0.2409	2.62E-01
CYTL1	Cytokine-like protein 1	-0.2013	-0.14 (-0.24, -0.05)	0.0034	4.17E-03
DCLRE1C	Protein artemis	-0.0125	-0.12 (-0.21, -0.02)	0.0136	1.59E-02
DCUN1D1	DCN1-like protein 1	0.0431	-0.07 (-0.17, 0.02)	0.1175	1.32E-01
DEFB103A_DEF B103B	Beta-defensin 103	0.024	0.3 (0.2, 0.39)	4.70E-10	7.29E-10
DEFB4A_DEFB4 B	Beta-defensin 4A	0.0502	0.6 (0.51, 0.7)	1.70E-36	4.37E-36
DKK3	Dickkopf-related protein 3	-0.0759	-0.84 (-0.93, -0.74)	3.70E-69	1.57E-68
DLL1	Delta-like protein 1	0.0581	0.69 (0.59, 0.79)	1.40E-42	3.75E-42

DMP1	Dentin matrix acidic phosphoprotein 1	-0.0295	-0.66 (-0.75, -0.56)	6.20E-42	1.70E-41
DPEP2	Dipeptidase 2	0.0018	0.01 (-0.09, 0.1)	0.9016	9.18E-01
DPT	Dermatopontin	0.0935	1.35 (1.26, 1.44)	1.40E-172	2.34E-171
DSG3	Desmoglein-3	0.0637	0.32 (0.22, 0.41)	4.60E-11	7.26E-11
DSG4	Desmoglein-4	-0.0339	-0.46 (-0.55, -0.37)	4.00E-22	7.77E-22
EDIL3	EGF-like repeat and discoidin I-like domain-containing protein 3	-0.0505	-0.61 (-0.71, -0.52)	2.00E-38	5.14E-38
EDN1	Endothelin-1	0.0334	0.45 (0.35, 0.55)	9.50E-19	1.77E-18
EFEMP1	EGF-containing fibulin-like extracellular matrix protein 1	0.175	0.7 (0.6, 0.8)	5.50E-45	1.60E-44
EGFLAM	Pikachurin	-0.014	-0.27 (-0.36, -0.17)	1.80E-08	2.73E-08
EIF4EBP1	Eukaryotic translation initiation factor 4E-binding protein 1	-0.036	0.39 (0.29, 0.48)	4.70E-16	8.51E-16
ENG	Endoglin	-0.1619	-0.58 (-0.68, -0.49)	1.90E-34	4.72E-34
ENO3	Beta-enolase	0.0344	0.91 (0.81, 1)	1.50E-83	7.75E-83
ENPP6	Glycerophosphocholine cholinephosphodiesterase ENPP6	-0.047	-1.15 (-1.24, -1.07)	1.40E-136	1.61E-135
ENTPD2	Ectonucleoside triphosphate diphosphohydrolase 2	-0.0153	-0.02 (-0.12, 0.07)	0.6209	6.56E-01
EPHA1	Ephrin type-A receptor 1	0.0021	1.17 (1.07, 1.26)	2.40E-126	2.15E-125
ERCC1	DNA excision repair protein ERCC-1	-0.0022	-0.07 (-0.16, 0.03)	0.1561	1.73E-01
ESM1	Endothelial cell-specific molecule 1	-0.0334	-0.93 (-1.02, -0.83)	8.70E-86	4.56E-85
EXTL1	Exostosin-like 1	-0.0501	-0.26 (-0.35, -0.17)	5.40E-08	7.79E-08
EZR	Ezrin	0.0604	0.98 (0.89, 1.07)	8.60E-98	5.17E-97
FABP4	Fatty acid-binding protein, adipocyte	0.34	2.15 (2.07, 2.24)	0.00E+00	0.00E+00
FABP9	Fatty acid-binding protein 9	-0.0259	-0.25 (-0.34, -0.16)	1.80E-07	2.53E-07
FAM13A	Protein FAM13A	-0.0004	0.31 (0.22, 0.4)	5.70E-11	8.92E-11
FAM3D	Protein FAM3D	-0.0405	-0.28 (-0.37, -0.19)	2.70E-09	4.14E-09
FAP	Prolyl endopeptidase FAP	-0.0165	0.53 (0.43, 0.62)	1.10E-27	2.46E-27
FASLG	Tumor necrosis factor ligand superfamily member 6	-0.0084	-0.33 (-0.42, -0.24)	5.90E-12	9.46E-12
FCER2	Low affinity immunoglobulin epsilon Fc receptor	0.0041	0.96 (0.86, 1.05)	1.50E-83	7.64E-83
FCRL6	Fc receptor-like protein 6	-0.0033	-0.06 (-0.15, 0.04)	0.2368	2.59E-01
FDX1	Adrenodoxin, mitochondrial	0.0033	0.44 (0.35, 0.54)	5.20E-21	9.97E-21
FGFBP1	Fibroblast growth factor-binding protein 1	0.0174	-0.14 (-0.23, -0.04)	0.0053	6.35E-03
FGFBP3	Fibroblast growth factor-binding protein 3	0.0206	0.45 (0.35, 0.54)	9.10E-21	1.72E-20
FGL1	Fibrinogen-like protein 1	-0.0523	-0.63 (-0.73, -0.54)	3.00E-40	8.03E-40
FGR	Tyrosine-protein kinase Fgr	-0.0013	0.5 (0.41, 0.59)	9.30E-27	1.96E-26
FLT4	Vascular endothelial growth factor receptor 3	0.0268	0.9 (0.81, 1)	5.30E-79	2.53E-78
FN1	Fibronectin	0.0546	1.1 (1.01, 1.19)	1.10E-122	9.56E-122
FOLR1	Folate receptor alpha	-0.1915	-0.81 (-0.91, -0.72)	1.90E-61	7.49E-61
FSHB	Follitropin subunit beta	-0.0035	-0.38 (-0.48, -0.29)	1.60E-15	2.88E-15
FSTL1	Follistatin-related protein 1	0.0037	0.21 (0.12, 0.31)	1.40E-05	1.88E-05
FUCA1	Tissue alpha-L-fucosidase	-0.0118	0.27 (0.18, 0.37)	2.00E-08	2.97E-08
FURIN	Furin	0.0313	1.74 (1.65, 1.82)	2.80E-298	1.35E-296
FUT3_FUT5	3-galactosyl-N-acetylglucosaminide 4-alpha-L-fucosyltransferase FUT3_4-galactosyl-N-acetylglucosaminide 3-alpha-L-fucosyltransferase FUT5	0.016	0.26 (0.17, 0.36)	1.40E-07	1.95E-07
FZD10	Frizzled-10	-0.0128	-0.07 (-0.16, 0.02)	0.1392	1.56E-01
GABARAP	Gamma-aminobutyric acid receptor-associated protein	-0.0017	0 (-0.09, 0.09)	0.9951	9.97E-01

GAD1	Glutamate decarboxylase 1	-0.0063	-0.18 (-0.27, -0.08)	0.0002	2.17E-04
GAGE2A	G antigen 2A	-0.0055	-0.07 (-0.17, 0.02)	0.1178	1.32E-01
GAL	Galanin peptides	-0.064	-0.59 (-0.68, -0.5)	9.60E-36	2.44E-35
GALNT5	Polypeptide N-acetylgalactosaminyltransferase 5	-0.0011	-0.21 (-0.31, -0.12)	8.50E-06	1.15E-05
GASK1A	Golgi-associated kinase 1A	0.0131	0.88 (0.79, 0.98)	7.40E-77	3.36E-76
GC	Vitamin D-binding protein	-0.0229	-0.09 (-0.18, 0.01)	0.0712	8.07E-02
GDF2	Growth/differentiation factor 2	-0.0688	-0.75 (-0.85, -0.66)	4.50E-57	1.64E-56
GFRAL	GDNF family receptor alpha-like	0.0511	0.39 (0.29, 0.48)	7.70E-16	1.39E-15
GHR	Growth hormone receptor	0.0172	1.61 (1.53, 1.7)	1.00E-263	3.52E-262
GHRL	Appetite-regulating hormone	-0.0668	-1.02 (-1.11, -0.93)	6.90E-101	4.24E-100
GIP	Gastric inhibitory polypeptide	-0.027	-0.12 (-0.22, -0.03)	0.0136	1.59E-02
GOLM2	Protein GOLM2	0.0425	1.09 (1, 1.18)	4.50E-118	3.63E-117
GPD1	Glycerol-3-phosphate dehydrogenase	0.0752	1.67 (1.58, 1.75)	4.70E-284	1.82E-282
GPHA2	Glycoprotein hormone alpha-2	-0.0352	-0.56 (-0.65, -0.47)	3.90E-33	9.75E-33
GPIHBP1	Glycosylphosphatidylinositol-anchored high density lipoprotein-binding protein 1	0.0068	0.18 (0.08, 0.27)	0.0002	2.57E-04
GPR158	Probable G-protein coupled receptor 158	-0.0359	-0.75 (-0.84, -0.66)	1.90E-55	6.71E-55
GRIN2B	Glutamate receptor ionotropic, NMDA 2B	0.0054	0 (-0.1, 0.09)	0.9562	9.64E-01
GRP	Gastrin-releasing peptide	0.0116	-0.1 (-0.19, 0)	0.0428	4.91E-02
GUCA2A	Guanylin	-0.0039	-0.71 (-0.81, -0.62)	3.80E-48	1.18E-47
HDAC8	Histone deacetylase 8	-0.0244	-0.09 (-0.18, 0.01)	0.0681	7.75E-02
HDGF	Hepatoma-derived growth factor	-0.007	0.16 (0.07, 0.26)	0.0005	6.94E-04
HEPACAM2	HEPACAM family member 2	-0.0012	-0.48 (-0.58, -0.39)	4.90E-25	1.01E-24
HGFAC	Hepatocyte growth factor activator	0.0073	0.2 (0.1, 0.29)	0.0001	6.57E-05
HJV	Hemojuvelin	0.0092	1.07 (0.98, 1.16)	4.60E-118	3.66E-117
HRAS	GTPase Hras	0.0237	0.12 (0.03, 0.21)	0.0117	1.38E-02
HRC	Sarcoplasmic reticulum histidine-rich calcium-binding protein	0.0093	-0.36 (-0.45, -0.26)	8.90E-14	1.53E-13
HRG	Histidine-rich glycoprotein	0.0231	0.33 (0.24, 0.43)	2.50E-12	4.02E-12
HS3ST3B1	Heparan sulfate glucosamine 3-O-sulfotransferase 3B1	-0.018	0.01 (-0.08, 0.11)	0.7916	8.17E-01
HS6ST2	Heparan-sulfate 6-O-sulfotransferase 2	-0.0455	-0.27 (-0.37, -0.18)	2.40E-08	3.59E-08
HSPG2	Basement membrane-specific heparan sulfate proteoglycan core protein	0.0257	1.43 (1.33, 1.53)	2.20E-176	3.77E-175
ICAM5	Intercellular adhesion molecule 5	-0.0139	-0.54 (-0.63, -0.45)	2.10E-29	4.57E-29
ICOSLG	ICOS ligand	-0.0945	-0.68 (-0.77, -0.59)	8.40E-47	2.56E-46
ID4	DNA-binding protein inhibitor ID-4	-0.0122	-0.14 (-0.24, -0.05)	0.0019	2.30E-03
IFIT3	Interferon-induced protein with tetratricopeptide repeats 3	-0.0413	0.1 (0, 0.19)	0.039	4.49E-02
IFNL2	Interferon lambda-2	0.0199	0.02 (-0.07, 0.12)	0.6022	6.38E-01
IGFBP1	Insulin-like growth factor-binding protein 1	-0.047	-1.48 (-1.56, -1.39)	1.30E-224	3.18E-223
IGSF3	Immunoglobulin superfamily member 3	0.0366	1.19 (1.1, 1.29)	4.20E-128	3.82E-127
IL17C	Interleukin-17C	0.0653	0.26 (0.16, 0.35)	1.60E-07	2.27E-07
IL1B	Interleukin-1 beta	-0.0231	0.27 (0.17, 0.36)	3.10E-08	4.52E-08
IL1R1	Interleukin-1 receptor type 1	-0.1056	-0.43 (-0.52, -0.33)	1.20E-17	2.21E-17
IL1RN	Interleukin-1 receptor antagonist protein	0.0168	1.79 (1.71, 1.88)	1.1e-319	7.23E-318
IL21R	Interleukin-21 receptor	0.0241	0.04 (-0.05, 0.14)	0.3456	3.71E-01

IL32	Interleukin-32	-0.0189	-0.53 (-0.63, -0.44)	1.10E-27	2.46E-27
IL33	Interleukin-33	-0.0189	-0.03 (-0.12, 0.07)	0.5461	5.82E-01
IL36G	Interleukin-36 gamma	0.0026	0.35 (0.26, 0.45)	1.60E-13	2.67E-13
INSL3	Insulin-like 3	0.0592	0.35 (0.26, 0.44)	3.90E-14	6.76E-14
INSL4	Early placenta insulin-like peptide	0.0358	0.2 (0.11, 0.29)	1.70E-05	2.27E-05
INSL5	Insulin-like peptide INSL5	0.0099	0.56 (0.47, 0.65)	1.80E-32	4.45E-32
ISLR2	Immunoglobulin superfamily containing leucine-rich repeat protein 2	0.0204	-0.14 (-0.23, -0.05)	0.0026	3.24E-03
ITGAV	Integrin alpha-V	-0.007	-0.54 (-0.63, -0.45)	6.50E-30	1.46E-29
ITGB5	Integrin beta-5	-0.0066	-0.13 (-0.23, -0.04)	0.0044	5.27E-03
ITIH4	Inter-alpha-trypsin inhibitor heavy chain H4	0.0242	0.65 (0.56, 0.74)	2.90E-43	8.23E-43
IVD	Isovaleryl-CoA dehydrogenase, mitochondrial	1.70E-05	0.19 (0.1, 0.28)	4.70E-05	6.14E-05
KIAA0319	Dyslexia-associated protein KIAA0319	-0.0104	-0.89 (-0.98, -0.8)	1.60E-77	7.43E-77
KIRREL2	Kin of IRRE-like protein 2	-0.0063	-0.49 (-0.58, -0.39)	8.70E-25	1.78E-24
KLK11	Kallikrein-11	-0.0437	-0.2 (-0.29, -0.1)	0.0001	1.15E-04
KLK12	Kallikrein-12	0.0004	0.01 (-0.08, 0.1)	0.816	8.37E-01
KLK14	Kallikrein-14	-0.0468	-0.41 (-0.5, -0.31)	2.30E-17	4.20E-17
KLK15	Kallikrein-15	-0.0122	-0.13 (-0.22, -0.03)	0.0087	1.04E-02
KLK4	Kallikrein-4	-0.021	-0.34 (-0.43, -0.25)	3.90E-13	6.52E-13
KRT8	Keratin, type II cytoskeletal 8	0.0027	0.23 (0.13, 0.33)	5.90E-06	8.09E-06
L1CAM	Neural cell adhesion molecule L1	0.0508	0.16 (0.07, 0.26)	0.0006	7.96E-04
LACRT	Extracellular glycoprotein lacritin	-0.0479	-0.61 (-0.7, -0.52)	7.60E-39	1.97E-38
LAMB1	Laminin subunit beta-1	0.0166	0 (-0.1, 0.09)	0.9418	9.57E-01
LAMP3	Lysosome-associated membrane glycoprotein 3	0.0639	0.38 (0.28, 0.47)	9.50E-15	1.68E-14
LAYN	Layilin	-0.0479	-0.35 (-0.45, -0.25)	4.50E-12	7.24E-12
LDLR	Low-density lipoprotein receptor	0.0189	1.44 (1.36, 1.53)	2.20E-218	5.08E-217
LEP	Leptin	1.0981	2.3 (2.22, 2.38)	0.00E+00	0.00E+00
LEPR	Leptin receptor	-0.0112	-1.16 (-1.25, -1.07)	1.10E-134	1.28E-133
LGALS3	Galectin-3	0.0303	0.58 (0.48, 0.67)	1.30E-31	3.04E-31
LGALS3BP	Galectin-3-binding protein	0.05	1.15 (1.06, 1.24)	2.80E-128	2.64E-127
LILRA5	Leukocyte immunoglobulin-like receptor subfamily A member 5	0.0333	1.27 (1.17, 1.36)	7.30E-149	9.41E-148
LMNB2	Lamin-B2	0.0243	1.07 (0.98, 1.17)	8.90E-106	5.78E-105
LMOD1	Leiomodin-1	-0.034	-0.07 (-0.17, 0.02)	0.144	1.60E-01
LRPAP1	Alpha-2-macroglobulin receptor-associated protein	0.0341	0.65 (0.56, 0.74)	1.30E-45	3.70E-45
LRRC38	Leucine-rich repeat-containing protein 38	-0.002	0.15 (0.06, 0.24)	0.0016	1.97E-03
LRRN1	Leucine-rich repeat neuronal protein 1	-0.0685	-0.24 (-0.33, -0.15)	4.60E-07	6.44E-07
LRTM2	Leucine-rich repeat and transmembrane domain-containing protein 2	-0.0069	-1.09 (-1.18, -0.99)	2.50E-115	1.82E-114
LTBP2	Latent-transforming growth factor beta-binding protein 2	-0.0565	-0.68 (-0.77, -0.58)	1.70E-44	4.98E-44
LTBP3	Latent-transforming growth factor beta-binding protein 3	0.0182	0.84 (0.74, 0.93)	3.30E-68	1.37E-67
MBL2	Mannose-binding protein C	-0.0223	-0.2 (-0.29, -0.11)	3.20E-05	4.19E-05
MEGF10	Multiple epidermal growth factor-like domains protein 10	0.0028	-0.19 (-0.29, -0.1)	3.00E-05	4.03E-05
MELTF	Melanotransferrin	0.0032	-0.14 (-0.23, -0.04)	0.0037	4.54E-03
MEPE	Matrix extracellular phosphoglycoprotein	-0.058	0.28 (0.18, 0.37)	5.90E-09	8.88E-09
MERTK	Tyrosine-protein kinase Mer	0.036	0.37 (0.28, 0.47)	1.10E-14	2.00E-14

MFGE8	Lactadherin	-0.0389	0.24 (0.15, 0.34)	7.20E-07	1.00E-06
MIA	Melanoma-derived growth regulatory protein	-0.1294	-0.13 (-0.23, -0.04)	0.0059	7.01E-03
MMP12	Macrophage metalloelastase	-0.0652	0 (-0.1, 0.1)	0.9445	9.57E-01
MNDA	Myeloid cell nuclear differentiation antigen	-0.0285	0.33 (0.24, 0.42)	2.30E-12	3.86E-12
MOG	Myelin-oligodendrocyte glycoprotein	-0.1972	-0.8 (-0.89, -0.71)	2.80E-61	1.07E-60
MRC1	Macrophage mannose receptor 1	0.06	1.09 (1, 1.18)	3.40E-112	2.35E-111
MSTN	Growth/differentiation factor 8	0.0272	0.45 (0.36, 0.55)	4.30E-21	8.29E-21
MUC16	Mucin-16	0.0074	0.08 (-0.01, 0.17)	0.0878	9.90E-02
MYBPC2	Myosin-binding protein C, fast-type	0.0041	0.34 (0.24, 0.43)	1.60E-12	2.59E-12
MYL3	Myosin light chain 3	0.0691	0.55 (0.46, 0.65)	3.70E-30	8.38E-30
MYOM3	Myomesin-3	0.0859	0.34 (0.24, 0.43)	3.10E-12	5.12E-12
NADK	NAD kinase	-0.0429	0.71 (0.62, 0.8)	7.80E-52	2.56E-51
NAGPA	N-acetylglucosamine-1-phosphodiester alpha-N-acetylglucosaminidase	0.0078	0.57 (0.48, 0.67)	1.90E-31	4.39E-31
NAP1L4	Nucleosome assembly protein 1-like 4	-0.0256	0.07 (-0.03, 0.16)	0.1607	1.77E-01
NCAM2	Neural cell adhesion molecule 2	-0.0577	-1.06 (-1.15, -0.97)	2.60E-112	1.86E-111
NCAN	Neurocan core protein	-0.1099	-1.09 (-1.19, -1)	1.00E-115	7.86E-115
NEFL	Neurofilament light polypeptide	-0.1485	-0.52 (-0.61, -0.42)	1.50E-24	3.10E-24
NELL1	Protein kinase C-binding protein NELL1	0.063	-0.18 (-0.27, -0.08)	0.0004	4.63E-04
NFATC3	Nuclear factor of activated T-cells, cytoplasmic 3	-0.0367	0.28 (0.19, 0.38)	7.50E-09	1.13E-08
NHLRC3	NHL repeat-containing protein 3	0.0242	1.02 (0.92, 1.12)	1.80E-90	1.03E-89
NPHS2	Podocin	0.0025	0.06 (-0.04, 0.15)	0.2247	2.46E-01
NPPC	C-type natriuretic peptide	-0.0571	0.53 (0.44, 0.63)	4.80E-27	1.04E-26
NPTX1	Neuronal pentraxin-1	-0.0416	-0.56 (-0.65, -0.46)	5.30E-31	1.24E-30
NPTX2	Neuronal pentraxin-2	-0.0024	-0.2 (-0.29, -0.1)	3.40E-05	4.53E-05
NRCAM	Neuronal cell adhesion molecule	0.0104	0.33 (0.23, 0.42)	2.90E-11	4.58E-11
NTRK2	BDNF/NT-3 growth factors receptor	0.1347	0.57 (0.48, 0.66)	2.50E-32	6.04E-32
NTRK3	NT-3 growth factor receptor	-0.0245	-0.99 (-1.08, -0.9)	2.00E-97	1.18E-96
NXPE4	NXPE family member 4	0.0067	0 (-0.09, 0.09)	0.9967	9.97E-01
ODAM	Odontogenic ameloblast-associated protein	-0.0087	-0.33 (-0.42, -0.24)	3.90E-12	6.25E-12
OGT	UDP-N-acetylglucosamine--peptide N-acetylglucosaminyltransferase 110 kDa subunit	-0.0048	-0.09 (-0.18, 0)	0.0573	6.54E-02
OMG	Oligodendrocyte-myelin glycoprotein	0.2207	-0.49 (-0.58, -0.39)	5.20E-24	1.04E-23
OPTC	Opticin	-0.2481	-0.91 (-1, -0.82)	2.00E-81	9.84E-81
PAEP	Glycodelin	-0.0462	-0.14 (-0.22, -0.05)	0.0016	1.94E-03
PALM	Paralemmin-1	0.1206	1.47 (1.38, 1.56)	2.40E-202	5.15E-201
PAM	Peptidyl-glycine alpha-amidating monooxygenase	0.0642	0.46 (0.36, 0.55)	1.80E-20	3.46E-20
PAMR1	Inactive serine protease PAMR1	0.0458	1.32 (1.23, 1.41)	1.30E-170	1.96E-169
PCDH12	Protocadherin-12	-0.0382	-0.01 (-0.1, 0.08)	0.8189	8.38E-01
PCDH17	Protocadherin-17	-0.0321	0.02 (-0.08, 0.11)	0.7218	7.57E-01
PDCD6	Programmed cell death protein 6	-0.0052	-0.05 (-0.14, 0.05)	0.3432	3.70E-01
PENK	Proenkephalin-A	-0.0239	0.15 (0.06, 0.25)	0.0022	2.68E-03
PFDN6	Prefoldin subunit 6	0.0054	0.01 (-0.08, 0.1)	0.8279	8.45E-01
PINLYP	phospholipase A2 inhibitor and Ly6/PLAUR domain-containing protein	-0.0241	0.1 (0.01, 0.19)	0.0373	4.31E-02

PLA2G1B	Phospholipase A2	-0.0326	-0.98 (-1.07, -0.89)	1.20E-90	6.50E-90
PLA2G2A	Phospholipase A2, membrane associated	0.022	0.49 (0.39, 0.58)	4.60E-25	9.58E-25
PM20D1	N-fatty-acyl-amino acid synthase/hydrolase PM20D1	-0.0189	-0.02 (-0.11, 0.08)	0.7378	7.71E-01
PODXL2	Podocalyxin-like protein 2	-0.0154	-0.64 (-0.74, -0.55)	7.00E-40	1.86E-39
POMC	Pro-opiomelanocortin	0.0494	0.56 (0.47, 0.65)	1.50E-31	3.64E-31
PRDX1	Peroxiredoxin-1	-0.0055	-0.06 (-0.15, 0.04)	0.2474	2.69E-01
PRRT3	Proline-rich transmembrane protein 3	-0.2645	-0.91 (-1, -0.81)	5.90E-78	2.75E-77
PRSS2	Trypsin-2	-0.1027	-0.16 (-0.26, -0.07)	0.001	1.24E-03
PRSS27	Serine protease 27	-0.0432	-0.24 (-0.33, -0.14)	6.70E-07	9.37E-07
PRSS53	Serine protease 53	-0.0063	-0.05 (-0.14, 0.05)	0.3269	3.53E-01
PSPN	Persephin	0.1437	0.78 (0.69, 0.88)	9.70E-57	3.51E-56
PTPRR	Receptor-type tyrosine-protein phosphatase R	-0.0494	-0.68 (-0.78, -0.59)	2.70E-45	7.89E-45
PTPRS	Receptor-type tyrosine-protein phosphatase S	0.0105	0.27 (0.17, 0.36)	4.20E-08	6.08E-08
PYDC1	Pyrin domain-containing protein 1	0.0137	0.25 (0.15, 0.34)	2.30E-07	3.31E-07
REG1A	Lithostathine-1-alpha	-0.0299	-0.47 (-0.56, -0.37)	4.80E-22	9.21E-22
REG3A	Regenerating islet-derived protein 3-alpha	-0.0381	-0.38 (-0.47, -0.28)	3.20E-15	5.68E-15
RNASE1	Ribonuclease pancreatic	0.0347	1.23 (1.13, 1.32)	1.00E-130	1.03E-129
ROBO1	Roundabout homolog 1	0.0663	0.48 (0.39, 0.58)	1.90E-22	3.70E-22
ROBO2	Roundabout homolog 2	0.0192	0.02 (-0.08, 0.12)	0.6877	7.23E-01
RPL14	60S ribosomal protein L14	0.0291	0.66 (0.56, 0.75)	7.00E-45	2.00E-44
RSPO1	R-spondin-1	-0.0048	-0.23 (-0.33, -0.14)	1.20E-06	1.64E-06
RSPO3	R-spondin-3	-0.077	0.02 (-0.08, 0.11)	0.7475	7.80E-01
RTN4R	Reticulon-4 receptor	0.0365	1.57 (1.48, 1.66)	1.50E-231	4.45E-230
S100G	Protein S100-G	0.0184	0.01 (-0.08, 0.11)	0.7705	7.99E-01
SCARF2	Scavenger receptor class F member 2	-0.1717	-0.52 (-0.61, -0.42)	8.40E-27	1.79E-26
SCG2	Secretogranin-2	-0.0931	-0.51 (-0.61, -0.42)	2.30E-25	4.72E-25
SCGB1A1	Uteroglobin	-0.01	-0.37 (-0.46, -0.27)	8.20E-14	1.41E-13
SCGB3A1	Secretoglobin family 3A member 1	-0.0528	-0.78 (-0.87, -0.69)	2.70E-60	1.03E-59
SCGB3A2	Secretoglobin family 3A member 2	-0.073	-1.22 (-1.31, -1.13)	1.80E-146	2.27E-145
SCN4B	Sodium channel subunit beta-4	0.004	0.56 (0.47, 0.66)	8.60E-31	2.00E-30
SCPEP1	Retinoid-inducible serine carboxypeptidase	0.0342	0.63 (0.54, 0.73)	8.40E-41	2.28E-40
SELE	E-selectin	0.0638	1.24 (1.14, 1.33)	2.90E-141	3.40E-140
SEPTIN3	Neuronal-specific septin-3	0.0045	0.19 (0.1, 0.29)	0.0001	8.17E-05
SERPINA4	Kallistatin	-0.0471	0.31 (0.21, 0.4)	2.10E-10	3.27E-10
SERPINA7	Thyroxine-binding globulin	0.01	0.59 (0.49, 0.68)	1.30E-34	3.22E-34
SERPINC1	Antithrombin-III	-0.0688	-0.36 (-0.45, -0.27)	4.70E-14	8.22E-14
SETMAR	Histone-lysine N-methyltransferase SETMAR	0.0049	1.29 (1.21, 1.38)	8.70E-170	1.30E-168
SEZ6	Seizure protein 6 homolog	-0.0486	-0.81 (-0.91, -0.72)	1.40E-65	5.81E-65
SEZ6L	Seizure 6-like protein	-0.0715	-1.04 (-1.13, -0.95)	3.80E-105	2.40E-104
SEZ6L2	Seizure 6-like protein 2	0.0306	-0.53 (-0.62, -0.44)	7.10E-30	1.60E-29
SFRP1	Secreted frizzled-related protein 1	0.0856	0.9 (0.8, 0.99)	6.10E-75	2.74E-74
SFRP4	Secreted frizzled-related protein 4	-0.0213	0.72 (0.63, 0.81)	2.90E-54	1.01E-53
SFTPD	Pulmonary surfactant-associated protein D	-0.0953	-0.36 (-0.45, -0.26)	3.00E-13	5.15E-13

SHBG	Sex hormone-binding globulin	-0.0832	-1.45 (-1.53, -1.36)	2.30E-229	6.53E-228
SIGLEC7	Sialic acid-binding Ig-like lectin 7	0.0119	0.76 (0.66, 0.86)	3.90E-53	1.31E-52
SIL1	Nucleotide exchange factor SIL1	0.003	0.46 (0.37, 0.55)	9.40E-23	1.86E-22
SLITRK1	SLIT and NTRK-like protein 1	-0.142	-1.16 (-1.25, -1.07)	7.50E-134	7.92E-133
SLITRK2	SLIT and NTRK-like protein 2	0.0182	0.67 (0.58, 0.77)	2.00E-43	5.69E-43
SMAD5	Mothers against decapentaplegic homolog 5	-0.0039	0.77 (0.68, 0.87)	1.20E-56	4.24E-56
SNCG	Gamma-synuclein	0.0603	1.21 (1.11, 1.3)	1.20E-128	1.17E-127
SORCS2	VPS10 domain-containing receptor SorCS2	-0.0298	0.12 (0.02, 0.21)	0.0233	2.70E-02
SPACA5_SPACA5B	Sperm acrosome-associated protein 5	0.0143	0 (-0.09, 0.1)	0.9522	9.62E-01
SPINK2	Serine protease inhibitor Kazal-type 2	0.0352	0.66 (0.57, 0.75)	1.30E-42	3.66E-42
SPINK6	Serine protease inhibitor Kazal-type 6	0.006	0.79 (0.7, 0.88)	3.90E-62	1.55E-61
SPON2	Spondin-2	-0.0028	0.54 (0.44, 0.64)	9.90E-27	2.08E-26
SPP1	Osteopontin	-0.016	-0.35 (-0.45, -0.26)	5.50E-13	9.22E-13
SSC4D	Scavenger receptor cysteine-rich domain-containing group B protein	0.0317	1.75 (1.66, 1.83)	5.9e-312	3.28E-310
STX1B	Syntaxin-1B	-0.0181	-0.26 (-0.35, -0.17)	5.10E-08	7.39E-08
SUSD2	Sushi domain-containing protein 2	-0.0528	-0.82 (-0.91, -0.73)	1.40E-65	5.81E-65
SUSD4	Sushi domain-containing protein 4	0.0076	-0.15 (-0.25, -0.06)	0.0014	1.73E-03
SYT1	Synaptotagmin-1	-0.0775	-0.63 (-0.72, -0.53)	3.90E-39	1.03E-38
TAGLN3	Transgelin-3	-0.011	-0.08 (-0.18, 0.01)	0.0777	8.79E-02
TARM1	T-cell-interacting, activating receptor on myeloid cells protein 1	-0.0008	-0.13 (-0.22, -0.03)	0.0071	8.45E-03
TCTN3	Tectonic-3	0.0031	0.92 (0.83, 1.02)	2.30E-81	1.13E-80
TFPI	Tissue factor pathway inhibitor	0.0085	0.72 (0.62, 0.81)	4.80E-52	1.57E-51
TGFBR2	TGF-beta receptor type-2	0.2394	1.17 (1.08, 1.27)	3.90E-122	3.30E-121
THOP1	Thimet oligopeptidase	-0.0118	0.85 (0.76, 0.94)	1.60E-70	6.86E-70
THRAP3	Thyroid hormone receptor-associated protein 3	0.0349	0.05 (-0.04, 0.14)	0.2673	2.90E-01
THY1	Thy-1 membrane glycoprotein	0.277	1.1 (1, 1.19)	2.80E-109	1.88E-108
TIMD4	T-cell immunoglobulin and mucin domain-containing protein 4	0.0424	0.74 (0.65, 0.84)	3.50E-53	1.18E-52
TMPRSS11D	Transmembrane protease serine 11D	-0.0891	-0.18 (-0.28, -0.09)	0.0002	2.56E-04
TMPRSS5	Transmembrane protease serine 5	-0.0439	-0.74 (-0.84, -0.65)	2.10E-55	7.34E-55
TNC	Tenascin	-0.0022	-0.12 (-0.22, -0.03)	0.0112	1.32E-02
TNFAIP8	Tumor necrosis factor alpha-induced protein 8	-0.0012	0.07 (-0.02, 0.16)	0.1399	1.56E-01
TNFRSF21	Tumor necrosis factor receptor superfamily member 21	0.0103	0.24 (0.15, 0.34)	8.00E-07	1.12E-06
TNFSF10	Tumor necrosis factor ligand superfamily member 10	0.065	0.78 (0.69, 0.87)	4.50E-61	1.73E-60
TNNI3	Troponin I, cardiac muscle	0.0155	0.14 (0.05, 0.24)	0.0028	3.47E-03
TPK1	Thiamin pyrophosphokinase 1	-0.0071	0.24 (0.14, 0.33)	8.10E-07	1.13E-06
TPSD1	Tryptase delta	0.006	0.1 (0.01, 0.19)	0.0361	4.18E-02
TRAF3	TNF receptor-associated factor 3, Isoform 2	0.0104	0.29 (0.2, 0.38)	5.60E-10	8.62E-10
TREH	Trehalase	-0.0089	0.55 (0.45, 0.64)	2.20E-30	5.14E-30
TRIM25	E3 ubiquitin/ISG15 ligase TRIM25	-0.0222	0.21 (0.12, 0.3)	9.30E-06	1.26E-05
TSC22D1	TSC22 domain family protein 1	0.0105	0.28 (0.19, 0.37)	1.30E-09	2.00E-09
TSHB	Thyrotropin subunit beta	0.0066	0.2 (0.11, 0.3)	1.80E-05	2.40E-05
TYRP1	5,6-dihydroxyindole-2-carboxylic acid oxidase	-0.0143	-0.07 (-0.16, 0.03)	0.1608	1.77E-01

UMOD	Uromodulin	-0.0261	-0.74 (-0.83, -0.65)	1.20E-56	4.38E-56
VASN	Vasorin	0.0226	0.56 (0.47, 0.66)	2.40E-31	5.59E-31
VAT1	Synaptic vesicle membrane protein VAT-1 homolog	0.0211	0.34 (0.25, 0.44)	4.80E-13	8.10E-13
VEGFD	Vascular endothelial growth factor D	-0.0107	-0.99 (-1.08, -0.9)	1.30E-95	7.41E-95
VSIG2	V-set and immunoglobulin domain-containing protein 2	-0.0188	-0.29 (-0.39, -0.19)	1.30E-08	2.00E-08
VWA1	von Willebrand factor A domain-containing protein 1	-0.0115	0.89 (0.8, 0.98)	6.60E-78	3.05E-77
VWC2	Brorin	0.0129	0.59 (0.5, 0.69)	5.20E-33	1.28E-32
VWC2L	von Willebrand factor C domain-containing protein 2-like	-0.1231	-1.03 (-1.13, -0.94)	4.90E-100	2.98E-99
WFDC12	WAP four-disulfide core domain protein 12	0.0288	0.72 (0.63, 0.82)	2.20E-51	6.94E-51
WFIKKN1	WAP, Kazal, immunoglobulin, Kunitz and NTR domain-containing protein 1	0.0608	0.74 (0.64, 0.83)	2.70E-53	9.04E-53
WFIKKN2	WAP, Kazal, immunoglobulin, Kunitz and NTR domain-containing protein 2	-0.0492	-1.22 (-1.31, -1.13)	1.70E-144	2.12E-143
WNT9A	Protein Wnt-9a	-0.028	-0.45 (-0.55, -0.35)	4.20E-19	7.89E-19

BMI, body mass index; CI: confidence interval; FDR: false discovery rate.
*Linear regressions of the measured BMI regressed on each of the proteins (per standard deviation).

Supplemental Table S2B. Association between the 385 LASSO Selected Proteins and Body Fat Percentage in the Training Set of the Healthy Cohort.

Protein	Name	LASSO	Linear Regression*		
		Beta	Beta (95% CI)	P	FDR
ACAN	Aggrecan core protein	-0.0781	-1.53 (-1.71, -1.34)	4.70E-57	1.85E-56
ACHE	Acetylcholinesterase	-0.0112	-2.21 (-2.4, -2.03)	1.90E-118	1.64E-117
ACTN4	Alpha-actinin-4	0.0047	0.16 (-0.03, 0.34)	0.1019	1.16E-01
ADAM12	Disintegrin and metalloproteinase domain-containing protein 12	0.0455	2.19 (2.01, 2.38)	7.60E-115	6.38E-114
ADAMTS15	A disintegrin and metalloproteinase with thrombospondin motifs 15	0.2694	2.71 (2.52, 2.9)	9.40E-168	1.17E-166
ADAMTS16	A disintegrin and metalloproteinase with thrombospondin motifs 16	0.0188	1.87 (1.69, 2.06)	5.10E-85	2.74E-84
ADAMTSL2	ADAMTS-like protein 2	0.0309	1.89 (1.7, 2.08)	1.50E-79	7.60E-79
ADAMTSL4	ADAMTS-like protein 4	-0.052	0.97 (0.79, 1.16)	4.40E-24	1.05E-23
ADGRD1	Adhesion G-protein coupled receptor D1	-0.0535	-1.36 (-1.55, -1.17)	9.90E-45	3.30E-44
ADGRG2	Adhesion G-protein coupled receptor G2	-0.0482	0.69 (0.5, 0.88)	5.40E-13	1.02E-12
ADM	Pro-adrenomedullin	0.3844	3.05 (2.86, 3.24)	1.30E-205	2.21E-204
AGER	Advanced glycosylation end product-specific receptor	-0.0398	-0.24 (-0.43, -0.05)	0.0136	1.68E-02
AGRP	Agouti-related protein	-0.0443	-1.03 (-1.22, -0.85)	1.10E-28	2.74E-28
AGXT	Serine--pyruvate aminotransferase	-0.0232	0.02 (-0.17, 0.21)	0.8375	8.49E-01
AHNAK2	Protein AHNAK2	-0.0242	-0.18 (-0.36, 0.01)	0.0602	6.98E-02
AKR1C4	Aldo-keto reductase family 1 member C4	0.008	0.22 (0.03, 0.4)	0.0238	2.88E-02
AMOT	Angiomotin	0.1023	2.96 (2.79, 3.14)	1.30E-226	2.87E-225
ANGPTL2	Angiopoietin-related protein 2	0.0225	1.2 (1.01, 1.39)	1.00E-34	2.86E-34
ANGPTL7	Angiopoietin-related protein 7	-0.3151	-1.24 (-1.43, -1.05)	1.30E-37	3.83E-37
APLP1	Amyloid-like protein 1	-0.0628	-0.28 (-0.47, -0.09)	0.0039	5.08E-03
APOA4	Apolipoprotein A-IV	-0.2446	-0.87 (-1.06, -0.68)	8.50E-20	1.86E-19
APOL1	Apolipoprotein L1	0.0405	1.37 (1.19, 1.56)	1.90E-47	6.68E-47
ARG2	Arginase-2, mitochondrial	0.0504	0.57 (0.39, 0.75)	6.30E-10	1.07E-09
ARHGAP30	Rho GTPase-activating protein 30	-0.0098	-0.2 (-0.39, -0.02)	0.0292	3.49E-02
ART3	Ecto-ADP-ribosyltransferase 3	-0.178	-3.38 (-3.55, -3.2)	2.00E-289	6.33E-288
ASPN	Asporin	0.0793	1.22 (1.04, 1.4)	4.30E-38	1.31E-37
ATP1B1	Sodium/potassium-transporting ATPase subunit beta-1	-0.0262	-0.39 (-0.57, -0.21)	3.30E-05	4.64E-05
ATP6V1G2	V-type proton ATPase subunit G 2	0.0088	0.06 (-0.12, 0.25)	0.4961	5.30E-01
B4GALT1	Beta-1,4-galactosyltransferase 1	-0.048	-0.08 (-0.28, 0.11)	0.3957	4.30E-01
BAG3	BAG family molecular chaperone regulator 3	-0.0041	0.49 (0.3, 0.68)	5.60E-07	8.56E-07
BCAT2	Branched-chain-amino-acid aminotransferase, mitochondrial	0.0099	0.25 (0.06, 0.43)	0.01	1.25E-02
BGLAP	Osteocalcin	0.0793	1.04 (0.85, 1.23)	7.80E-27	1.96E-26
BLNK	B-cell linker protein	0.018	0.57 (0.39, 0.76)	1.20E-09	1.95E-09
BMP6	Bone morphogenetic protein 6	0.0159	-0.12 (-0.31, 0.07)	0.1999	2.21E-01
BPIFB2	BPI fold-containing family B member 2	0.0643	3.3 (3.13, 3.48)	2.70E-269	7.42E-268
BRME1	Break repair meiotic recombinase recruitment factor 1	0.0073	0.14 (-0.05, 0.32)	0.1405	1.58E-01
C7	Complement component C7	-0.0507	-0.12 (-0.31, 0.08)	0.2393	2.64E-01
CA14	Carbonic anhydrase 14	-0.1317	-3.61 (-3.79, -3.44)	0.00E+00	0.00E+00
CALB2	Calretinin	0.1153	3 (2.82, 3.18)	5.50E-225	1.12E-223

CAPS	Calcyphosin	0.0384	1.03 (0.85, 1.21)	9.40E-29	2.46E-28
CARHSP1	Calcium-regulated heat-stable protein 1	0.0224	-0.6 (-0.79, -0.42)	1.10E-10	1.98E-10
CBLN4	Cerebellin-4	-0.019	0.32 (0.13, 0.51)	0.0009	1.24E-03
CCL15	C-C motif chemokine 15	-0.0306	0.16 (-0.03, 0.34)	0.095	1.09E-01
CCL16	C-C motif chemokine 16	-0.0176	0.01 (-0.18, 0.21)	0.8845	8.94E-01
CCL20	C-C motif chemokine 20	0.0073	1.27 (1.09, 1.45)	1.40E-42	4.44E-42
CCL27	C-C motif chemokine 27	-0.1968	0.08 (-0.11, 0.27)	0.4258	4.60E-01
CCL7	C-C motif chemokine 7	0.065	1.81 (1.63, 1.99)	1.30E-81	6.58E-81
CCNE1	G1/S-specific cyclin-E1	-0.0105	0.08 (-0.1, 0.27)	0.3643	3.97E-01
CD160	CD160 antigen	0.0115	0.75 (0.56, 0.94)	1.40E-14	2.81E-14
CD1C	T-cell surface glycoprotein CD1c	-0.0201	0.08 (-0.11, 0.26)	0.4219	4.58E-01
CD22	B-cell receptor CD22	0.152	2.68 (2.5, 2.86)	2.20E-173	3.37E-172
CD276	CD276 antigen	-0.0617	0.43 (0.23, 0.62)	2.10E-05	3.03E-05
CD300LG	CMRF35-like molecule 9	0.1262	1.46 (1.27, 1.65)	2.30E-51	8.19E-51
CD38	ADP-ribosyl cyclase/cyclic ADP-ribose hydrolase 1	-0.3636	-2.67 (-2.85, -2.49)	1.20E-169	1.58E-168
CD70	CD70 antigen	0.031	1.96 (1.77, 2.14)	1.80E-94	1.12E-93
CD83	CD83 antigen	0.0379	1.81 (1.62, 2)	7.00E-75	3.27E-74
CD86	T-lymphocyte activation antigen CD86	0.0033	0.86 (0.68, 1.05)	1.80E-19	3.89E-19
CD99	CD99 antigen	-0.1061	-2 (-2.19, -1.82)	4.90E-97	3.18E-96
CD99L2	CD99 antigen-like protein 2	0.0451	1.9 (1.72, 2.09)	2.30E-87	1.29E-86
CDH2	Cadherin-2	-0.0524	0.24 (0.04, 0.44)	0.0207	2.52E-02
CDH5	Cadherin-5	-0.0121	-0.42 (-0.61, -0.24)	7.90E-06	1.16E-05
CDHR1	Cadherin-related family member 1	0.0573	0.71 (0.53, 0.9)	7.40E-14	1.42E-13
CDHR5	Cadherin-related family member 5	-0.0041	1.73 (1.55, 1.92)	1.70E-73	8.01E-73
CEACAM21	Carcinoembryonic antigen-related cell adhesion molecule 21	0.0075	0.33 (0.14, 0.51)	0.0005	7.27E-04
CEP164	Centrosomal protein of 164 kDa	0.0147	0.33 (0.14, 0.52)	0.0006	7.60E-04
CFB	Complement factor B	0.1215	2.95 (2.77, 3.12)	2.40E-217	4.68E-216
CFH	Complement factor H	0.0384	2.4 (2.22, 2.59)	2.30E-132	2.32E-131
CFHR2	Complement factor H-related protein 2	0.0188	1.09 (0.9, 1.28)	1.60E-28	4.10E-28
CFI	Complement factor I	0.0412	3.14 (2.96, 3.31)	3.50E-253	8.49E-252
CGB3_CGB5_CGB8	Choriogonadotropin subunit beta 3	0.0754	1.75 (1.56, 1.93)	2.80E-76	1.32E-75
CGREF1	Cell growth regulator with EF hand domain protein 1	-0.0003	0.59 (0.39, 0.78)	2.10E-09	3.38E-09
CHGB	Secretogranin-1	-0.0457	-0.98 (-1.17, -0.79)	2.90E-23	6.87E-23
CHRDL1	Chordin-like protein 1	0.054	2.1 (1.91, 2.29)	1.90E-101	1.36E-100
CHRDL2	Chordin-like protein 2	-0.0585	-0.19 (-0.37, -0.01)	0.0392	4.61E-02
CLC	Galectin-10	-0.0207	0.12 (-0.06, 0.3)	0.1907	2.12E-01
CLEC4A	C-type lectin domain family 4 member A	0.0228	0.03 (-0.15, 0.21)	0.7517	7.82E-01
CLMP	CXADR-like membrane protein	0.9422	4.52 (4.35, 4.68)	0.00E+00	0.00E+00
CLSTN2	Calsyntenin-2	-0.052	-0.75 (-0.94, -0.57)	2.80E-15	5.62E-15
CLU	Clusterin	0.0304	1.22 (1.04, 1.41)	1.40E-38	4.28E-38
CNGB3	Cyclic nucleotide-gated cation channel beta-3	0.005	0.1 (-0.08, 0.28)	0.2809	3.08E-01
CNST	Consortin	-0.0045	0.27 (0.08, 0.45)	0.005	6.34E-03

COL15A1	Collagen alpha-1(XV) chain	0.0778	0.21 (0.02, 0.41)	0.0326	3.86E-02
COL1A1	Collagen alpha-1(I) chain	-0.0419	-0.25 (-0.44, -0.06)	0.0084	1.05E-02
COL3A1	Collagen alpha-1(III) chain	-0.0495	-0.31 (-0.5, -0.13)	0.001	1.36E-03
COL4A1	Collagen alpha-1(IV) chain	-0.2023	-1.46 (-1.65, -1.27)	1.30E-51	4.71E-51
COL4A4	Collagen alpha-4(IV) chain	-0.0193	-0.15 (-0.33, 0.04)	0.1138	1.29E-01
COL6A3	Collagen alpha-3(VI) chain	-0.0423	1.82 (1.62, 2.01)	3.70E-71	1.58E-70
COQ7	5-demethoxyubiquinone hydroxylase, mitochondrial	0.1004	1.05 (0.86, 1.23)	5.70E-29	1.50E-28
CPE	Carboxypeptidase E	-0.0569	-1.07 (-1.25, -0.89)	9.70E-30	2.62E-29
CPOX	Oxygen-dependent coproporphyrinogen-III oxidase, mitochondrial	-0.0024	-0.79 (-0.98, -0.6)	1.30E-16	2.72E-16
CPQ	Carboxypeptidase Q	0.0674	1.12 (0.94, 1.31)	1.60E-31	4.49E-31
CPVL	Probable serine carboxypeptidase CPVL	0.0054	0.67 (0.48, 0.85)	4.70E-12	8.66E-12
CRH	Corticoliberin	-0.0135	-0.9 (-1.09, -0.72)	4.70E-22	1.07E-21
CRIM1	Cysteine-rich motor neuron 1 protein	0.1255	0.56 (0.37, 0.75)	1.10E-08	1.80E-08
CRISP2	Cysteine-rich secretory protein 2	-0.053	-3.23 (-3.4, -3.05)	2.50E-267	6.41E-266
CRYBB2	Beta-crystallin B2	-0.0412	0.14 (-0.05, 0.33)	0.1562	1.75E-01
CSF3R	Granulocyte colony-stimulating factor receptor	0.0548	1.06 (0.88, 1.25)	2.30E-28	5.82E-28
CST5	Cystatin-D	-0.0445	-0.45 (-0.63, -0.26)	3.80E-06	5.70E-06
CST6	Cystatin-M	-0.0753	-1.57 (-1.75, -1.38)	1.90E-60	7.95E-60
CTBS	Di-N-acetylchitobiase	-0.083	-0.05 (-0.25, 0.14)	0.5753	6.10E-01
CTHRC1	Collagen triple helix repeat-containing protein 1	-0.0993	-0.83 (-1.02, -0.64)	4.40E-17	9.14E-17
CTSH	Pro-cathepsin H	0.0532	0.18 (-0.01, 0.36)	0.0662	7.65E-02
CTSO	Cathepsin O	0.0015	0.65 (0.46, 0.84)	4.30E-11	7.67E-11
CTSV	Cathepsin L2	0.0031	-0.41 (-0.6, -0.22)	2.10E-05	2.97E-05
CTSZ	Cathepsin Z	-0.0049	0.41 (0.22, 0.61)	4.00E-05	5.67E-05
CXCL1	Growth-regulated alpha protein	0.0732	0.5 (0.31, 0.68)	1.00E-07	1.64E-07
CXCL11	C-X-C motif chemokine 11	0.0114	1.44 (1.25, 1.62)	1.60E-52	5.70E-52
CXCL16	C-X-C motif chemokine 16	-0.0027	1.8 (1.62, 1.99)	6.60E-78	3.19E-77
CXCL6	C-X-C motif chemokine 6	0.0015	0.57 (0.38, 0.75)	1.80E-09	2.89E-09
CYB5A	Cytochrome b5	-0.0001	-0.03 (-0.22, 0.15)	0.7298	7.64E-01
CYTL1	Cytokine-like protein 1	-0.2192	-1.94 (-2.12, -1.75)	1.20E-89	6.91E-89
DCBLD2	Discoidin, CUB and LCCL domain-containing protein 2	0.0013	0.68 (0.49, 0.88)	5.60E-12	1.02E-11
DCLRE1C	Protein artemis	-0.0462	-0.19 (-0.37, 0)	0.048	5.62E-02
DDC	Aromatic-L-amino-acid decarboxylase	-0.0322	-0.84 (-1.02, -0.65)	1.00E-18	2.18E-18
DDR1	Epithelial discoidin domain-containing receptor 1	-0.0545	-0.87 (-1.06, -0.68)	3.80E-19	8.35E-19
DDT	D-dopachrome decarboxylase	0.0199	0.02 (-0.16, 0.21)	0.7925	8.16E-01
DDX1	ATP-dependent RNA helicase DDX1	0.0197	0.03 (-0.16, 0.21)	0.7876	8.13E-01
DDX53	Probable ATP-dependent RNA helicase DDX53	-0.056	-0.39 (-0.58, -0.2)	3.90E-05	5.49E-05
DEFB104A_DEFB104B	Beta-defensin 104	-0.0028	-1.83 (-2.01, -1.65)	1.50E-85	8.23E-85
DENND2B	DENN domain-containing protein 2B	0.0104	0.15 (-0.04, 0.33)	0.1176	1.33E-01
DIPK2B	Divergent protein kinase domain 2B	0.1487	1.48 (1.3, 1.67)	6.20E-54	2.38E-53
DKK3	Dickkopf-related protein 3	-0.0643	-0.75 (-0.94, -0.57)	3.20E-15	6.48E-15
DMP1	Dentin matrix acidic phosphoprotein 1	0.0545	-0.03 (-0.22, 0.16)	0.7496	7.82E-01

DPEP1	Dipeptidase 1	-0.0294	-1.7 (-1.88, -1.52)	1.60E-72	7.11E-72
DPEP2	Dipeptidase 2	0.01	0.68 (0.49, 0.87)	1.40E-12	2.54E-12
DPP7	Dipeptidyl peptidase 2	-0.0479	-0.25 (-0.44, -0.06)	0.0096	1.19E-02
DPT	Dermatopontin	0.1813	2.08 (1.89, 2.27)	5.50E-102	4.04E-101
DSG2	Desmoglein-2	0.0059	0.26 (0.08, 0.45)	0.006	7.57E-03
DTX3	Probable E3 ubiquitin-protein ligase DTX3	-0.0241	-0.31 (-0.51, -0.12)	0.0015	2.03E-03
ECHDC3	Enoyl-CoA hydratase domain-containing protein 3, mitochondrial	0.0188	0.47 (0.28, 0.65)	9.10E-07	1.38E-06
EFEMP1	EGF-containing fibulin-like extracellular matrix protein 1	0.2057	2.28 (2.09, 2.47)	3.70E-119	3.37E-118
EFNA1	Ephrin-A1	0.0645	1.77 (1.57, 1.96)	3.40E-70	1.47E-69
ELN	Elastin	0.1144	1.96 (1.77, 2.14)	1.20E-90	7.28E-90
ENDOU	Poly(U)-specific endoribonuclease	-0.0106	-2.21 (-2.39, -2.02)	1.50E-114	1.22E-113
ENG	Endoglin	-0.1451	-1.29 (-1.47, -1.1)	6.40E-42	2.08E-41
ENPP2	Ectonucleotide pyrophosphatase/phosphodiesterase family member 2	0.0237	3.63 (3.45, 3.8)	0.00E+00	0.00E+00
ENPP5	Ectonucleotide pyrophosphatase/phosphodiesterase family member 5	-0.0489	-2.76 (-2.93, -2.59)	2.30E-212	4.14E-211
ENPP6	Glycerophosphocholine cholinephosphodiesterase ENPP6	-0.1154	-0.74 (-0.92, -0.55)	5.60E-15	1.11E-14
EPN1	Epsin-1	-0.0013	-0.02 (-0.2, 0.16)	0.8208	8.38E-01
EPO	Erythropoietin	-0.0395	0.62 (0.44, 0.8)	3.70E-11	6.57E-11
EPS8L2	Epidermal growth factor receptor kinase substrate 8-like protein 2	-0.0427	0.27 (0.08, 0.47)	0.0049	6.24E-03
EXTL1	Exostosin-like 1	-0.0131	-0.7 (-0.88, -0.51)	1.70E-13	3.30E-13
F11	Coagulation factor XI	0.0233	1.85 (1.67, 2.04)	7.80E-85	4.15E-84
FABP2	Fatty acid-binding protein, intestinal	-0.1286	-0.47 (-0.66, -0.28)	1.10E-06	1.71E-06
FABP4	Fatty acid-binding protein, adipocyte	1.2469	5.65 (5.5, 5.8)	0.00E+00	0.00E+00
FAM171A2	Protein FAM171A2	-0.0109	-0.01 (-0.19, 0.18)	0.9333	9.36E-01
FAM3B	Protein FAM3B	-0.0434	-0.71 (-0.9, -0.52)	3.00E-13	5.66E-13
FAM3C	Protein FAM3C	-0.0936	-0.24 (-0.44, -0.04)	0.0173	2.13E-02
FCGR3B	Low affinity immunoglobulin gamma Fc region receptor III-B	0.0043	0.91 (0.73, 1.1)	9.20E-23	2.12E-22
FCRLB	Fc receptor-like B	-0.0054	-0.63 (-0.82, -0.44)	1.00E-10	1.83E-10
FGF5	Fibroblast growth factor 5	0.0052	0.27 (0.08, 0.46)	0.0047	5.95E-03
FGFBP3	Fibroblast growth factor-binding protein 3	0.0036	0.95 (0.76, 1.13)	3.70E-23	8.60E-23
FGL1	Fibrinogen-like protein 1	-0.0181	1.53 (1.35, 1.72)	2.20E-58	8.83E-58
FH	Fumarate hydratase, mitochondrial	0.0289	0.31 (0.12, 0.49)	0.0012	1.61E-03
FLRT2	Leucine-rich repeat transmembrane protein FLRT2	-0.0046	0.61 (0.42, 0.8)	3.60E-10	6.13E-10
FLT4	Vascular endothelial growth factor receptor 3	0.0631	1.38 (1.19, 1.56)	3.00E-46	1.04E-45
FNDC1	Fibronectin type III domain-containing protein 1	0.0096	0.99 (0.8, 1.18)	5.00E-24	1.18E-23
FOLR2	Folate receptor beta	0.0336	2.62 (2.44, 2.8)	1.10E-169	1.46E-168
FOLR3	Folate receptor gamma	0.0286	1.43 (1.25, 1.61)	9.00E-54	3.41E-53
FURIN	Furin	0.0208	2.66 (2.47, 2.84)	8.00E-170	1.14E-168
GALNT5	Polypeptide N-acetylgalactosaminyltransferase 5	-0.0098	-0.71 (-0.89, -0.52)	1.40E-13	2.74E-13
GASK1A	Golgi-associated kinase 1A	0.0077	0.9 (0.72, 1.09)	4.20E-21	9.40E-21
GBP6	Guanylate-binding protein 6	0.0258	0.01 (-0.18, 0.19)	0.9298	9.35E-01
GC	Vitamin D-binding protein	-0.0235	0.46 (0.27, 0.64)	1.30E-06	2.01E-06
GH1	Somatotropin	-0.0382	0.86 (0.68, 1.04)	3.60E-21	8.12E-21
GH2	Growth hormone variant	0.001	0.35 (0.16, 0.53)	0.0002	3.41E-04

GHRL	Appetite-regulating hormone	-0.0548	0.34 (0.15, 0.53)	0.0004	5.70E-04
GMPR	GMP reductase 1	-0.0036	-0.97 (-1.15, -0.78)	3.10E-25	7.49E-25
GNLY	Granulysin	0.0074	1.2 (1.02, 1.39)	1.70E-35	4.88E-35
GNPDA2	Glucosamine-6-phosphate isomerase 2	-0.0402	-0.02 (-0.21, 0.17)	0.8247	8.40E-01
GP2	Pancreatic secretory granule membrane major glycoprotein GP2	-0.0028	-1.52 (-1.71, -1.33)	1.00E-55	4.01E-55
GPD1	Glycerol-3-phosphate dehydrogenase	0.0394	2.27 (2.08, 2.45)	2.90E-127	2.80E-126
GRAP2	GRB2-related adapter protein 2	-0.1711	0.28 (0.1, 0.47)	0.003	3.94E-03
GSTA1	Glutathione S-transferase A1	-0.0712	0.83 (0.64, 1.02)	8.20E-18	1.74E-17
GZMA	Granzyme A	0.0769	1.62 (1.44, 1.81)	2.10E-64	8.82E-64
GZMB	Granzyme B	-0.0511	-1.04 (-1.23, -0.86)	4.10E-29	1.09E-28
HDAC8	Histone deacetylase 8	-0.0301	-0.04 (-0.23, 0.14)	0.6507	6.84E-01
HGFAC	Hepatocyte growth factor activator	0.0324	1.21 (1.02, 1.39)	3.00E-36	8.88E-36
HK2	Hexokinase-2	0.004	0.2 (0.02, 0.39)	0.0278	3.33E-02
HMOX1	Heme oxygenase 1	-0.0255	-0.85 (-1.04, -0.66)	1.10E-18	2.32E-18
HRG	Histidine-rich glycoprotein	0.0295	0.98 (0.8, 1.17)	6.10E-25	1.48E-24
HS6ST2	Heparan-sulfate 6-O-sulfotransferase 2	-0.1868	-2.24 (-2.43, -2.05)	5.30E-119	4.76E-118
HSPG2	Basement membrane-specific heparan sulfate proteoglycan core protein	0.0023	1.2 (1, 1.4)	1.50E-31	4.24E-31
ICAM4	Intercellular adhesion molecule 4	0.0213	-0.27 (-0.45, -0.08)	0.0052	6.50E-03
ICOSLG	ICOS ligand	-0.1129	-0.83 (-1.01, -0.64)	2.80E-18	5.97E-18
IDUA	Alpha-L-iduronidase	-0.0005	1.13 (0.94, 1.32)	8.00E-31	2.18E-30
IFNAR1	Interferon alpha/beta receptor 1	0.03	0.63 (0.45, 0.82)	1.40E-11	2.60E-11
IFNL2	Interferon lambda-2	0.0204	0.15 (-0.03, 0.34)	0.0984	1.12E-01
IGDCC4	Immunoglobulin superfamily DCC subclass member 4	-0.013	-1.99 (-2.18, -1.81)	4.00E-98	2.77E-97
IGFBP2	Insulin-like growth factor-binding protein 2	-0.259	-1.43 (-1.62, -1.24)	4.80E-50	1.69E-49
IGFBP6	Insulin-like growth factor-binding protein 6	-0.0061	-1.74 (-1.94, -1.55)	1.20E-67	4.97E-67
IGFBP7	Insulin-like growth factor-binding protein 7	-0.1833	-0.8 (-0.99, -0.6)	9.10E-16	1.85E-15
IL12B	Interleukin-12 subunit beta	0.0172	1.99 (1.8, 2.18)	1.00E-94	6.29E-94
IL17C	Interleukin-17C	0.0415	-0.59 (-0.78, -0.4)	9.30E-10	1.55E-09
IL19	Interleukin-19	-0.0424	-0.42 (-0.61, -0.23)	1.80E-05	2.60E-05
IL1RL1	Interleukin-1 receptor-like 1	-0.0416	-2.01 (-2.19, -1.83)	3.70E-103	2.77E-102
IL1RL2	Interleukin-1 receptor-like 2	0.1683	1.73 (1.54, 1.91)	2.10E-73	9.71E-73
IL2RA	Interleukin-2 receptor subunit alpha	-0.0119	0.52 (0.32, 0.71)	1.80E-07	2.80E-07
IL5RA	Interleukin-5 receptor subunit alpha	0.0108	0.31 (0.12, 0.5)	0.0017	2.21E-03
IL6R	Interleukin-6 receptor subunit alpha	0.0222	0.65 (0.46, 0.84)	1.20E-11	2.14E-11
IMPG1	Interphotoreceptor matrix proteoglycan 1	0.0213	0.21 (0.03, 0.4)	0.0256	3.08E-02
INSL4	Early placenta insulin-like peptide	0.0047	-0.28 (-0.46, -0.1)	0.003	3.91E-03
IPCEF1	Interactor protein for cytohesin exchange factors 1	-0.0314	0.01 (-0.18, 0.19)	0.9277	9.35E-01
ITGA5	Integrin alpha-5	0.099	2.05 (1.86, 2.24)	1.10E-98	7.91E-98
ITGAL	Integrin alpha-L	0.0017	1.24 (1.05, 1.42)	1.20E-37	3.49E-37
ITGB6	Integrin beta-6	-0.1259	-2.77 (-2.95, -2.6)	4.00E-194	6.37E-193
ITIH4	Inter-alpha-trypsin inhibitor heavy chain H4	0.0574	2.98 (2.81, 3.16)	2.80E-231	6.36E-230
JCHAIN	Immunoglobulin J chain	0.024	0.66 (0.47, 0.85)	4.40E-12	8.06E-12

KCNC4	Potassium voltage-gated channel subfamily C member 4	0.0462	0.31 (0.12, 0.49)	0.0012	1.54E-03
KIAA0319	Dyslexia-associated protein KIAA0319	-0.0051	-0.39 (-0.58, -0.2)	0.0001	7.20E-05
KITLG	Kit ligand	-0.0786	-0.55 (-0.74, -0.35)	2.50E-08	3.92E-08
KLK1	Kallikrein-1	-0.0052	-0.39 (-0.58, -0.2)	0.0001	8.98E-05
KLK13	Kallikrein-13	-0.06	-1.15 (-1.33, -0.96)	1.50E-32	4.11E-32
KLK15	Kallikrein-15	-0.0065	-0.04 (-0.23, 0.15)	0.6749	7.08E-01
KLK3	Prostate-specific antigen	0.0671	-4.38 (-4.56, -4.21)	0.00E+00	0.00E+00
KLRD1	Natural killer cells antigen CD94	0.0062	1.2 (1.01, 1.39)	5.50E-36	1.61E-35
KRT5	Keratin, type II cytoskeletal 5	-0.0087	-1.46 (-1.65, -1.28)	2.20E-54	8.33E-54
LAIR1	Leukocyte-associated immunoglobulin-like receptor 1	0.0058	2.1 (1.91, 2.3)	5.40E-98	3.58E-97
LAMB1	Laminin subunit beta-1	0.0336	0.73 (0.54, 0.92)	4.20E-14	8.14E-14
LAMP2	Lysosome-associated membrane glycoprotein 2	0.0633	2.13 (1.94, 2.32)	2.50E-107	1.90E-106
LCN15	Lipocalin-15	-0.0119	-0.71 (-0.9, -0.53)	7.50E-14	1.43E-13
LEFTY2	Left-right determination factor 2	0.123	1.65 (1.47, 1.83)	2.30E-72	1.02E-71
LEP	Leptin	3.0453	7.07 (6.95, 7.18)	0.00E+00	0.00E+00
LEPR	Leptin receptor	-0.0097	-0.59 (-0.78, -0.41)	5.20E-10	8.86E-10
LGALS3	Galectin-3	0.0099	2.04 (1.85, 2.23)	5.10E-98	3.43E-97
LGALS3BP	Galectin-3-binding protein	0.0416	2.42 (2.24, 2.6)	1.50E-143	1.67E-142
LGALS7_LGALS7B	Galectin-7	-0.218	-1.09 (-1.28, -0.89)	1.40E-28	3.66E-28
LGALS9	Galectin-9	0.0972	2.52 (2.33, 2.71)	3.20E-144	3.59E-143
LIFR	Leukemia inhibitory factor receptor	0.0385	0.16 (-0.03, 0.35)	0.1056	1.20E-01
LILRA2	Leukocyte immunoglobulin-like receptor subfamily A member 2	0.0274	2.21 (2.02, 2.39)	4.00E-116	3.42E-115
LILRA3	Leukocyte immunoglobulin-like receptor subfamily A member 3	0.0064	1.18 (0.99, 1.37)	8.50E-35	2.42E-34
LMOD1	Leiomodin-1	-0.0249	-0.7 (-0.89, -0.51)	3.60E-13	6.81E-13
LPCAT2	Lysophosphatidylcholine acyltransferase 2	0.004	0.9 (0.72, 1.09)	1.40E-21	3.15E-21
LRP1	Prolow-density lipoprotein receptor-related protein 1	0.022	0.63 (0.44, 0.81)	1.80E-11	3.27E-11
LRRC38	Leucine-rich repeat-containing protein 38	-0.0719	-1.25 (-1.43, -1.06)	1.50E-39	4.91E-39
LRRN1	Leucine-rich repeat neuronal protein 1	-0.0449	-1.28 (-1.46, -1.09)	1.10E-41	3.66E-41
LXN	Latexin	0.0174	-0.61 (-0.79, -0.42)	1.80E-10	3.09E-10
LYAR	Cell growth-regulating nucleolar protein	-0.0128	0.06 (-0.13, 0.24)	0.5524	5.88E-01
MAN2B2	Epididymis-specific alpha-mannosidase	-0.0004	-0.39 (-0.58, -0.19)	0.0001	1.15E-04
MATN3	Matrilin-3	-0.0314	-1.26 (-1.45, -1.07)	1.80E-38	5.51E-38
MB	Myoglobin	-0.1065	-1.96 (-2.14, -1.78)	9.00E-96	5.80E-95
MBL2	Mannose-binding protein C	-0.0028	-0.81 (-1, -0.62)	2.40E-17	4.98E-17
MCAM	Cell surface glycoprotein MUC18	0.0862	0.11 (-0.07, 0.3)	0.2421	2.66E-01
MELTF	Melanotransferrin	0.027	0.6 (0.41, 0.78)	3.50E-10	5.95E-10
MEPE	Matrix extracellular phosphoglycoprotein	-0.1609	-1.66 (-1.84, -1.48)	4.00E-69	1.68E-68
MFGE8	Lactadherin	-0.0303	-0.54 (-0.73, -0.35)	2.50E-08	4.00E-08
MIA	Melanoma-derived growth regulatory protein	-0.0328	-0.59 (-0.77, -0.4)	1.00E-09	1.70E-09
MLN	Promotilin	0.0727	0.54 (0.35, 0.73)	2.40E-08	3.85E-08
MMP1	Interstitial collagenase	-0.0356	0.4 (0.21, 0.58)	3.90E-05	5.49E-05
MMP3	Stromelysin-1	-0.33	-3.96 (-4.13, -3.79)	0.00E+00	0.00E+00

MMP7	Matrilysin	0.0589	1.66 (1.46, 1.85)	8.70E-59	3.47E-58
MOG	Myelin-oligodendrocyte glycoprotein	-0.1323	-0.24 (-0.43, -0.05)	0.014	1.72E-02
MPO	Myeloperoxidase	0.082	1.01 (0.82, 1.19)	1.50E-26	3.74E-26
MSMB	Beta-microseminoprotein	-0.0777	-1.98 (-2.17, -1.8)	5.50E-92	3.28E-91
MST1	Hepatocyte growth factor-like protein	-0.0161	0.43 (0.23, 0.62)	1.40E-05	2.00E-05
MUC13	Mucin-13	-0.0436	0.21 (0.02, 0.41)	0.0308	3.66E-02
MUCL3	Mucin-like protein 3	0.0158	0.3 (0.12, 0.49)	0.0013	1.76E-03
NAAA	N-acylethanolamine-hydrolyzing acid amidase	-0.0224	-0.55 (-0.74, -0.36)	1.20E-08	1.99E-08
NCAM1	Neural cell adhesion molecule 1	-0.1196	-2.32 (-2.5, -2.14)	1.50E-133	1.58E-132
NCR3LG1	Natural cytotoxicity triggering receptor 3 ligand 1	-0.1467	-1.84 (-2.03, -1.65)	3.50E-81	1.78E-80
NCS1	Neuronal calcium sensor 1	-0.0767	-1.27 (-1.46, -1.08)	4.70E-39	1.49E-38
NECAP2	Adaptin ear-binding coat-associated protein 2	0.0002	0.5 (0.32, 0.69)	1.10E-07	1.79E-07
NECTIN4	Nectin-4	-0.0053	-0.78 (-0.97, -0.58)	1.10E-14	2.24E-14
NEFL	Neurofilament light polypeptide	-0.1199	-0.15 (-0.35, 0.04)	0.1252	1.41E-01
NOS2	Nitric oxide synthase, inducible	0.0312	0.05 (-0.14, 0.23)	0.6084	6.42E-01
NPPC	C-type natriuretic peptide	-0.3373	-2.35 (-2.53, -2.16)	1.10E-129	1.05E-128
NPTX2	Neuronal pentraxin-2	-0.1173	-3.25 (-3.42, -3.07)	3.40E-271	1.00E-269
NPTXR	Neuronal pentraxin receptor	-0.0245	-0.44 (-0.63, -0.25)	5.40E-06	7.89E-06
NRP2	Neuropilin-2	0.001	0.97 (0.78, 1.15)	1.70E-23	4.02E-23
NTF3	Neurotrophin-3	-0.0755	-0.58 (-0.77, -0.4)	1.00E-09	1.74E-09
NTRK2	BDNF/NT-3 growth factors receptor	0.0757	1.25 (1.06, 1.44)	7.20E-39	2.25E-38
OBP2B	Odorant-binding protein 2b	-0.2525	-3.67 (-3.84, -3.5)	0.00E+00	0.00E+00
OLFM4	Olfactomedin-4	-0.03	0.07 (-0.13, 0.26)	0.4971	5.30E-01
OLR1	Oxidized low-density lipoprotein receptor 1	-0.1181	0.27 (0.08, 0.45)	0.0049	6.23E-03
OMG	Oligodendrocyte-myelin glycoprotein	0.0234	-0.21 (-0.4, -0.02)	0.0295	3.52E-02
OPTC	Opticin	-0.0097	0.56 (0.37, 0.75)	5.40E-09	8.76E-09
PADI2	Protein-arginine deiminase type-2	-0.0088	-0.27 (-0.45, -0.08)	0.0045	5.81E-03
PALM	Paralemmin-1	0.0467	2.69 (2.51, 2.88)	9.60E-169	1.24E-167
PBLD	Phenazine biosynthesis-like domain-containing protein	0.0196	0.17 (-0.02, 0.36)	0.0725	8.36E-02
PCDH17	Protocadherin-17	-0.0271	-0.51 (-0.7, -0.32)	1.80E-07	2.72E-07
PDGFRA	Platelet-derived growth factor receptor alpha	-0.1589	-0.41 (-0.6, -0.22)	1.90E-05	2.73E-05
PENK	Proenkephalin-A	-0.0498	0.37 (0.18, 0.57)	0.0002	2.66E-04
PEPD	Xaa-Pro dipeptidase	-0.0164	-0.03 (-0.22, 0.16)	0.7788	8.06E-01
PGA4	Pepsin A-4	-0.0022	-1.45 (-1.65, -1.25)	1.90E-45	6.31E-45
PGF	Placenta growth factor	-0.0063	-0.3 (-0.5, -0.1)	0.0034	4.38E-03
PI3	Elafin	-0.0369	-0.83 (-1.02, -0.64)	8.60E-18	1.81E-17
PLA2G1B	Phospholipase A2	-0.0466	0.02 (-0.17, 0.21)	0.8165	8.36E-01
PLB1	Phospholipase B1, membrane-associated	-0.0195	-1.94 (-2.12, -1.77)	9.50E-101	6.79E-100
PLIN1	Perilipin-1	0.019	2.4 (2.22, 2.58)	6.00E-142	6.41E-141
PLXDC1	Plexin domain-containing protein 1	-0.012	-0.71 (-0.89, -0.52)	4.20E-14	8.14E-14
PODXL	Podocalyxin	0.0049	-0.16 (-0.34, 0.03)	0.0966	1.11E-01
PODXL2	Podocalyxin-like protein 2	-0.0885	-1.04 (-1.23, -0.85)	5.60E-27	1.42E-26
POLR2A	DNA-directed RNA polymerase II subunit RPB1	-0.0094	-0.02 (-0.21, 0.16)	0.8015	8.23E-01

PRCP	Lysosomal Pro-X carboxypeptidase	0.0158	1.02 (0.83, 1.21)	5.10E-25	1.25E-24
PREB	Prolactin regulatory element-binding protein	-0.0484	-0.07 (-0.26, 0.12)	0.4879	5.23E-01
PRRT3	Proline-rich transmembrane protein 3	-0.1134	-0.05 (-0.25, 0.14)	0.5803	6.14E-01
PRSS27	Serine protease 27	-0.0005	-0.23 (-0.41, -0.04)	0.018	2.20E-02
PRSS53	Serine protease 53	-0.0112	-0.42 (-0.6, -0.23)	1.10E-05	1.56E-05
PRTG	Protogenin	-0.0314	-1.07 (-1.26, -0.89)	1.50E-29	4.03E-29
PSCA	Prostate stem cell antigen	0.0107	-0.1 (-0.29, 0.09)	0.3137	3.43E-01
PTGDS	Prostaglandin-H2 D-isomerase	-0.168	-0.86 (-1.05, -0.66)	2.50E-17	5.15E-17
PTH1R	Parathyroid hormone/parathyroid hormone-related peptide receptor	0.0546	0.21 (0.03, 0.4)	0.0245	2.96E-02
PTPRC	Receptor-type tyrosine-protein phosphatase C	0.0614	1.35 (1.17, 1.54)	6.60E-46	2.25E-45
PTPRK	Receptor-type tyrosine-protein phosphatase kappa	0.0632	0.91 (0.73, 1.1)	1.20E-21	2.83E-21
PTS	6-pyruvoyl tetrahydrobiopterin synthase	0.0039	0.64 (0.45, 0.83)	6.70E-11	1.18E-10
PTX3	Pentraxin-related protein PTX3	-0.0928	-0.27 (-0.45, -0.08)	0.0042	5.44E-03
PYDC1	Pyrin domain-containing protein 1	0.0179	-0.66 (-0.85, -0.48)	4.00E-12	7.48E-12
PZP	Pregnancy zone protein	0.1788	4.5 (4.34, 4.65)	0.00E+00	0.00E+00
QPCT	Glutaminyl-peptide cyclotransferase	0.0053	0.64 (0.45, 0.83)	8.50E-11	1.48E-10
RARRES2	Retinoic acid receptor responder protein 2	0.1433	2.93 (2.75, 3.11)	2.10E-208	3.63E-207
RECK	Reversion-inducing cysteine-rich protein with Kazal motifs	0.1162	1.42 (1.24, 1.6)	3.40E-52	1.25E-51
REG1B	Lithostathine-1-beta	-0.0455	-0.46 (-0.64, -0.27)	1.40E-06	2.04E-06
REG3G	Regenerating islet-derived protein 3-gamma	-0.0003	-0.72 (-0.91, -0.53)	4.10E-14	8.00E-14
RELT	Tumor necrosis factor receptor superfamily member 19L	-0.2124	-1.37 (-1.57, -1.18)	8.80E-44	2.93E-43
REN	Renin	-0.1045	-1.45 (-1.67, -1.22)	1.40E-36	4.13E-36
RGMA	Repulsive guidance molecule A	-0.1097	-1.89 (-2.08, -1.71)	1.90E-87	1.05E-86
ROBO1	Roundabout homolog 1	0.013	1.48 (1.29, 1.67)	5.20E-51	1.84E-50
RPL14	60S ribosomal protein L14	0.0024	0.75 (0.57, 0.93)	9.00E-16	1.83E-15
SCG2	Secretogranin-2	-0.0341	-0.52 (-0.72, -0.33)	1.30E-07	2.00E-07
SCGB3A1	Secretoglobin family 3A member 1	-0.0767	0.13 (-0.06, 0.32)	0.176	1.96E-01
SCGB3A2	Secretoglobin family 3A member 2	-0.0432	-0.92 (-1.1, -0.73)	1.50E-21	3.41E-21
SCRG1	Scrapie-responsive protein 1	-0.145	-0.66 (-0.85, -0.46)	3.30E-11	5.96E-11
SERPINA1	Alpha-1-antitrypsin	-0.0145	0.27 (0.08, 0.46)	0.0047	5.95E-03
SERPINA4	Kallistatin	-0.0076	-0.22 (-0.41, -0.03)	0.0218	2.65E-02
SERPIND1	Heparin cofactor 2	0.0386	2.63 (2.44, 2.81)	8.50E-167	1.02E-165
SERPING1	Plasma protease C1 inhibitor	0.0297	1.31 (1.13, 1.5)	2.80E-43	9.07E-43
SEZ6L2	Seizure 6-like protein 2	0.2702	1.91 (1.73, 2.09)	2.00E-94	1.25E-93
SF3B4	Splicing factor 3B subunit 4	0.0659	0.32 (0.14, 0.51)	0.0006	8.41E-04
SFRP1	Secreted frizzled-related protein 1	0.308	2.15 (1.96, 2.34)	2.00E-107	1.59E-106
SFTPD	Pulmonary surfactant-associated protein D	-0.0812	-0.28 (-0.47, -0.09)	0.0046	5.90E-03
SHPK	Sedoheptulokinase	-0.0141	-0.18 (-0.36, 0.01)	0.0577	6.71E-02
SIGLEC10	Sialic acid-binding Ig-like lectin 10	0.0526	1.23 (1.04, 1.43)	2.40E-35	6.99E-35
SIRPA	Tyrosine-protein phosphatase non-receptor type substrate 1	-0.0091	0.5 (0.31, 0.69)	2.40E-07	3.65E-07
SLC9A3R2	Na(+)/H(+) exchange regulatory cofactor NHE-RF2	-0.0003	-0.58 (-0.77, -0.4)	2.70E-10	4.72E-10
SLIT2	Slit homolog 2 protein	0.0032	0.47 (0.28, 0.66)	7.70E-07	1.17E-06
SLITRK2	SLIT and NTRK-like protein 2	0.1278	1.97 (1.78, 2.15)	3.30E-93	1.96E-92

SMOC1	SPARC-related modular calcium-binding protein 1	-0.0394	0.32 (0.13, 0.52)	0.001	1.37E-03
SMOC2	SPARC-related modular calcium-binding protein 2	-0.1115	-0.95 (-1.13, -0.76)	6.70E-23	1.56E-22
SMPD1	Sphingomyelin phosphodiesterase	-0.0642	-0.75 (-0.94, -0.56)	6.60E-15	1.31E-14
SNCG	Gamma-synuclein	0.0614	2.58 (2.39, 2.77)	4.30E-148	4.96E-147
SOD2	Superoxide dismutase [Mn], mitochondrial	-0.1096	-1.48 (-1.66, -1.29)	3.80E-53	1.42E-52
SPESP1	Sperm equatorial segment protein 1	-0.0239	-2.09 (-2.27, -1.9)	6.20E-109	4.95E-108
SPINK6	Serine protease inhibitor Kazal-type 6	-0.1922	-0.54 (-0.73, -0.35)	1.40E-08	2.31E-08
SPON1	Spondin-1	0.0196	1.04 (0.85, 1.23)	5.90E-26	1.46E-25
SPON2	Spondin-2	-0.0285	1.54 (1.34, 1.73)	2.10E-53	7.68E-53
SSC4D	Scavenger receptor cysteine-rich domain-containing group B protein	-0.0092	0.02 (-0.17, 0.21)	0.8352	8.48E-01
ST6GAL1	Beta-galactoside alpha-2,6-sialyltransferase 1	0.0622	1.83 (1.64, 2.01)	2.90E-82	1.52E-81
STX1B	Syntaxin-1B	-0.008	0.03 (-0.16, 0.21)	0.7549	7.83E-01
SUSD5	Sushi domain-containing protein 5	-0.0097	-1.63 (-1.82, -1.44)	2.00E-60	7.95E-60
TBC1D17	TBC1 domain family member 17	0.0115	-0.18 (-0.36, 0.01)	0.0572	6.68E-02
TEX101	Testis-expressed protein 101	-0.0898	-3.73 (-3.9, -3.56)	0.00E+00	0.00E+00
TFF2	Trefoil factor 2	-0.0082	0.52 (0.34, 0.71)	4.10E-08	6.47E-08
TFRC	Transferrin receptor protein 1	0.0251	0.64 (0.46, 0.83)	8.10E-12	1.47E-11
TG	Thyroglobulin	0.065	1.05 (0.87, 1.23)	1.00E-28	2.70E-28
TGFBR2	TGF-beta receptor type-2	0.1827	1.85 (1.65, 2.04)	6.40E-76	2.99E-75
TGM2	Protein-glutamine gamma-glutamyltransferase 2	-0.0259	-0.55 (-0.74, -0.37)	5.40E-09	8.74E-09
THBS2	Thrombospondin-2	-0.0224	0.88 (0.68, 1.07)	5.40E-19	1.17E-18
THBS4	Thrombospondin-4	0.1579	1.86 (1.68, 2.05)	2.00E-83	1.07E-82
THOP1	Thimet oligopeptidase	-0.0952	-0.48 (-0.67, -0.29)	5.60E-07	8.56E-07
THY1	Thy-1 membrane glycoprotein	0.2221	2.72 (2.53, 2.91)	1.50E-170	2.23E-169
TIMP4	Metalloproteinase inhibitor 4	0.151	2.29 (2.1, 2.47)	7.50E-127	7.02E-126
TNC	Tenascin	-0.118	-0.07 (-0.26, 0.12)	0.4549	4.91E-01
TNFRSF19	Tumor necrosis factor receptor superfamily member 19	-0.0508	-0.62 (-0.82, -0.43)	5.10E-10	8.69E-10
TNFRSF6B	Tumor necrosis factor receptor superfamily member 6B	-0.0724	0.46 (0.26, 0.65)	4.90E-06	7.24E-06
TNFSF10	Tumor necrosis factor ligand superfamily member 10	0.0665	0.76 (0.58, 0.95)	9.10E-16	1.85E-15
TNFSF12	Tumor necrosis factor ligand superfamily member 12	0.011	0.35 (0.17, 0.54)	0.0002	2.93E-04
TNNI3	Troponin I, cardiac muscle	0.0012	-0.41 (-0.6, -0.22)	2.00E-05	2.94E-05
TOP1	DNA topoisomerase 1	0.0046	-0.88 (-1.06, -0.69)	1.20E-20	2.57E-20
TPSD1	Tryptase delta	0.0589	0.26 (0.07, 0.44)	0.0067	8.42E-03
TPSG1	Tryptase gamma	0.0129	0.2 (0.01, 0.38)	0.0371	4.37E-02
TREM2	Triggering receptor expressed on myeloid cells 2	0.0341	1.8 (1.61, 1.99)	7.10E-73	3.19E-72
TREML2	Trem-like transcript 2 protein	0.0107	1.26 (1.07, 1.45)	8.20E-39	2.53E-38
TSPAN1	Tetraspanin-1	-0.0162	-0.48 (-0.68, -0.28)	2.30E-06	3.48E-06
TYRP1	5,6-dihydroxyindole-2-carboxylic acid oxidase	-0.0194	0.07 (-0.12, 0.26)	0.4751	5.11E-01
UMOD	Uromodulin	-0.0314	0.48 (0.29, 0.66)	3.60E-07	5.55E-07
UNG	Uracil-DNA glycosylase	-0.0273	0 (-0.19, 0.18)	0.9663	9.66E-01
UXS1	UDP-glucuronic acid decarboxylase 1	-0.0605	-1.12 (-1.3, -0.93)	8.40E-32	2.33E-31
VAMP5	Vesicle-associated membrane protein 5	0.009	0.12 (-0.06, 0.31)	0.1891	2.10E-01
VEGFB	Vascular endothelial growth factor B	0.0535	1.83 (1.64, 2.02)	1.80E-78	8.74E-78

VIT	Vitrin	-0.073	-1.78 (-1.97, -1.6)	1.20E-80	6.04E-80
VSIR	V-type immunoglobulin domain-containing suppressor of T-cell activation	-0.0175	0.2 (0.01, 0.38)	0.0357	4.21E-02
VWA1	von Willebrand factor A domain-containing protein 1	-0.0498	0.97 (0.79, 1.16)	2.60E-24	6.16E-24
VWC2L	von Willebrand factor C domain-containing protein 2-like	-0.0068	0.23 (0.04, 0.42)	0.019	2.32E-02
WFIKKN2	WAP, Kazal, immunoglobulin, Kunitz and NTR domain-containing protein 2	-0.06	-1.18 (-1.37, -0.99)	2.50E-34	7.05E-34
XG	Glycoprotein Xg	0.3044	4.68 (4.52, 4.84)	0.00E+00	0.00E+00

CI: confidence interval; FDR: false discovery.
*Linear regressions of the measured body fat percentage regressed on each of the proteins (per standard deviation).

Supplemental Table S2C. Association between the 176 LASSO Selected Proteins and Waist-hip Ratio in the Training Set of the Healthy Cohort.

Protein	Name	LASSO	Linear Regression*		
		Beta	Beta (95% CI)	P	FDR
ACE2	Angiotensin-converting enzyme 2	0.0012	0.033 (0.031, 0.035)	2.70E-248	2.37E-247
ACRV1	Acrosomal protein SP-10	0.0023	0.041 (0.039, 0.043)	0.00E+00	0.00E+00
ACY1	Aminoacylase-1	0.0007	0.037 (0.035, 0.039)	0.00E+00	0.00E+00
ADGRG2	Adhesion G-protein coupled receptor G2	-0.003	-0.029 (-0.031, -0.027)	9.20E-211	5.04E-210
ADH4	All-trans-retinol dehydrogenase [NAD(+)] ADH4	0.0005	0.033 (0.031, 0.035)	3.00E-274	3.15E-273
ADIPOQ	Adiponectin	-0.0035	-0.043 (-0.044, -0.041)	0.00E+00	0.00E+00
AGER	Advanced glycosylation end product-specific receptor	-0.0004	-0.014 (-0.016, -0.012)	8.90E-50	1.25E-49
ANGPT2	Angiopoietin-2	-0.0003	-0.012 (-0.014, -0.01)	1.60E-34	2.06E-34
ANGPTL3	Angiopoietin-related protein 3	-0.0012	-0.016 (-0.018, -0.014)	2.70E-63	4.16E-63
ANPEP	Aminopeptidase N	-0.0003	0.007 (0.005, 0.009)	2.70E-14	3.07E-14
APCS	ssSerum amyloid P-component	0.0037	0.042 (0.04, 0.043)	0.00E+00	0.00E+00
APOA1	Apolipoprotein A-I	-0.0023	-0.024 (-0.026, -0.022)	1.60E-144	4.52E-144
BAG3	BAG family molecular chaperone regulator 3	0.0001	0.017 (0.015, 0.018)	4.80E-65	7.54E-65
BCAN	Brevican core protein	-8.24E-06	-0.017 (-0.019, -0.015)	4.40E-69	7.47E-69
BCHE	Cholinesterase	0.0003	0.026 (0.024, 0.028)	4.00E-164	1.48E-163
BMP10	Bone morphogenetic protein 10	-0.0004	-0.024 (-0.026, -0.022)	1.70E-149	5.06E-149
C1QL2	Complement C1q-like protein 2	-0.0006	-0.015 (-0.017, -0.013)	1.50E-53	2.19E-53
CCL11	Eotaxin	0.0002	0.009 (0.007, 0.011)	1.10E-22	1.35E-22
CCN5	CCN family member 5	0.0002	0.022 (0.02, 0.024)	8.40E-108	1.84E-107
CD300LG	CMRF35-like molecule 9	-0.0056	-0.023 (-0.025, -0.021)	5.20E-130	1.41E-129
CD59	CD59 glycoprotein	0.001	0.028 (0.026, 0.03)	2.50E-163	9.06E-163
CD99	CD99 antigen	0.0045	0.03 (0.028, 0.031)	1.20E-214	7.03E-214
CDH15	Cadherin-15	0.0004	0.03 (0.028, 0.032)	3.50E-205	1.85E-204
CDHR2	Cadherin-related family member 2	0.0026	0.045 (0.043, 0.046)	0.00E+00	0.00E+00
CDHR5	Cadherin-related family member 5	1.31E-05	0.017 (0.015, 0.019)	3.50E-67	5.70E-67
CEACAM5	Carcinoembryonic antigen-related cell adhesion molecule 5	0.0003	0.005 (0.003, 0.007)	6.00E-08	6.52E-08
CFD	Complement factor D	0.0024	0.02 (0.019, 0.022)	6.70E-95	1.35E-94
CFH	Complement factor H	0.0005	0.026 (0.024, 0.028)	7.40E-154	2.36E-153
CFP	Properdin	0.0005	0.026 (0.024, 0.028)	3.90E-174	1.51E-173
CHGB	Secretogranin-1	-0.001	-0.009 (-0.011, -0.007)	7.70E-21	9.11E-21
CLEC4A	C-type lectin domain family 4 member A	-0.0002	-0.015 (-0.016, -0.013)	2.40E-54	3.53E-54
CLEC5A	C-type lectin domain family 5 member A	-0.001	-0.016 (-0.018, -0.014)	1.10E-58	1.66E-58
CNTN1	Contactin-1	-0.0019	-0.028 (-0.03, -0.026)	5.30E-198	2.47E-197
CNTN3	Contactin-3	0.0019	0.035 (0.033, 0.036)	6.90E-301	8.61E-300
CNTN5	Contactin-5	-0.0006	-0.022 (-0.024, -0.021)	8.40E-123	2.15E-122
CNTNAP2	Contactin-associated protein-like 2	-0.0004	-0.017 (-0.018, -0.015)	2.20E-66	3.51E-66
COL4A1	Collagen alpha-1(IV) chain	-0.002	-0.02 (-0.022, -0.019)	3.30E-100	7.06E-100
COMP	Cartilage oligomeric matrix protein	0.0016	0.015 (0.013, 0.017)	2.70E-51	3.82E-51
CPA1	Carboxypeptidase A1	9.38E-08	0.006 (0.004, 0.008)	6.90E-09	7.49E-09

CPM	Carboxypeptidase M	0.0002	0.032 (0.03, 0.034)	1.50E-241	1.08E-240
CRISP2	Cysteine-rich secretory protein 2	0.0016	0.025 (0.023, 0.027)	5.90E-157	1.92E-156
CTBS	Di-N-acetylchitobiase	0.0007	0.021 (0.02, 0.023)	1.80E-109	3.97E-109
CTHRC1	Collagen triple helix repeat-containing protein 1	2.11E-06	0.028 (0.026, 0.03)	6.80E-182	2.93E-181
CTSF	Cathepsin F	-0.0003	-0.01 (-0.012, -0.009)	8.10E-28	9.84E-28
CTSL	Cathepsin L1	0.0003	0.014 (0.012, 0.016)	2.70E-48	3.77E-48
CTSV	Cathepsin L2	-0.0004	-0.008 (-0.01, -0.006)	1.60E-15	1.90E-15
CXCL17	C-X-C motif chemokine 17	0.0006	0.008 (0.006, 0.01)	1.10E-15	1.33E-15
DCC	Netrin receptor DCC	-1.78E-05	-0.006 (-0.008, -0.005)	1.20E-11	1.32E-11
DEFB4A_DEFB4B	Beta-defensin 4A	1.71E-06	0.025 (0.024, 0.027)	6.90E-160	2.39E-159
DIPK2B	Divergent protein kinase domain 2B	-0.0011	-0.018 (-0.02, -0.016)	2.30E-81	4.25E-81
DLK1	Protein delta homolog 1	0.0006	0.013 (0.011, 0.015)	6.80E-40	9.04E-40
DMP1	Dentin matrix acidic phosphoprotein 1	-0.0004	-0.017 (-0.019, -0.015)	2.80E-67	4.60E-67
DPT	Dermatopontin	0.0043	0.016 (0.014, 0.018)	2.30E-61	3.45E-61
DSG2	Desmoglein-2	-0.0018	-0.029 (-0.031, -0.027)	1.60E-204	8.34E-204
DSG4	Desmoglein-4	-0.0009	-0.007 (-0.009, -0.005)	2.50E-14	2.86E-14
DTX2	Probable E3 ubiquitin-protein ligase DTX2	-0.0005	-0.006 (-0.008, -0.005)	8.80E-12	9.75E-12
EGFLAM	Pikachurin	-0.0001	-0.009 (-0.011, -0.008)	2.70E-23	3.19E-23
ENPP2	Ectonucleotide pyrophosphatase/phosphodiesterase family member 2	-0.0015	-0.034 (-0.035, -0.032)	1.80E-276	2.02E-275
ENPP6	Glycerophosphocholine cholinephosphodiesterase ENPP6	-0.0002	-0.019 (-0.021, -0.018)	4.40E-95	9.08E-95
EPHA1	Ephrin type-A receptor 1	0.0004	0.032 (0.03, 0.034)	1.30E-245	1.07E-244
ERBB2	Receptor tyrosine-protein kinase erbB-2	0.0031	0.033 (0.032, 0.035)	8.00E-280	9.44E-279
ERI1	3'-5' exoribonuclease 1	6.89E-06	0.013 (0.011, 0.015)	9.20E-44	1.25E-43
FASLG	Tumor necrosis factor ligand superfamily member 6	-0.0009	-0.017 (-0.019, -0.015)	3.40E-68	5.63E-68
FBN2	Fibrillin-2	-0.0004	-0.004 (-0.006, -0.002)	0.0001	1.16E-04
FCGR3B	Low affinity immunoglobulin gamma Fc region receptor III-B	-0.0005	-0.008 (-0.01, -0.006)	1.00E-18	1.17E-18
FGF19	Fibroblast growth factor 19	-0.0003	-0.007 (-0.009, -0.005)	3.00E-12	3.36E-12
FGF21	Fibroblast growth factor 21	0.0014	0.022 (0.021, 0.024)	6.10E-123	1.58E-122
FGL1	Fibrinogen-like protein 1	-0.0006	-0.025 (-0.027, -0.024)	6.90E-159	2.35E-158
FN1	Fibronectin	0.0003	0.022 (0.02, 0.023)	1.00E-117	2.55E-117
FOLR1	Folate receptor alpha	-0.0013	-0.02 (-0.022, -0.018)	5.10E-90	9.72E-90
FSHB	Follitropin subunit beta	-0.0014	-0.029 (-0.031, -0.028)	8.40E-216	5.26E-215
GAGE2A	G antigen 2A	-0.0001	-0.001 (-0.003, 0)	0.1327	1.34E-01
GDF15	Growth/differentiation factor 15	0.0013	0.013 (0.011, 0.015)	7.70E-36	9.76E-36
GH1	Somatotropin	-0.0007	-0.03 (-0.031, -0.028)	4.70E-244	3.60E-243
GHR	Growth hormone receptor	0.0001	0.021 (0.019, 0.023)	1.70E-104	3.64E-104
GHRL	Appetite-regulating hormone	-0.0008	-0.022 (-0.023, -0.02)	1.50E-112	3.43E-112
GLA	Alpha-galactosidase A	0.0003	0.027 (0.025, 0.029)	7.50E-178	3.07E-177
GNGT1	Guanine nucleotide-binding protein G(T) subunit gamma-T1	-0.0002	-0.005 (-0.007, -0.003)	2.10E-08	2.33E-08
GPD1	Glycerol-3-phosphate dehydrogenase	0.0004	0.022 (0.02, 0.024)	8.40E-119	2.11E-118
GPR158	Probable G-protein coupled receptor 158	-0.0004	-0.013 (-0.015, -0.011)	9.90E-41	1.32E-40
GPR15L	Protein GPR15L	0.0009	0.02 (0.018, 0.021)	1.00E-94	2.03E-94

GUSB	Beta-glucuronidase	0.0011	0.038 (0.036, 0.04)	0.00E+00	0.00E+00
HGF	Hepatocyte growth factor	0.0005	0.026 (0.025, 0.028)	3.30E-162	1.17E-161
HLA-DRA	HLA class II histocompatibility antigen, DR alpha chain	-0.0003	-0.002 (-0.003, 0)	0.0965	9.93E-02
HSPB6	Heat shock protein beta-6	0.0038	0.03 (0.028, 0.031)	8.10E-211	4.58E-210
HTR1B	5-hydroxytryptamine receptor 1B	0.0004	0 (-0.002, 0.002)	0.8244	8.24E-01
IGDCC4	Immunoglobulin superfamily DCC subclass member 4	-0.0008	-0.001 (-0.002, 0.001)	0.5862	5.90E-01
IGFBP1	Insulin-like growth factor-binding protein 1	-0.0025	-0.03 (-0.032, -0.029)	2.00E-236	1.37E-235
IGFBP3	Insulin-like growth factor-binding protein 3	-0.0016	-0.007 (-0.009, -0.005)	2.60E-13	2.94E-13
IGSF21	Immunoglobulin superfamily member 21	0.0001	0.016 (0.014, 0.018)	2.70E-63	4.16E-63
IL19	Interleukin-19	0.0001	0.013 (0.011, 0.015)	1.30E-37	1.62E-37
IL32	Interleukin-32	-0.0007	-0.014 (-0.016, -0.012)	1.70E-48	2.31E-48
IL3RA	Interleukin-3 receptor subunit alpha	2.52E-05	0.015 (0.014, 0.017)	9.80E-58	1.44E-57
INSL5	Insulin-like peptide INSL5	0.0005	0.022 (0.02, 0.024)	9.70E-125	2.54E-124
ITIH3	Inter-alpha-trypsin inhibitor heavy chain H3	-0.0007	-0.025 (-0.026, -0.023)	4.70E-157	1.55E-156
KIR3DL2	Killer cell immunoglobulin-like receptor 3DL2	-0.0004	-0.004 (-0.006, -0.002)	1.10E-05	1.21E-05
KLK14	Kallikrein-14	-0.0029	-0.017 (-0.019, -0.015)	1.60E-68	2.62E-68
KLK3	Prostate-specific antigen	0.0003	0.046 (0.044, 0.047)	0.00E+00	0.00E+00
LACRT	Extracellular glycoprotein lacritin	-0.0001	-0.012 (-0.014, -0.01)	1.60E-38	2.12E-38
LCAT	Phosphatidylcholine-sterol acyltransferase	0.0011	0.022 (0.02, 0.024)	2.80E-117	6.83E-117
LDLR	Low-density lipoprotein receptor	0.001	0.026 (0.024, 0.028)	6.20E-175	2.42E-174
LEFTY2	Left-right determination factor 2	-0.0005	-0.019 (-0.021, -0.017)	3.10E-94	6.04E-94
LPL	Lipoprotein lipase	-0.0032	-0.037 (-0.038, -0.035)	0.00E+00	0.00E+00
LPO	Lactoperoxidase	-0.0001	-0.003 (-0.005, -0.001)	0.0013	1.42E-03
LRTM2	Leucine-rich repeat and transmembrane domain-containing protein 2	-0.0006	-0.021 (-0.023, -0.019)	1.30E-109	2.84E-109
LUZP2	Leucine zipper protein 2	-2.71E-05	-0.012 (-0.013, -0.01)	1.70E-33	2.12E-33
MAN2B2	Epididymis-specific alpha-mannosidase	0.0006	0.018 (0.017, 0.02)	1.40E-79	2.57E-79
MDGA1	MAM domain-containing glycosylphosphatidylinositol anchor protein 1	-0.0001	-0.013 (-0.015, -0.011)	5.50E-39	7.13E-39
MEP1A	Meprin A subunit alpha	0.0006	0.019 (0.017, 0.021)	1.10E-88	2.11E-88
MET	Hepatocyte growth factor receptor	-0.0016	-0.018 (-0.02, -0.016)	8.00E-80	1.43E-79
MMP3	Stromelysin-1	0.0034	0.035 (0.033, 0.037)	2.40E-307	3.30E-306
MRC1	Macrophage mannose receptor 1	3.56E-06	0.013 (0.011, 0.015)	9.80E-40	1.28E-39
MSMB	Beta-microseminoprotein	0.001	0.018 (0.017, 0.02)	8.70E-79	1.52E-78
NCAN	Neurocan core protein	-0.0005	-0.025 (-0.027, -0.023)	1.80E-152	5.65E-152
NPC2	NPC intracellular cholesterol transporter 2	0.0002	0.019 (0.017, 0.021)	4.80E-74	8.20E-74
NPTX2	Neuronal pentraxin-2	0.0016	0.025 (0.024, 0.027)	5.30E-160	1.87E-159
NTF3	Neurotrophin-3	-0.0013	-0.01 (-0.012, -0.008)	1.70E-26	2.00E-26
NTRK2	BDNF/NT-3 growth factors receptor	0.0007	0.002 (0, 0.004)	0.0157	1.64E-02
OPTC	Opticin	-0.0015	-0.02 (-0.022, -0.018)	9.80E-100	2.05E-99
PCDH9	Protocadherin-9	-0.0003	0.002 (0, 0.004)	0.0665	6.92E-02
PGA4	Pepsin A-4	0.0003	0.007 (0.005, 0.009)	8.00E-12	8.97E-12
PGF	Placenta growth factor	0.0024	0.029 (0.028, 0.031)	2.60E-193	1.19E-192
PI16	Peptidase inhibitor 16	-0.0001	-0.012 (-0.014, -0.01)	4.80E-33	5.97E-33

PI3	Elafin	0.0003	0.022 (0.02, 0.024)	1.60E-115	3.64E-115
PLA2G1B	Phospholipase A2	-0.0021	-0.022 (-0.024, -0.02)	2.90E-114	6.64E-114
PLA2G7	Platelet-activating factor acetylhydrolase	0.0003	0.027 (0.026, 0.029)	2.70E-182	1.19E-181
PLAT	Tissue-type plasminogen activator	0.0023	0.033 (0.031, 0.035)	9.90E-264	9.67E-263
POF1B	Protein POF1B	-0.0004	-0.004 (-0.006, -0.003)	4.40E-06	4.67E-06
PRAP1	Proline-rich acidic protein 1	0.0008	0.04 (0.038, 0.042)	0.00E+00	0.00E+00
PRL	Prolactin	-4.99E-05	-0.014 (-0.015, -0.012)	1.10E-46	1.52E-46
PROK1	Prokineticin-1	0.001	0.031 (0.029, 0.033)	1.80E-257	1.67E-256
PRSS8	Prostasin	0.0031	0.032 (0.03, 0.034)	1.40E-223	9.22E-223
PSPN	Persephin	0.0052	0.041 (0.039, 0.043)	0.00E+00	0.00E+00
PTH	Parathyroid hormone	0.0002	0.02 (0.018, 0.022)	4.10E-93	7.94E-93
PTPRF	Receptor-type tyrosine-protein phosphatase F	0.0001	0.02 (0.018, 0.022)	2.10E-96	4.36E-96
PTPRR	Receptor-type tyrosine-protein phosphatase R	-0.0001	-0.009 (-0.011, -0.007)	1.90E-19	2.23E-19
PTX3	Pentraxin-related protein PTX3	-0.002	-0.014 (-0.016, -0.012)	5.80E-49	8.04E-49
PZP	Pregnancy zone protein	-0.0019	-0.03 (-0.032, -0.029)	3.10E-244	2.45E-243
RBP7	Retinoid-binding protein 7	-0.0004	0.001 (0, 0.003)	0.1294	1.32E-01
REN	Renin	0.0017	0.024 (0.021, 0.026)	5.10E-94	1.00E-93
RTN4R	Reticulon-4 receptor	1.96E-05	0.028 (0.026, 0.03)	2.80E-177	1.14E-176
SCGB1A1	Uteroglobin	-0.0002	-0.002 (-0.004, 0)	0.0703	7.28E-02
SCGB3A1	Secretoglobin family 3A member 1	-1.76E-05	-0.016 (-0.017, -0.014)	1.10E-59	1.69E-59
SCGB3A2	Secretoglobin family 3A member 2	-0.0009	-0.018 (-0.02, -0.016)	1.00E-80	1.83E-80
SCRG1	Scrapie-responsive protein 1	0.0003	0.024 (0.023, 0.026)	1.40E-136	3.86E-136
SELE	E-selectin	0.0021	0.025 (0.023, 0.027)	2.90E-143	8.11E-143
SEMA3F	Semaphorin-3F	0.0012	0.023 (0.021, 0.024)	1.70E-116	4.13E-116
SERPINA11	Serpin A11	-0.0001	-0.028 (-0.03, -0.026)	2.60E-201	1.29E-200
SERPINA6	Corticosteroid-binding globulin	-0.0014	-0.025 (-0.026, -0.023)	2.40E-150	7.32E-150
SETMAR	Histone-lysine N-methyltransferase SETMAR	0.0016	0.027 (0.025, 0.029)	1.20E-180	4.85E-180
SEZ6	Seizure protein 6 homolog	-0.0001	-0.015 (-0.017, -0.013)	2.10E-53	3.02E-53
SGSH	N-sulphoglucosamine sulphohydrolase	0.0002	0.017 (0.015, 0.019)	6.00E-68	9.91E-68
SIGLEC7	Sialic acid-binding Ig-like lectin 7	0.0001	0.014 (0.012, 0.016)	1.30E-43	1.79E-43
SLC39A14	Zinc transporter ZIP14	-0.0002	-0.011 (-0.013, -0.009)	5.70E-31	7.02E-31
SLITRK1	SLIT and NTRK-like protein 1	-0.0015	-0.021 (-0.023, -0.02)	5.50E-113	1.25E-112
SPESP1	Sperm equatorial segment protein 1	0.0002	0.023 (0.022, 0.025)	2.00E-136	5.52E-136
SPINK6	Serine protease inhibitor Kazal-type 6	0.0002	0.022 (0.02, 0.024)	2.30E-118	5.79E-118
SPRR3	Small proline-rich protein 3	-0.0001	-0.016 (-0.018, -0.014)	6.50E-63	1.01E-62
SRPX	Sushi repeat-containing protein SRPX	-0.0009	-0.019 (-0.021, -0.017)	8.20E-84	1.52E-83
SSC4D	Scavenger receptor cysteine-rich domain-containing group B protein	0.0026	0.04 (0.038, 0.042)	0.00E+00	0.00E+00
SSC5D	Soluble scavenger receptor cysteine-rich domain-containing protein SSC5D	0.001	0.025 (0.023, 0.027)	3.40E-147	9.99E-147
STAB2	Stabilin-2	0.0002	0.025 (0.023, 0.027)	2.10E-151	6.59E-151
TEX101	Testis-expressed protein 101	0.0021	0.036 (0.035, 0.038)	0.00E+00	0.00E+00
TFPI2	Tissue factor pathway inhibitor 2	-0.0004	-0.012 (-0.014, -0.01)	1.80E-35	2.25E-35
THBD	Thrombomodulin	0.0019	0.018 (0.017, 0.02)	4.80E-79	8.52E-79

THBS2	Thrombospondin-2	-0.0003	-0.011 (-0.013, -0.009)	2.10E-30	2.61E-30
TNR	Tenascin-R	-0.0002	-0.016 (-0.017, -0.014)	4.20E-60	6.31E-60
TPT1	Translationally-controlled tumor protein	-0.0001	-0.002 (-0.003, 0)	0.1116	1.14E-01
UPB1	Beta-ureidopropionase	0.0001	0.029 (0.027, 0.031)	4.70E-211	2.74E-210
VEGFD	Vascular endothelial growth factor D	-0.0017	-0.023 (-0.025, -0.021)	1.60E-128	4.19E-128
VIT	Vitrin	0.0004	0.031 (0.029, 0.032)	9.20E-241	6.47E-240
VWC2L	von Willebrand factor C domain-containing protein 2-like	-0.0006	-0.019 (-0.021, -0.018)	2.70E-88	5.08E-88
WFDC12	WAP four-disulfide core domain protein 12	0.0023	0.029 (0.027, 0.03)	1.50E-201	7.73E-201
WIF1	Wnt inhibitory factor 1	-0.0027	-0.017 (-0.019, -0.015)	5.30E-70	9.04E-70
XG	Glycoprotein Xg	-0.0043	-0.029 (-0.031, -0.027)	7.40E-199	3.50E-198
ZHX2	Zinc fingers and homeoboxes protein 2	2.77E-05	0.015 (0.013, 0.017)	1.80E-59	2.74E-59

CI: confidence interval; FDR: false discovery rate.
*Linear regressions of the measured waist-hip ratio regressed on each of the proteins (per standard deviation).

Supplemental Table S2D. The 25 LASSO Selected Proteins Shared across Obesity-related Phenotypes.

Protein	Name	BMI			Body Fat Percentage			Waist-hip Ratio		
		LASSO Beta	Linear Regression*		LASSO Beta	Linear Regression*		LASSO Beta	Linear Regression*	
			Beta (95% CI)	P		Beta (95% CI)	P		Beta (95% CI)	P
ADGRG2	Adhesion G-protein coupled receptor G2	-0.1181	-0.97 (-1.06, -0.88)	3.60E-92	-0.0482	0.69 (0.5, 0.88)	5.40E-13	-0.003	-0.029 (-0.031, -0.027)	9.20E-211
AGER	Advanced glycosylation end product-specific receptor	-0.1281	-0.84 (-0.93, -0.74)	1.60E-68	-0.0398	-0.24 (-0.43, -0.05)	0.0136	-0.0004	-0.014 (-0.016, -0.012)	8.90E-50
BAG3	BAG family molecular chaperone regulator 3	0.0124	1.1 (1, 1.19)	1.40E-115	-0.0041	0.49 (0.3, 0.68)	5.60E-07	0.0001	0.017 (0.015, 0.018)	4.80E-65
CD300LG	CMRF35-like molecule 9	0.1124	-0.01 (-0.11, 0.08)	0.7624	0.1262	1.46 (1.27, 1.65)	2.30E-51	-0.0056	-0.023 (-0.025, -0.021)	5.20E-130
CD99	CD99 antigen	0.0552	0.66 (0.56, 0.75)	8.90E-42	-0.1061	-2 (-2.19, -1.82)	4.90E-97	0.0045	0.03 (0.028, 0.031)	1.20E-214
CFH	Complement factor H	0.1082	1.84 (1.75, 1.93)	9.9e-324	0.0384	2.4 (2.22, 2.59)	2.30E-132	0.0005	0.026 (0.024, 0.028)	7.40E-154
CHGB	Secretogranin-1	-0.0551	-0.97 (-1.06, -0.87)	2.00E-87	-0.0457	-0.98 (-1.17, -0.79)	2.90E-23	-0.001	-0.009 (-0.011, -0.007)	7.70E-21
COL4A1	Collagen alpha-1(IV) chain	-0.1903	-1.27 (-1.37, -1.18)	1.10E-157	-0.2023	-1.46 (-1.65, -1.27)	1.30E-51	-0.002	-0.02 (-0.022, -0.019)	3.30E-100
CTBS	Di-N-acetylchitobiase	-0.002	0.68 (0.58, 0.77)	2.10E-44	-0.083	-0.05 (-0.25, 0.14)	0.5753	0.0007	0.021 (0.02, 0.023)	1.80E-109
CTHRC1	Collagen triple helix repeat-containing protein 1	0.1693	1.2 (1.11, 1.29)	7.00E-134	-0.0993	-0.83 (-1.02, -0.64)	4.40E-17	2.11E-06	0.028 (0.026, 0.03)	6.80E-182
DMP1	Dentin matrix acidic phosphoprotein 1	-0.0295	-0.66 (-0.75, -0.56)	6.20E-42	0.0545	-0.03 (-0.22, 0.16)	0.7496	-0.0004	-0.017 (-0.019, -0.015)	2.80E-67
DPT	Dermatopontin	0.0935	1.35 (1.26, 1.44)	1.40E-172	0.1813	2.08 (1.89, 2.27)	5.50E-102	0.0043	0.016 (0.014, 0.018)	2.30E-61
ENPP6	Glycerophosphocholine cholinephosphodiesterase ENPP6	-0.047	-1.15 (-1.24, -1.07)	1.40E-136	-0.1154	-0.74 (-0.92, -0.55)	5.60E-15	-0.0002	-0.019 (-0.021, -0.018)	4.40E-95
FGL1	Fibrinogen-like protein 1	-0.0523	-0.63 (-0.73, -0.54)	3.00E-40	-0.0181	1.53 (1.35, 1.72)	2.20E-58	-0.0006	-0.025 (-0.027, -0.024)	6.90E-159
GHRL	Appetite-regulating hormone	-0.0668	-1.02 (-1.11, -0.93)	6.90E-101	-0.0548	0.34 (0.15, 0.53)	0.0004	-0.0008	-0.022 (-0.023, -0.02)	1.50E-112
GPD1	Glycerol-3-phosphate dehydrogenase	0.0752	1.67 (1.58, 1.75)	4.70E-284	0.0394	2.27 (2.08, 2.45)	2.90E-127	0.0004	0.022 (0.02, 0.024)	8.40E-119
NPTX2	Neuronal pentraxin-2	-0.0024	-0.2 (-0.29, -0.1)	3.40E-05	-0.1173	-3.25 (-3.42, -3.07)	3.40E-271	0.0016	0.025 (0.024, 0.027)	5.30E-160
NTRK2	BDNF/NT-3 growth factors receptor	0.1347	0.57 (0.48, 0.66)	2.50E-32	0.0757	1.25 (1.06, 1.44)	7.20E-39	0.0007	0.002 (0, 0.004)	0.0157
OPTC	Opticin	-0.2481	-0.91 (-1, -0.82)	2.00E-81	-0.0097	0.56 (0.37, 0.75)	5.40E-09	-0.0015	-0.02 (-0.022, -0.018)	9.80E-100
PLA2G1B	Phospholipase A2	-0.0326	-0.98 (-1.07, -0.89)	1.20E-90	-0.0466	0.02 (-0.17, 0.21)	0.8165	-0.0021	-0.022 (-0.024, -0.02)	2.90E-114
SCGB3A1	Secretoglobin family 3A member 1	-0.0528	-0.78 (-0.87, -0.69)	2.70E-60	-0.0767	0.13 (-0.06, 0.32)	0.176	-1.76E-05	-0.016 (-0.017, -0.014)	1.10E-59
SCGB3A2	Secretoglobin family 3A member 2	-0.073	-1.22 (-1.31, -1.13)	1.80E-146	-0.0432	-0.92 (-1.1, -0.73)	1.50E-21	-0.0009	-0.018 (-0.02, -0.016)	1.00E-80
SPINK6	Serine protease inhibitor Kazal-type 6	0.006	0.79 (0.7, 0.88)	3.90E-62	-0.1922	-0.54 (-0.73, -0.35)	1.40E-08	0.0002	0.022 (0.02, 0.024)	2.30E-118

SSC4D	Scavenger receptor cysteine-rich domain-containing group B protein	0.0317	1.75 (1.66, 1.83)	5.9e-312	-0.0092	0.02 (-0.17, 0.21)	0.8352	0.0026	0.04 (0.038, 0.042)	0.00E+00
VWC2L	von Willebrand factor C domain-containing protein 2-like	-0.1231	-1.03 (-1.13, -0.94)	4.90E-100	-0.0068	0.23 (0.04, 0.42)	0.019	-0.0006	-0.019 (-0.021, -0.018)	2.70E-88

CI: confidence interval.
*Linear regressions of the measured obesity-related phenotype regressed on each of the proteins (per standard deviation).

Supplemental Table S3A. Pathway Enrichment for Gene Ontology Using the 389 LASSO-selected Proteins for BMI. Top 10 Pathways out of Total 86 Pathways with p < 0.05 are shown.

GO.ID	Term	Annotated	Significant	Expected	P	FDR
GO:0007155	cell adhesion	581	103	77.16	0.0005	1
GO:0006508	proteolysis	400	58	53.12	0.0005	1
GO:0051965	positive regulation of synapse assembly	22	9	2.92	0.0012	1
GO:0032696	negative regulation of interleukin-13 production	5	4	0.66	0.0014	1
GO:0051838	cytolysis by host of symbiont cells	5	4	0.66	0.0014	1
GO:0014009	glial cell proliferation	23	5	3.05	0.0023	1
GO:0099560	synaptic membrane adhesion	9	5	1.2	0.0032	1
GO:0031214	biomineral tissue development	47	11	6.24	0.0036	1
GO:0010460	positive regulation of heart rate	6	4	0.8	0.0037	1
GO:0071310	cellular response to organic substance	541	75	71.85	0.0083	1

Supplemental Table S3B. Pathway Enrichment for Gene Ontology Using the 385 LASSO-selected Proteins for Body Fat Percentage. Top 10 Pathways out of Total 75 Pathways with p < 0.05 are shown.

GO.ID	Term	Annotated	Significant	Expected	P	FDR
GO:0007155	cell adhesion	581	100	76.95	3.50E-07	0.0039
GO:0030198	extracellular matrix organization	123	37	16.29	9.00E-06	0.0507
GO:0006508	proteolysis	400	64	52.98	6.20E-05	0.2328
GO:0032526	response to retinoic acid	27	9	3.58	0.0007	1
GO:0038063	collagen-activated tyrosine kinase receptor signaling pathway	3	3	0.4	0.0023	1
GO:0042104	positive regulation of activated T cell proliferation	12	6	1.59	0.0024	1
GO:0006954	inflammatory response	329	57	43.57	0.0040	1
GO:0051603	proteolysis involved in protein catabolic process	117	10	15.5	0.0070	1
GO:0043129	surfactant homeostasis	7	4	0.93	0.0076	1
GO:0014012	peripheral nervous system axon regeneration	4	3	0.53	0.0083	1

Supplemental Table S3C. Pathway Enrichment for Gene Ontology Using the 176 LASSO-selected Proteins for Waist-hip Ratio. Top 10 Pathways out of Total 57 Pathways with p < 0.05 are shown.

GO.ID	Term	Annotated	Significant	Expected	P	FDR
GO:0007155	cell adhesion	581	49	35.22	0.0003	1
GO:0046326	positive regulation of glucose import	8	4	0.49	0.0008	1
GO:0042756	drinking behavior	3	3	0.18	0.0036	1
GO:0044871	negative regulation by host of viral glycoprotein metabolic process	2	2	0.12	0.0037	1
GO:1903016	negative regulation of exo-alpha-sialidase activity	2	2	0.12	0.0037	1
GO:0019695	choline metabolic process	2	2	0.12	0.0037	1
GO:0090675	intermicrovillar adhesion	2	2	0.12	0.0037	1
GO:0044869	negative regulation by host of viral exo-alpha-sialidase activity	2	2	0.12	0.0037	1
GO:0050805	negative regulation of synaptic transmission	16	5	0.97	0.0037	1
GO:0019886	antigen processing and presentation of exogenous peptide antigen via MHC class II	12	4	0.73	0.0044	1

Supplemental Table S4. Associations Between Protein Predicted Scores and Outcomes. Sensitivity Analysis Results after Excluding Cancer at Baseline.

Outcome	Model	PPS_{BMI} (per SD)				PPS_{BFP} (per SD)				PPS_{WHR} (per SD)			
		N	N Event	HR (95% CI)	P	N	N Event	HR (95% CI)	P	N	N Event	HR (95% CI)	P
MACE	Model 1	29032	3586	1.22 (1.18, 1.26)	**<.0001**	28604	3501	1.29 (1.23, 1.35)	**<.0001**	29081	3597	1.46 (1.39, 1.54)	**<.0001**
	Model 2	29032	3586	1.15 (1.07, 1.23)	**0.0002**	28604	3501	1.33 (1.22, 1.46)	**<.0001**	29081	3597	1.33 (1.25, 1.42)	**<.0001**
	Model 3	23476	2897	1.05 (0.97, 1.14)	0.229	23148	2837	1.21 (1.09, 1.33)	**0.0003**	23513	2906	1.12 (1.03, 1.22)	**0.0099**
Ischemic Stroke	Model 1	29031	691	1.14 (1.05, 1.23)	**0.0016**	28604	683	1.18 (1.06, 1.32)	**0.0027**	29080	695	1.32 (1.16, 1.49)	**<.0001**
	Model 2	29031	691	1.07 (0.92, 1.26)	0.3819	28604	683	1.05 (0.87, 1.28)	0.5963	29080	695	1.26 (1.08, 1.47)	**0.0027**
	Model 3	23476	572	1.07 (0.89, 1.28)	0.4926	23148	568	0.94 (0.75, 1.17)	0.5474	23513	574	1.19 (0.97, 1.46)	0.0935
MI	Model 1	29032	2743	1.23 (1.19, 1.28)	**<.0001**	28604	2679	1.31 (1.24, 1.38)	**<.0001**	29081	2745	1.52 (1.43, 1.62)	**<.0001**
	Model 2	29032	2743	1.21 (1.12, 1.31)	**<.0001**	28604	2679	1.37 (1.24, 1.52)	**<.0001**	29081	2745	1.4 (1.29, 1.51)	**<.0001**
	Model 3	23476	2215	1.09 (1, 1.2)	0.0649	23148	2167	1.24 (1.1, 1.39)	**0.0003**	23513	2217	1.14 (1.03, 1.26)	**0.0086**
CV Death	Model 1	29032	828	1.3 (1.2, 1.41)	**<.0001**	28604	789	1.44 (1.29, 1.62)	**<.0001**	29081	834	1.43 (1.27, 1.62)	**<.0001**
	Model 2	29032	828	1.01 (0.86, 1.18)	0.8971	28604	789	1.56 (1.27, 1.9)	**<.0001**	29081	834	1.19 (1.03, 1.37)	**0.0192**
	Model 3	23476	662	0.97 (0.82, 1.16)	0.7492	23148	633	1.39 (1.11, 1.75)	**0.0046**	23513	667	1.09 (0.91, 1.31)	0.3312

PPS_{BMI}, protein predicted score of BMI; PPS_{BFP}: protein predicted score of body fat percentage; PPS_{WHR}: protein predicted score of waist-hip ratio; MACE: major adverse cardiovascular events (ischemic stroke, myocardial infarction, and cardiovascular death); MI: myocardial infarction; CV: cardiovascular; HR: hazard ratio; CI: confidence interval.

Model 1: adjusted for age, sex, race (white vs. other);

Model 2: adjusted for the measured obesity-related phenotype (BMI, body fat percentage, or waist-hip ratio) in addition to Model 1;

Model 3: adjusted for total cholesterol, high density lipoprotein cholesterol, systolic blood pressure, estimated glomerular filtration rate calculated using the 2021 CKD-EPI equation, diabetes, current smoking, blood pressure lowering medication use, cholesterol lowering medication use in addition to Model 2.

Supplemental Table S5. Sex-Specific Associations Between Protein Predicted Scores and Outcomes.

Protein Predicted Score	Outcome	Model	Male				Female				Sex Interaction P
			N	N Event	HR (95% CI)	P	N	N Event	HR (95% CI)	P	
PPS_{BMI} (per SD)	MACE	Model 1	14927	2489	1.26 (1.21, 1.32)	**<.0001**	17830	1568	1.17 (1.11, 1.23)	**<.0001**	**0.018**
		Model 2	14927	2489	1.21 (1.11, 1.32)	**<.0001**	17830	1568	1.08 (0.97, 1.19)	0.1721	**0.0127**
		Model 3	12141	2016	1.13 (1.02, 1.25)	**0.0159**	14309	1255	1.01 (0.89, 1.14)	0.8875	0.3764
	Ischemic Stroke	Model 1	14927	427	1.13 (1.01, 1.26)	**0.0256**	17828	349	1.14 (1.03, 1.27)	**0.0103**	0.9263
		Model 2	14927	427	1.04 (0.84, 1.27)	0.7402	17828	349	1.18 (0.94, 1.47)	0.1469	0.9494
		Model 3	12141	351	1.04 (0.82, 1.32)	0.7535	14309	289	1.17 (0.92, 1.5)	0.2054	0.3422
	MI	Model 1	14927	1984	1.27 (1.21, 1.33)	**<.0001**	17830	1109	1.19 (1.12, 1.26)	**<.0001**	0.0681
		Model 2	14927	1984	1.28 (1.16, 1.41)	**<.0001**	17830	1109	1.1 (0.97, 1.25)	0.1322	0.0612
		Model 3	12141	1610	1.17 (1.04, 1.31)	**0.007**	14309	886	1.03 (0.89, 1.19)	0.7113	0.4282
	CV Death	Model 1	14927	622	1.33 (1.2, 1.46)	**<.0001**	17830	348	1.21 (1.08, 1.36)	**0.0011**	0.2363
		Model 2	14927	622	1.17 (0.97, 1.41)	0.1047	17830	348	0.86 (0.68, 1.09)	0.222	0.1578
		Model 3	12141	481	1.17 (0.95, 1.43)	0.1421	14309	286	0.84 (0.64, 1.09)	0.1836	0.7397
PPS_{BFP} (per SD)	MACE	Model 1	14692	2428	1.35 (1.27, 1.43)	**<.0001**	17576	1524	1.21 (1.13, 1.3)	**<.0001**	**0.0457**
		Model 2	14692	2428	1.36 (1.22, 1.51)	**<.0001**	17576	1524	1.33 (1.15, 1.53)	**<.0001**	0.0548
		Model 3	11954	1971	1.26 (1.12, 1.42)	**0.0001**	14120	1225	1.25 (1.06, 1.46)	**0.0069**	0.3887
	Ischemic Stroke	Model 1	14692	423	1.24 (1.07, 1.43)	**0.0038**	17575	344	1.14 (0.98, 1.33)	0.0836	0.5298
		Model 2	14692	423	1.13 (0.89, 1.45)	0.319	17575	344	1.1 (0.83, 1.46)	0.515	0.5023
		Model 3	11954	349	1.05 (0.79, 1.38)	0.7474	14120	287	0.99 (0.73, 1.34)	0.9405	0.8778
	MI	Model 1	14692	1937	1.34 (1.26, 1.43)	**<.0001**	17576	1078	1.26 (1.16, 1.37)	**<.0001**	0.3371
		Model 2	14692	1937	1.4 (1.24, 1.57)	**<.0001**	17576	1078	1.35 (1.15, 1.59)	**0.0003**	0.3805
		Model 3	11954	1574	1.28 (1.12, 1.46)	**0.0003**	14120	864	1.28 (1.07, 1.55)	**0.0087**	0.7546
	CV Death	Model 1	14692	591	1.53 (1.34, 1.75)	**<.0001**	17576	333	1.19 (1, 1.42)	**0.0462**	**0.0344**
		Model 2	14692	591	1.69 (1.37, 2.1)	**<.0001**	17576	333	1.24 (0.88, 1.74)	0.2285	**0.04**
		Model 3	11954	457	1.58 (1.23, 2.02)	**0.0003**	14120	275	1.16 (0.79, 1.71)	0.4391	0.324
PPS_{WHR} (per SD)	MACE	Model 1	14966	2500	1.52 (1.42, 1.63)	**<.0001**	17850	1570	1.4 (1.29, 1.51)	**<.0001**	0.1787
		Model 2	14966	2500	1.34 (1.24, 1.46)	**<.0001**	17850	1570	1.32 (1.2, 1.46)	**<.0001**	0.146
		Model 3	12168	2026	1.15 (1.03, 1.27)	**0.0139**	14327	1256	1.14 (1, 1.29)	**0.0476**	0.4153
		Model 1	14966	430	1.32 (1.12, 1.55)	**0.0009**	17848	351	1.31 (1.11, 1.55)	**0.0013**	0.9464

	**Ischemic Stroke**	**Model 2**	14966	430	1.14 (0.94, 1.39)	0.1805	17848	351	1.42 (1.15, 1.74)	**0.001**	0.9644
		**Model 3**	12168	353	1.08 (0.82, 1.41)	0.5821	14327	290	1.34 (1.02, 1.75)	**0.0345**	0.6546
	**MI**	**Model 1**	14966	1986	1.55 (1.43, 1.67)	**<.0001**	17850	1109	1.48 (1.36, 1.62)	**<.0001**	0.6109
		**Model 2**	14966	1986	1.42 (1.29, 1.56)	**<.0001**	17850	1109	1.38 (1.23, 1.54)	**<.0001**	0.5559
		**Model 3**	12168	1612	1.16 (1.03, 1.3)	**0.0165**	14327	886	1.17 (1.01, 1.35)	**0.0413**	0.8301
	**CV Death**	**Model 1**	14966	630	1.4 (1.21, 1.61)	**<.0001**	17850	348	1.45 (1.21, 1.74)	**<.0001**	0.6127
		**Model 2**	14966	630	1.08 (0.91, 1.27)	0.3814	17850	348	1.31 (1.06, 1.63)	**0.0137**	0.6991
		**Model 3**	12168	488	1.06 (0.85, 1.31)	0.6094	14327	286	1.12 (0.86, 1.48)	0.4018	0.6826

PPS_{BMI}, protein predicted score of BMI; PPS_{BFP}: protein predicted score of body fat percentage; PPS_{WHR}: protein predicted score of waist-hip ratio; MACE: major adverse cardiovascular events (ischemic stroke, myocardial infarction, and cardiovascular death); MI: myocardial infarction; CV: cardiovascular; HR: hazard ratio; CI: confidence interval.

Model 1: adjusted for age, race (white vs. other);

Model 2: adjusted for the measured obesity-related phenotype (BMI, body fat percentage, or waist-hip ratio) in addition to Model 1;

Model 3: adjusted for total cholesterol, high density lipoprotein cholesterol, systolic blood pressure, estimated glomerular filtration rate calculated using the 2021 CKD-EPI equation, diabetes, current smoking, blood pressure lowering medication use, cholesterol lowering medication use in addition to Model 2.

Supplemental Table S6. Sex-Specific Associations Between Protein Predicted Scores and Outcomes. Sensitivity Analysis Results after Excluding Cancer at Baseline.

Protein Predicted Score	Outcome	Model	Male				Female				Sex Interaction P
			N	N Event	HR (95% CI)	P	N	N Event	HR (95% CI)	P	
PPS$_{BMI}$ (per SD)	MACE	**Model 1**	13738	2250	1.26 (1.2, 1.32)	**<.0001**	15294	1336	1.17 (1.11, 1.23)	**<.0001**	**0.03**
		**Model 2**	13738	2250	1.19 (1.09, 1.31)	**0.0002**	15294	1336	1.07 (0.96, 1.19)	0.243	**0.0208**
		**Model 3**	11175	1825	1.1 (0.99, 1.23)	0.0754	12301	1072	0.98 (0.86, 1.12)	0.7712	0.3409
	Ischemic Stroke	**Model 1**	13738	388	1.11 (0.99, 1.25)	0.0662	15293	303	1.15 (1.03, 1.29)	**0.01**	0.6829
		**Model 2**	13738	388	0.96 (0.77, 1.19)	0.7029	15293	303	1.21 (0.95, 1.54)	0.1169	0.7228
		**Model 3**	11175	323	0.95 (0.74, 1.22)	0.691	12301	249	1.21 (0.92, 1.58)	0.1669	0.2
	MI	**Model 1**	13738	1797	1.27 (1.21, 1.33)	**<.0001**	15294	946	1.19 (1.12, 1.26)	**<.0001**	0.0816
		**Model 2**	13738	1797	1.28 (1.16, 1.42)	**<.0001**	15294	946	1.09 (0.96, 1.25)	0.1828	0.0743
		**Model 3**	11175	1456	1.16 (1.03, 1.3)	**0.0146**	12301	759	0.99 (0.85, 1.16)	0.8958	0.3378
	CV Death	**Model 1**	13738	549	1.31 (1.18, 1.46)	**<.0001**	15294	279	1.28 (1.13, 1.45)	**0.0001**	0.7621
		**Model 2**	13738	549	1.1 (0.9, 1.35)	0.3272	15294	279	0.87 (0.68, 1.11)	0.2641	0.5688
		**Model 3**	11175	424	1.09 (0.88, 1.36)	0.4196	12301	238	0.81 (0.61, 1.08)	0.1507	0.9832
PPS$_{BFP}$ (per SD)	MACE	**Model 1**	13522	2199	1.35 (1.27, 1.43)	**<.0001**	15082	1302	1.21 (1.13, 1.31)	**<.0001**	0.0567
		**Model 2**	13522	2199	1.34 (1.2, 1.5)	**<.0001**	15082	1302	1.3 (1.12, 1.51)	**0.0006**	0.0629
		**Model 3**	11004	1788	1.24 (1.09, 1.41)	**0.0008**	12144	1049	1.16 (0.98, 1.37)	0.0825	0.2368
	Ischemic Stroke	**Model 1**	13522	384	1.22 (1.05, 1.42)	**0.0101**	15082	299	1.15 (0.98, 1.34)	0.0965	0.6012
		**Model 2**	13522	384	1.06 (0.82, 1.38)	0.6345	15082	299	1.03 (0.76, 1.4)	0.8309	0.5513
		**Model 3**	11004	321	0.98 (0.73, 1.31)	0.8725	12144	247	0.9 (0.65, 1.26)	0.5547	0.8359
	MI	**Model 1**	13522	1756	1.34 (1.25, 1.44)	**<.0001**	15082	923	1.26 (1.16, 1.38)	**<.0001**	0.3777
		**Model 2**	13522	1756	1.4 (1.24, 1.59)	**<.0001**	15082	923	1.3 (1.1, 1.55)	**0.0026**	0.4093
		**Model 3**	11004	1424	1.27 (1.1, 1.46)	**0.001**	12144	743	1.19 (0.98, 1.45)	0.0818	0.5597
	CV Death	**Model 1**	13522	523	1.55 (1.34, 1.78)	**<.0001**	15082	266	1.28 (1.05, 1.56)	**0.0127**	0.1531
		**Model 2**	13522	523	1.72 (1.37, 2.15)	**<.0001**	15082	266	1.26 (0.85, 1.87)	0.2469	0.1656
		**Model 3**	11004	405	1.6 (1.23, 2.08)	**0.0004**	12144	228	1.06 (0.69, 1.63)	0.7772	0.3781
PPS$_{WHR}$ (per SD)	MACE	**Model 1**	13770	2259	1.51 (1.4, 1.62)	**<.0001**	15311	1338	1.4 (1.29, 1.52)	**<.0001**	0.2402
		**Model 2**	13770	2259	1.32 (1.21, 1.45)	**<.0001**	15311	1338	1.34 (1.21, 1.49)	**<.0001**	0.2034
		**Model 3**	11197	1833	1.13 (1.01, 1.26)	**0.0375**	12316	1073	1.1 (0.96, 1.26)	0.1674	0.406

	**Ischemic Stroke**	**Model 1**	13770	390	1.29 (1.08, 1.53)	**0.0044**	15310	305	1.35 (1.13, 1.61)	**0.0011**	0.6877
		**Model 2**	13770	390	1.08 (0.87, 1.33)	0.4876	15310	305	1.52 (1.22, 1.9)	**0.0002**	0.7021
		**Model 3**	11197	324	1.03 (0.77, 1.38)	0.8201	12316	250	1.37 (1.03, 1.82)	**0.0321**	0.5148
	**MI**	**Model 1**	13770	1799	1.54 (1.42, 1.68)	**<.0001**	15311	946	1.48 (1.34, 1.63)	**<.0001**	0.5954
		**Model 2**	13770	1799	1.41 (1.28, 1.55)	**<.0001**	15311	946	1.36 (1.21, 1.54)	**<.0001**	0.5448
		**Model 3**	11197	1458	1.15 (1.01, 1.3)	**0.0315**	12316	759	1.12 (0.95, 1.31)	0.1733	0.7572
	**CV Death**	**Model 1**	13770	555	1.36 (1.17, 1.59)	**<.0001**	15311	279	1.52 (1.25, 1.86)	**<.0001**	0.2809
		**Model 2**	13770	555	1.05 (0.88, 1.26)	0.5901	15311	279	1.45 (1.14, 1.84)	**0.0023**	0.3256
		**Model 3**	11197	429	1.06 (0.85, 1.33)	0.5969	12316	238	1.15 (0.86, 1.54)	0.3532	0.4266

PPS_{BMI}, protein predicted score of BMI; PPS_{BFP}: protein predicted score of body fat percentage; PPS_{WHR}: protein predicted score of waist-hip ratio; MACE: major adverse cardiovascular events (ischemic stroke, myocardial infarction, and cardiovascular death); MI: myocardial infarction; CV: cardiovascular; HR: hazard ratio; CI: confidence interval.

Model 1: adjusted for age, race (white vs. other);

Model 2: adjusted for the measured obesity-related phenotype (BMI, body fat percentage, or waist-hip ratio) in addition to Model 1;

Model 3: adjusted for total cholesterol, high density lipoprotein cholesterol, systolic blood pressure, estimated glomerular filtration rate calculated using the 2021 CKD-EPI equation, diabetes, current smoking, blood pressure lowering medication use, cholesterol lowering medication use in addition to Model 2.

Supplemental Figure S1. Description of the sample selection workflow from the UK Biobank cohort.

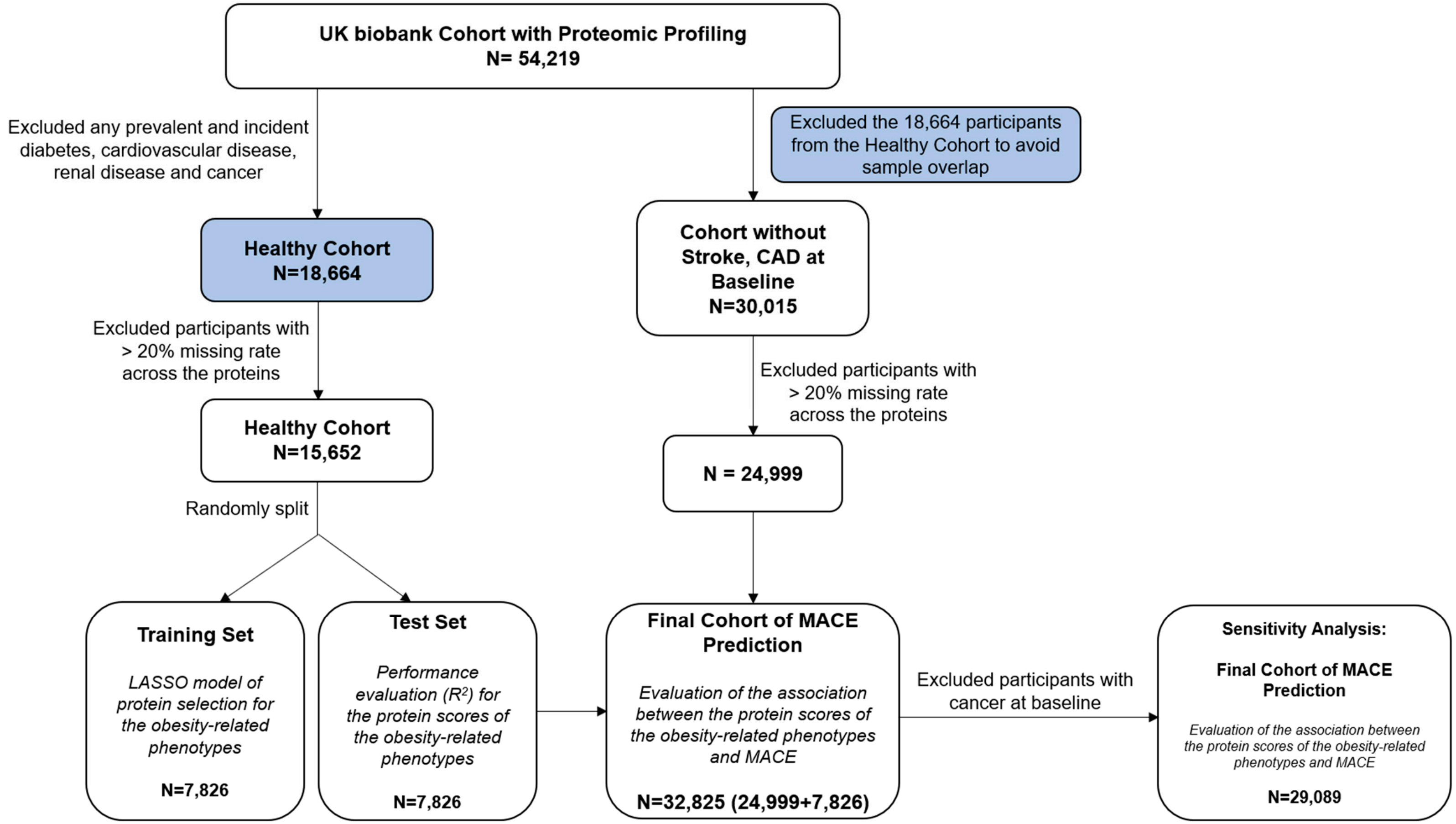

Supplemental Figure S2. R^2 values assessing the prediction performance of protein-predicted scores of BMI (PPS_{BMI}), BFP (PPS_{BFP}), and WHR (PPS_{WHR}) across various sample sizes. The median R^2 from the LASSO models with 2.5% and 97.5% percentiles of the 100 iterations are shown.

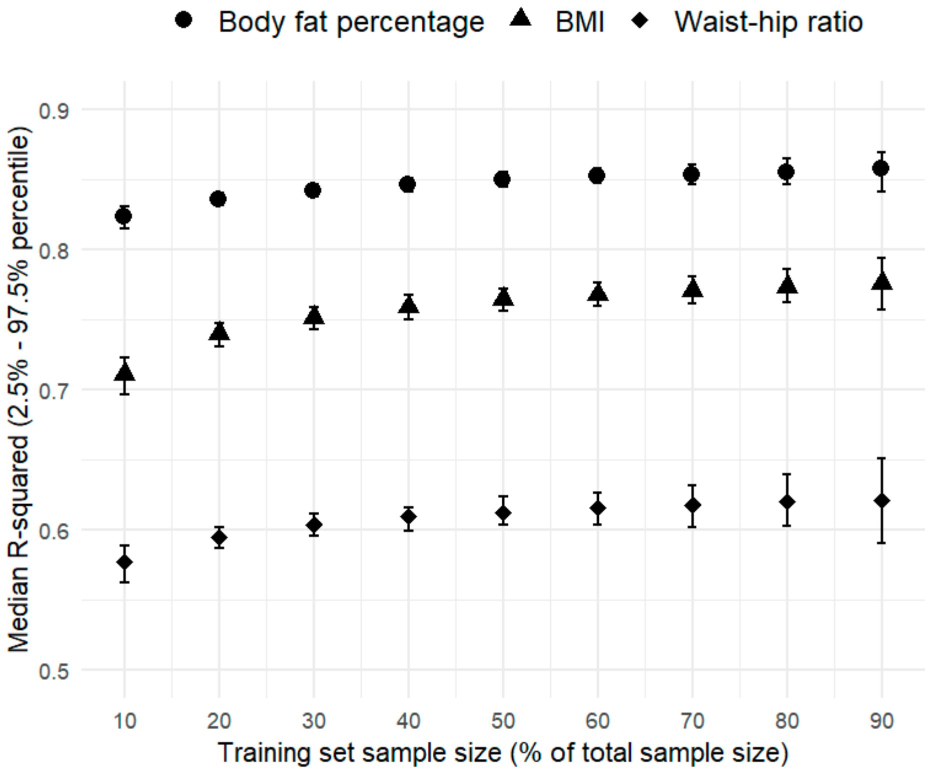

Supplemental Figure S3. The LASSO Selected Proteins Shared across Obesity-related Phenotypes.

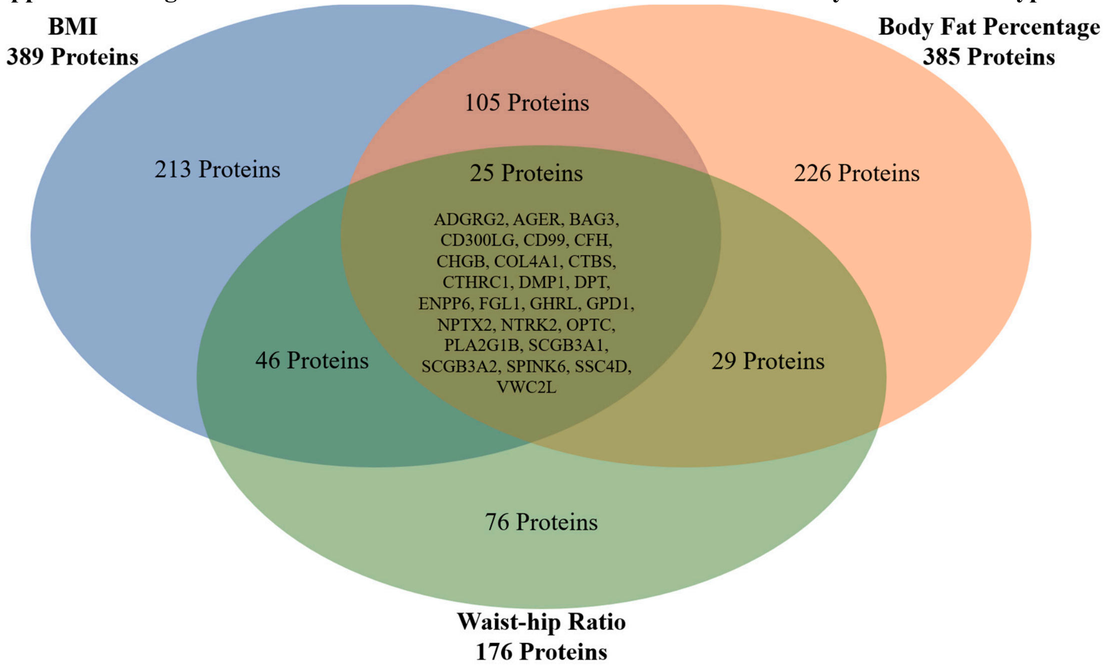

Supplemental Figure S4. Linear Associations Between Predicted Protein Scores of Obesity-related Phenotypes and Measured Phenotypes.

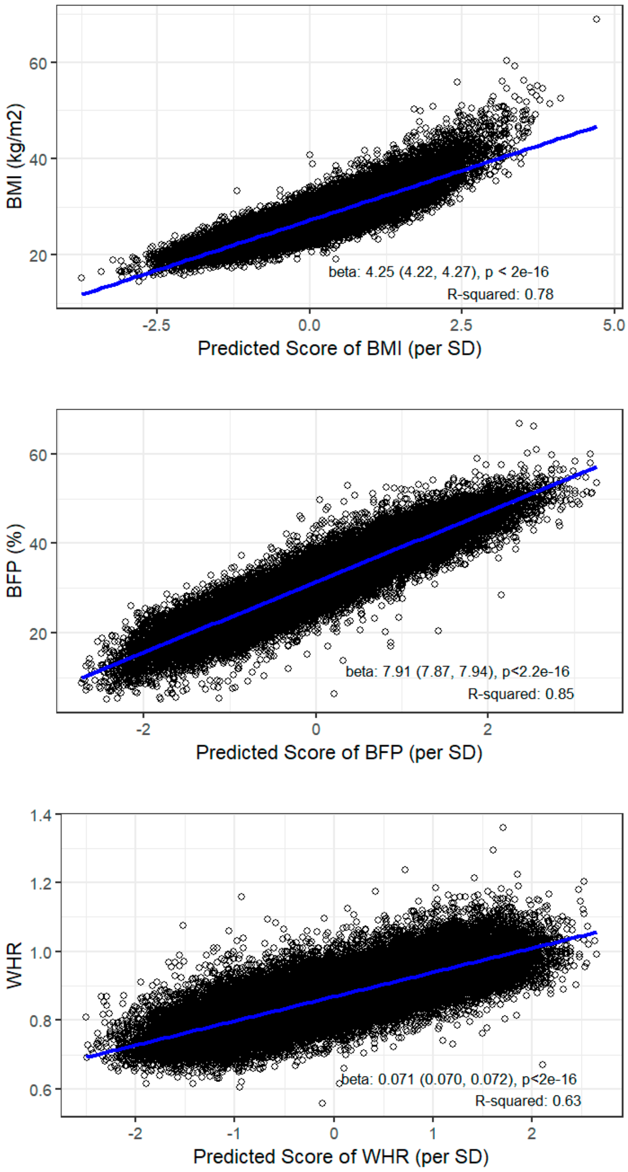

Supplemental Figure S5. Forest Plot of the Associations Between Protein Predicted Scores of Obesity-related Phenotypes and MACE Individual Components. Model 1: adjusted for age, sex and race (white vs. other); Model 2: adjusted for the measured obesity-related phenotype (BMI, body fat percentage, or waist-hip ratio) in addition to Model 1; Model 3: adjusted for total cholesterol, high density lipoprotein cholesterol, systolic blood pressure, estimated glomerular filtration rate calculated using the 2021 CKD-EPI equation, diabetes, current smoking, blood pressure lowering medication use, cholesterol lowering medication use in addition to Model 2.

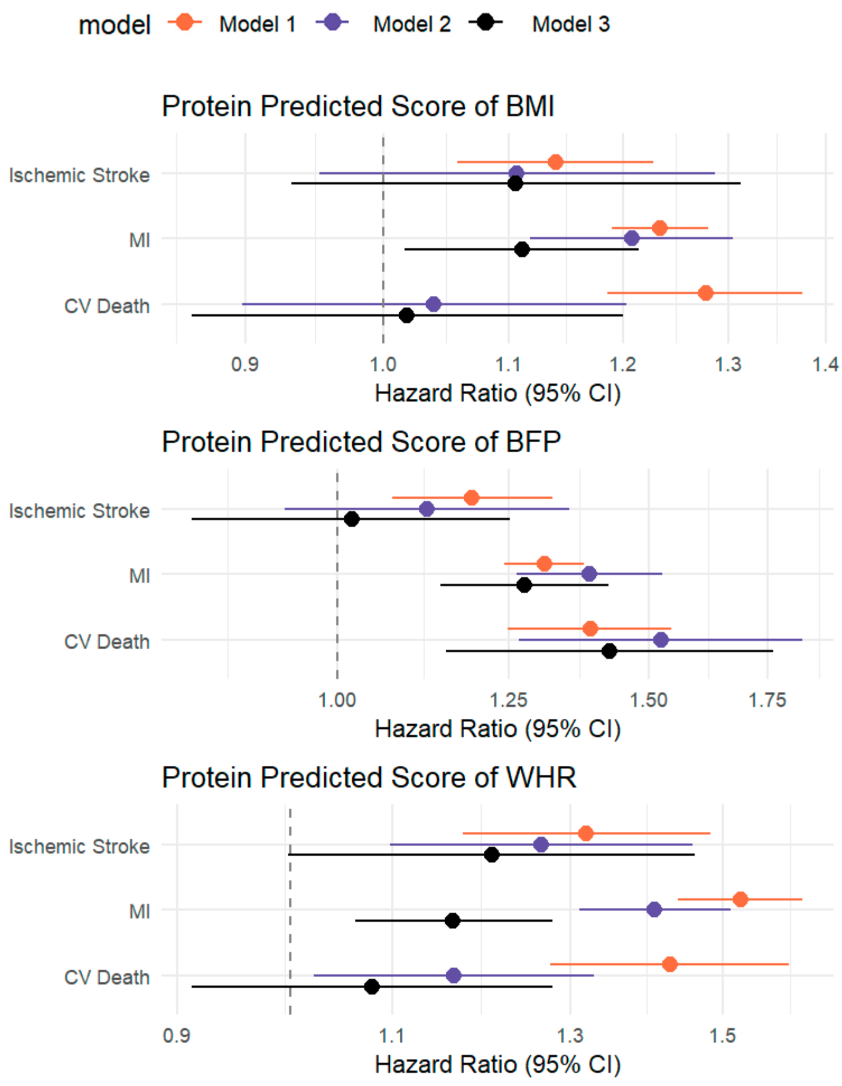

Supplement: Supplementary file 1 [file proteomes-13-00051-s001.zip › proteomes-3863792-supplementary.pdf]
